# Advancing quantitative PCR with color cycle multiplex amplification

**DOI:** 10.1093/nar/gkae683

**Published:** 2024-08-09

**Authors:** Wei Chen, Kerou Zhang, Fei Huang, Lan Zhao, George C Waldren, Qi Jiang, Sherry X Chen, Bonnie Wang, Wei Guo, David Y Zhang, Jinny X Zhang

**Affiliations:** Department of Innovation, NuProbe USA, Houston, TX 77054, USA; Department of Innovation, NuProbe USA, Houston, TX 77054, USA; Department of Laboratory Medicine, Zhongshan Hospital, Fudan University, Shanghai, Shanghai 200032, China; Department of Respiratory and Critical Care Medicine, Shanghai Pulmonary Hospital, School of Medicine, Tongji University, Shanghai 200433, China; Department of Innovation, NuProbe USA, Houston, TX 77054, USA; Department of Innovation, NuProbe USA, Houston, TX 77054, USA; Department of Innovation, NuProbe USA, Houston, TX 77054, USA; Department of Innovation, NuProbe USA, Houston, TX 77054, USA; Department of Laboratory Medicine, Zhongshan Hospital, Fudan University, Shanghai, Shanghai 200032, China; Department of Innovation, NuProbe USA, Houston, TX 77054, USA; Department of Innovation, NuProbe USA, Houston, TX 77054, USA

## Abstract

Quantitative PCR (qPCR) is the gold standard for detection and quantitation of known DNA targets, but the scarcity of spectrally distinct fluorophores and filter sets limits the number of detectable targets. Here, we introduce color cycle multiplex amplification (CCMA) to significantly increase the number of detectable DNA targets in a single qPCR reaction using standard instrumentation. In CCMA, presence of one DNA target species results in a pre-programmed pattern of fluorescence increases. This pattern is distinguished by cycle thresholds (Cts) through rationally designed delays in amplification. For example, we design an assay wherein *Staphylococcus aureus* sequentially induces FAM, then Cy5.5, then ROX fluorescence increases with more than 3 cycles between each signal. CCMA offers notably higher potential for multiplexing because it uses fluorescence permutation rather than combination. With 4 distinct fluorescence colors, CCMA theoretically allows the detection of up to 136 distinct DNA target sequences using fluorescence permutation. Experimentally, we demonstrated a single-tube qPCR assay screening 21 sepsis-related bacterial DNA targets in samples of blood, sputum, pleural effusion and bronchoalveolar lavage fluid, with 89% clinical sensitivity and 100% clinical specificity, showing its potential as a powerful tool for advanced quantitative screening in molecular diagnostics.

## Graphical Abstract

**Graphical Abstract:**
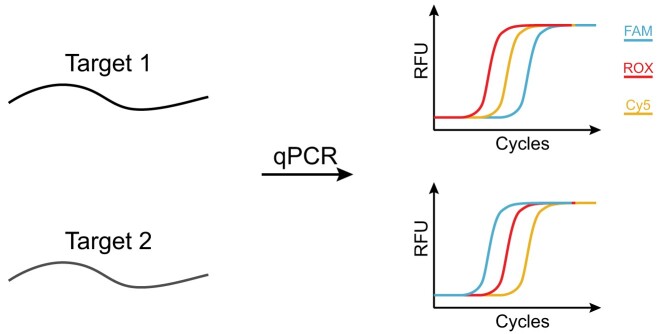

## Introduction

Cost-effective and multiplexed detection of nucleic acids is critical for enabling molecular diagnostic assays for a variety of diseases and, particularly, for syndromic testing (1–4). In syndromic testing cases, a patient presents with nonspecific clinical symptoms potentially caused by multiple pathogens; rapid and cost-effective identification of the specific pathogen(s) infecting a patient can help clinicians determine optimal treatment methods, thereby improving patient outcomes (5,6). Next-generation sequencing (NGS) has gained research interests in pathogen identification due to its accuracy, genomic coverage and falling costs. However, traditional NGS assays involve long and complex library preparation unsuitable for rapid syndromic testing in outpatient setting (7–9). Despite of cost reductions, NGS remains cost prohibitive for non-high throughput applications in diagnostic laboratories (10–12). To address this gap, Oxford Nanopore's automated, multiplexed 16S panel offers pathogen detection from sample to sequences with relatively low cost and fast workflows (13,14). Yet specialized data analysis requirements make it challenging to provide easily interpretable results to clinicians (15). Further optimization for workflow, cost and data analysis is needed to realize the potential of NGS for point-of-care applications.

Moreover, the variety of diagnostic platforms leveraging different technologies has expanded options from sample collection to informing patient care decisions. GenMark's ePlex system (16) uses microarray for blood culture identification (BCID) of multiple pathogens and resistance gene profiling. Additionally, T2 (17,18) Biosystem can directly detect up to 5–6 clinically relevant pathogens from whole blood samples within 5 h.

Highly multiplexed PCR platforms, such as the BioFire FilmArray (Biomérieux) (19,20), have gained adoption for some applications. However, the need for customized consumable cartridges, the lack of pathogen DNA quantitation, and the lower installed instrument base for these instruments compared to qPCR limit their reach and impact on clinical practice (21–23). In contrast, quantitative PCR (qPCR) (24,25) instrument systems are broadly deployed across many clinical laboratories and hospitals across the world (26,27) (Table 1) and provide data that is more readily accessible for clinical reporting.

**Table 1.** Comparison of technologies for detection and identification of DNA sequences

	Target/ Identification coverage	Quantitative readout	Turnaround time	Cost
qPCR	+	+++	+++	+++
NGS	+++	++	+	+
Nanopore Sequencing (13,14,64)	+++	++	++	++
BioFire FilmArray (19,20)	++	+	+++	++
BIOMÉRIEUX VITEK MS (54,55)	++	+	+	++
Color Cycle Multiplex Amplification	++	+++	+++	+++

Standard qPCR instrument platforms and assays have limited multiplexing capabilities. Typically, commercial qPCR instruments are restricted by the number of distinct LED light sources, filter sets and spectrally distinct fluorophores to between four to six fluorescence channels (28). This limitation is exacerbated by most commercial grade diagnostic assays requiring at least one dedicated fluorescence channel for internal control. However, there are dozens of potential pathogens that can result in similar clinical symptoms, and furthermore there is geographic diversity in the DNA sequences of different strains of bacteria and clades of viruses (29). Consequently, highly multiplexed DNA detection is needed to provide maximum actionable information to the clinician to improve patient outcomes (30).

Researchers has devoted their efforts to generate new methods that increased the multiplicity of qPCR. The color mixing strategy (31) employs combinations of fluorescence to enhance detection capabilities, and signal amplitude-based method like Chromacode (32,33) and ddPCR (34,35) utilize the probe concentration and different fluorophores to increase the multiplicity. Qiuying et al utilized the amplicon length and high-resolution melting curve analysis to increase the qPCR multiplicity by 10-fold (36).

**Figure 1.**
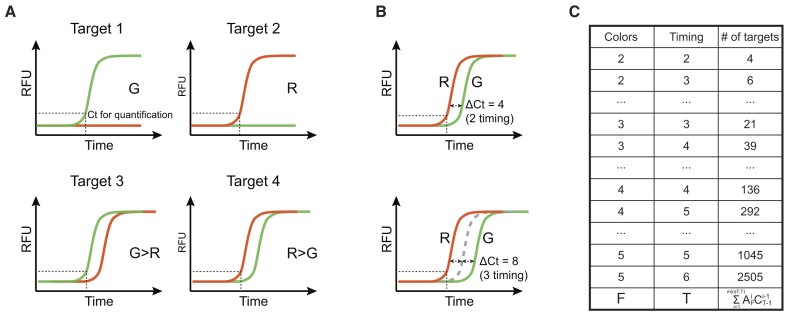
Color cycle multiplex amplification (CCMA) concept and target identification capacity. (**A**) Diagram illustrating the principle supporting CCMA. CCMA introduces a programmable Ct delay (timing) between fluorescent signals to identify targets based on their order of fluorescence appearance. Using two color channels (G, green and R, red), CCMA can distinguish up to four targets. (**B**) Increasing the number of discrete Ct delays that can be programmed (timings) benefits the multiplicity of CCMA. (**C**) The theoretical number of targets discriminated by CCMA is determined by F (the number of available color channels) and T (the number of timings).

Here, we present color cycle multiplex amplification (CCMA), a qPCR approach in which each DNA target elicits a pre-programmed permutation of fluorescence increases across multiple fluorescence channels (Figure 1). The fluorescence permutation is implemented through rationally designed delays in qPCR cycle threshold (Ct) using an oligonucleotide blocker, via the blocker displacement amplification (BDA) mechanism (37,38). CCMA allows dramatically higher multiplexing for single-target-present applications, theoretically allowing identification and quantitation of up to 136 distinct DNA target sequences with 4 fluorescence channels. This color permutation strategy provides another fascinating way to increase the detection capability of routine qPCR without additional process.

**CCMA principles**. In CCMA, DNA targets are identified according to the order of fluorescence appearance (Figure 1A). The number of targets that can be discriminated increases exponentially as a function of the number of color channels used, and also with the number of discrete Ct delays that can be programmed (timings) (Figure 1B, Supplementary Figure S1). We denote the number of fluorescence color channels as F, and the number of distinct timings as T. For example, given F = 3 color channels (R = red, G = green, and B = blue) and T = 2 timings (1 programmable delay), a total of 9 total targets (R, G, B, R > G, R > B, B > G, B > R, G > B and G > R) can be simultaneously measured. This number increases to 136 given F = 4 and T = 4 (Figure 1C). To clarify, when using only fluorescence permutation, the number of timings (T) is set by default to match the number of available color channels (F).

**Figure 2.**
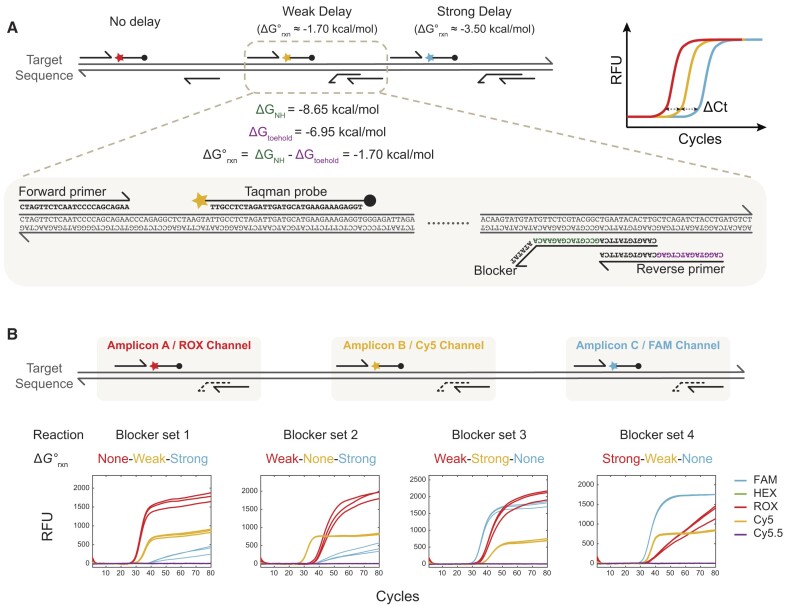
CCMA proof-of-concept results. (**A**) Implementation illustration of 3-color CCMA with 3 timings. For each target, a set of three amplicons were designed, and each amplicon had a TaqMan probe corresponding to a distinct fluorescence channel. To program 2 discrete Ct delays (3 timings), 2 blockers with weak or strong ΔGrxn were designed and allocated to two amplicons, respectively. The stronger the blocker's template binding affinity over that of the reverse primer, the severer inhibition of the amplification efficiency of the amplicon, and a later fluorescence signal is observed. (**B**) Design flexibility of CCMA. Ct delays of each channel can be easily manipulated by alternating blockers sets.

**CCMA implementation using blocker displacement amplification**. The multiplexing power of CCMA depends intimately on the ability to stably control and manipulate Ct. We use rationally designed blockers to modulate the Ct delays for different fluorescence signals, by programmably attenuating the PCR amplification of specific amplicons. The region of the DNA template that the blocker binds to overlaps with that of the reverse PCR primer, resulting in a competitive hybridization reaction (39) (Figure 2A). By strengthening the relative binding thermodynamics of the blockers, or by increasing the stoichiometry of the blocker, the reverse primer will bind less efficiently to the DNA template, resulting in reduced DNA amplification yield. In Figure 2B, for blocker set 1, Amplicon A has no blocker and amplifies maximally efficiently; Amplicon B has a weak blocker and is programmed to amplify with a Ct that is 5 cycles later than Amplicon A. Amplicon C has a strong blocker and is programmed with a Ct value that is 10 cycles later than Amplicon A. The Ct value of Amplicon A depends on the concentration of the DNA template and can still be used for DNA quantitation purposes.

## Materials and methods

### Reagents

TaqPath ProAmp Master Mix (Thermo Fisher Scientific, USA), IDTE (Integrated DNA Technologies, USA), AMPure XP SPRI magnetic beads (Beckman Coulter Life Sciences, USA), Carrier RNA (Roche, Switzerland), NEBNext Ultra II DNA Library Prep Kit for Illumina (New England Biolabs, USA), NEBNext Multiplex Oligos for Illumina (Index Primers Set 1) (New England Biolabs, USA). TaqMan probes, primers, blockers and gBlocks were purchased from IDT technologies and the sequences are listed in Supplementary Table S1–S5.

***Bacterial genomic DNA samples*.**Quantitative bacterial genomic DNA (gDNA) were purchased from American Type Culture Collection (ATCC, Supplementary Table S6). Human gDNA (NA18562) were purchased from Coriell Institute for Medical Research. Bacterial quantitative gDNA (ATCC) and human gDNA (NA18562) were fragmented using M220 focused-ultrasonicator (Covaris, USA) with a targeted length of fragment set at 250bp. Fragmented DNA was quantified by Qubit 4 Fluorometer (Thermo Fisher Scientific, USA) using Qubit dsDNA HS Assay Kit (Thermo Fisher Scientific, USA).

***Clinical samples collection and pathogen DNA extraction*.**The clinical blood samples and blood culture samples from bloodstream infection patients, patients with suspected bloodstream infection and healthy individuals were obtained from Zhongshan Hospital, Fudan University. The sputum samples, bronchoalveolar lavage fluid (BALF) and pleural effusion samples from patients and healthy individuals were obtained from Shanghai Pulmonary Hospital, Tongji University. The pathogen DNA were extracted from clinical samples with Pathogen Lysis Tube L (Qiagen, Germany), QIAamp UCP Pathogen Mini Kit (Qiagen, Germany) and TissueLyser LT L (Qiagen, Germany). The extracted DNA was quantified with Nanodrop (Thermo Fisher Scientific, USA) and Qubit 3.0 Fluorometer (Thermo Fisher Scientific, USA) using a dsDNA HS assay (Q32854).

***Primer specificity validation***. All 21 Bacterial DNA template (gBlocks, input of ∼3000 template copies; refer to sequences in Supplementary Table S5) was mixed with the primers (refer to concentrations in Supplementary Table S7; refer to sequences in Supplementary Table S2), and then underwent 60 cycles of PCR using Taqpath ProAmp polymerase. The reaction volume was 50 μl and the thermocycling protocol is listed in Supplementary Table S8. The amplicon products were then purified by SPRI (AMPure XP beads, ratio of beads solution to products solution = 1.8). Libraries were constructed using standard ligation-based NGS library preparation procedures (Kit: NEBNext Ultra II DNA Library Prep Kit for Illumina and NEBNext Multiplex Oligos for Illumina) and then sequenced by MiniSeq/ NextSeq sequencer (Illumina, USA).

***Single-* *plex* *CCMA tests***. A typical real-time PCR reaction consisted of a mixture of TaqPath ProAmp Master Mix, bacterial DNA template (gBlocks, input of ∼3000 template copies; refer to sequences in Supplementary Table S5), H_2_O, human gDNA (0.7 ng/μl, 200 copies/μl), primers pair (50 nM each, refer to sequences in Supplementary Table S2), TaqMan probe (200 nM, refer to sequences in Supplementary Table S3) and blocker (500 nM, refer to sequences in Supplementary Table S4). The total volume of each reaction was 50 μl. PCR was performed using a Bio-Rad CFX96 Touch Real-Time PCR Detection System (Bio-Rad, USA) and the detailed procedures were listed in Supplementary Table S8. For blocker selection, eight different blocker combinations were tested for each single-plex test.

***Multiplexed CCMA tests***. A typical real-time multiplexed PCR reaction consisted of a multiplexed mixture of TaqPath ProAmp Master Mix, bacterial DNA template (gBlocks, input of ∼300 template copies; refer to sequences in Supplementary Table S5), H_2_O, human gDNA (0.7 ng/μl, 200 copies/μl), primers, TaqMan probes and blockers (refer to sequences and concentrations in Supplementary Table S2–S4 and Supplementary Table S7). The total volume of each reaction was 50 μl. Data analysis process in shown in Supplementary Section 4 and Supplementary Figure S10.

For panel development and experiments optimization, see details in Supplementary Section 5. For PCR thermocycling procedure optimization, different protocols (listed in Supplementary Table S8) were tested. gBlocks of *Staphylococcus epidermidis* with input amount at 300 copies were used as the DNA template. For host DNA interference tests, gBlocks of *Staphylococcus epidermidis* (300 copies) was mixed with human gDNA (10 000 copies and 100 000 copies), cattle DNA (100 000 copies) and chicken DNA (100 000 copies), respectively. For clinical sample background interference tests, mimic samples representing a variety of sample types were created by mixing gBlocks of *Staphylococcus epidermidis* (300 copies) with 200 μl whole blood, 50 μl PBMC, 100 μl plasma, 500 μl sputum, 100 μl cheek swab and dry blood spot with diameter at 6 mm, respectively. Then, DNA from mimic samples were extracted using QIAamp UCP Pathogen Mini Kit (Qiagen, Germany) and followed by CCMA tests. For LoD assessment, gBlocks of different species with input of ranging from 5 to 1800 copies were used as the DNA template.

For proof-of-concept experiment of modular development of higher-plex CCMA panels, the primers, blockers and probes of 63-amplicon sepsis-related pathogen detection panel were mixed with 168-amplicon Pan-Cancer detection panel (40) under different primer ratio (Supplementary Table S9–S10), and then followed the same multiplex CCMA test procedure.

***Bacterial genomic DNA sample and clinical sample test***. In a typical multiplex CCMA assay with a total volume of 50 μl, bacterial quantitative gDNA sample (purchased from ATCC, inputs of 5–1000 template copies; refer to sample list in Supplementary Table 6S) or clinical sample DNA (extracted from clinical samples, inputs of 6 ng to 1 μg, see details in Supplementary Table S11) were used as the DNA template. The PCR procedure used was the same as the multiplexed CCMA tests.

***Cross-validation***. All clinical samples (blood, blood culture, sputum, sputum culture, BALF and pleural effusion) also went through the standard automatic bacterial culture process using Biomérieux BacT/ALERT 3D. Pathogens in cultured samples were identified by smear microscopy and VITEK Mass Spectrometry Microbial identification system (Biomérieux VITEK spectrometry microbial identification system).

## Results

### Proof-of-concept

To demonstrate 3-color CCMA with three timings, we first designed three primer sets targeting the *Chlamydia pneumoniae*-genome using TaqMan probes labeled with ROX, Cy5 or FAM (Figure 2). For each amplicon, we designed two different blockers to provide different Ct delay options. For any given experiment, at least one primer set would stay unblocked and remaining primer sets would be coupled with one blocker each to generate the desired fluorescence order.

To prove that CCMA can potentially accommodate higher multiplicity, we tested a 4-color CCMA with four timing system targeting aac(3)-II, a gene found in many pathogenic bacteria (41) (Supplementary Figure S2). Four fluorescence channels and three blockers (Supplementary Figure S3) were used to create sequential Ct delays of 6, 4 and 2 cycles. Additional manipulation of Ct delays can be achieved by adjusting the reaction stoichiometry (Supplementary Figure S4).

### 21 species pathogen detection panel

**Figure 3.**
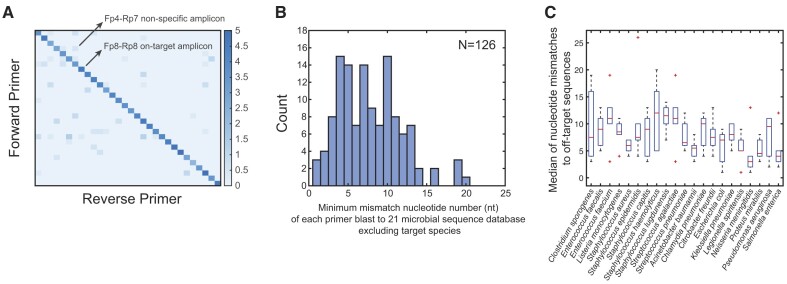
Sequence specificity of multiplexed target detection. (**A**) Primer specificity analysis using next-generation sequencing (NGS). Multiplexed PCR were performed using primers and synthetic double-stranded DNA templates (gBlocks) of all 21 bacterial species with human gDNA background. The multiplex PCR products were then purified, ligated, indexed and sequenced. Sequencing reads were analyzed and categorized into on-target amplicons (diagonal) and non-specific amplicons. The reads of corresponding primer pairs are shown in log scale in the heatmap. Full heatmap is given in Supplementary Figure S7. (B, C) Genomic specificity of primers. (**B**) Histogram of the minimum number of mismatched nucleotides of each primer when BLASTed against the whole genome sequences of all 21 species (Supplementary Figure S8). (**C**) Projection of genome specificity of six primers into each species.

**Design specificity**. Next, the utility of CCMA is demonstrated in a massively multiplexed qPCR panel designed to detect DNA from 21 sepsis-related pathogen. Considering the high redundancy of sequences shared between microbiome species, a pipeline (Supplementary Figure S5) is built to down-select signature regions that can uniquely represent each species. Primer candidates are automatically designed against these unique regions with minimized dimerization using our previously published SADDLE algorithm (42). Length distribution of the designed amplicons is shown in Supplementary Figure S6. Synthetic double-stranded bacterial DNA templates (gBlocks) of 21 species were first mixed with high concentration of human genomic DNA (gDNA), and then amplified using the designed primers. PCR product was subjected to NGS and the results confirmed high reads dominancy of desired amplicons over off-target reads (Figure 3A and Supplementary Figures S7 and S8). Genomic specificity of primers was validated by analyzing the minimum mismatch nucleotide number of each primer blast to 21 microbial sequence database excluding target species. As shown in Figure 3B and Supplementary Figure S9, mismatches between each primer sequence to the corresponding most possible off-target binding site ranges from 1 to 20 nt with a median of 7 nt. Moreover, a similar strategy to analyze the specificity of six primers corresponding to each target species in the panel was performed, and the results are plotted in Figure 3C.

Blockers and TaqMan probes were computationally designed for each bacterial species to result in a unique fluorescence order (Supplementary Tables S2–S5). For each species, blockers with the best timing separation were selected from blocker candidates with different binding affinities by screening 8 blocker combinations (Supplementary Figures S11 and S12). Single-plex CCMA results for each species are shown in Supplementary Figure S13.

**Figure 4.**
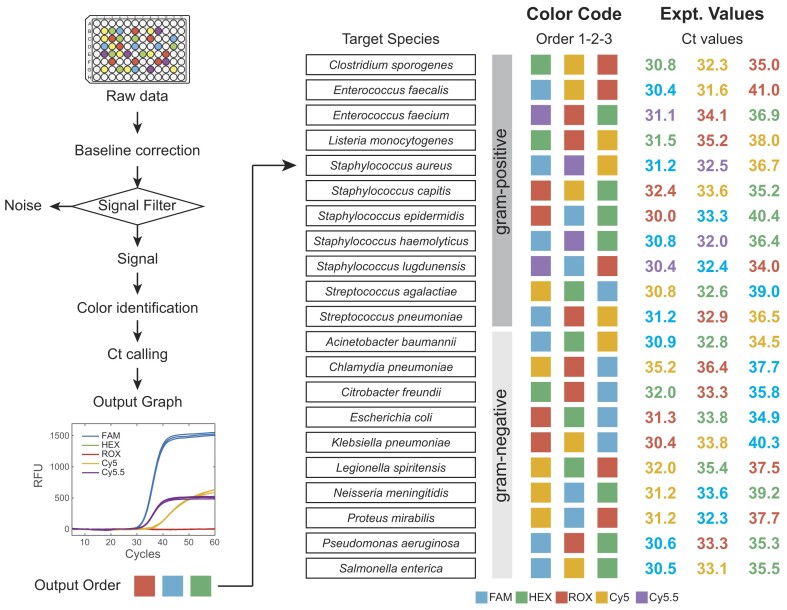
Overview of sepsis-related bacteria detection panel based on CCMA. A PCR reaction was conducted with a mixture of PCR primers, amplicon-specific TaqMan probes, blockers of the selected 63 amplicons, gBlocks of the target species and excess human gDNA background. Raw fluorescence signals in all channels were collected and filtered into true signal vs noise. Each true signal then underwent baseline correction, color identification (Supplementary Figure S9), resulting in PCR amplification curves with a specific order of fluorescence signal appearance and Ct calls unique to each bacterial species. Average Ct values across three parallel experiments (100 copies gBlock input) are shown (see details in Supplementary Excel 1). The identity of the pathogen was then determined by cross-referencing the experimental order against the color code indices.

**Panel development in multiplex**. Multiplexed qPCR reactions were then performed for each of the 21 contrived samples containing human gDNA as background with all primers, selected blockers, and TaqMan probes combined. Figure 4 shows resulting fluorescence orders. Targets were identified by their expected order of signal emergence for a unique combination of three fluorescence curves. Furthermore, quantitative information about target concentrations could be acquired from the first Ct value. Our assay detected sequences from 21 bacterial species using only 5 fluorescence channels and 3 timings. Detailed qPCR curves and quantitation standard curves for 21 species are given in Supplementary Figures S14 and S15.

In developing the 21-species pathogen detection panel from single-plex CCMA tests, oligonucleotide concentrations and thermocycling conditions were optimized to enable multiplex PCR reaction. Compared to single-plex PCR, the multiplex system contains more primer and probe sequences, necessitating decreased concentrations of each oligonucleotide to accommodate the increased sequence quantity within the overall reaction. Details on optimized concentrations are provided in Supplementary Table S7.

**Figure 5.**
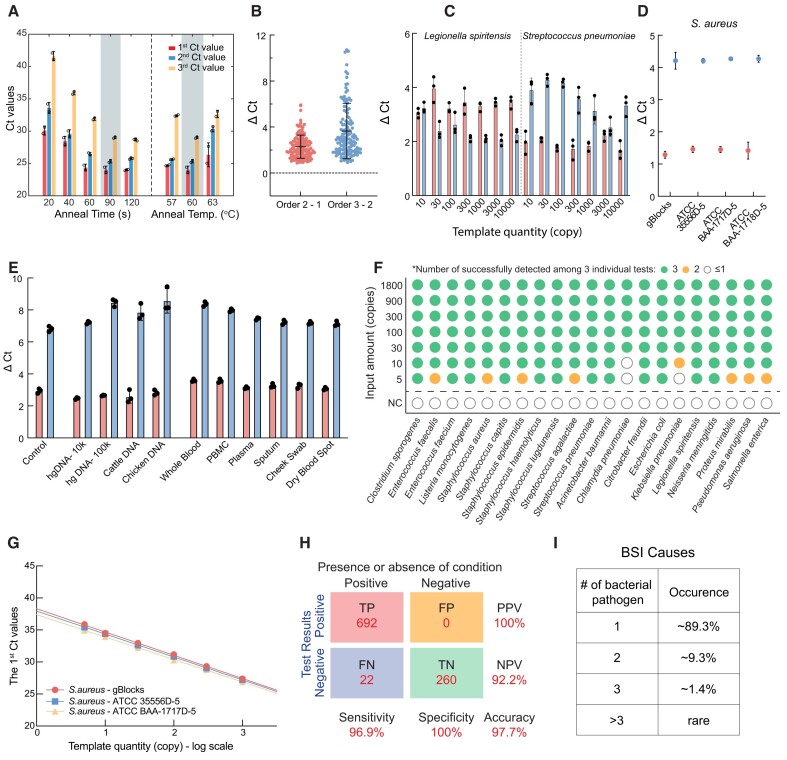
Performance of sepsis-related bacteria detection panel based on CCMA. (**A**) Ct values of Staphylococcus epidermidis with input amounts of 5000 copies using CCMA with different PCR annealing times and annealing temperatures. Optimal condition used in following figures is emphasized in shallow grey. (**B**) Sequential Ct delay. Violin plots show ΔCt between the first 2 fluorescent signals (Order 2–Order 1, shown in maple) and between the last 2 fluorescent signals (Order 3–Order 2, shown in navy). *n* = 3 biological replicates for 21 species, SD = 1.0 and 2.4, respectively. The color code used for the ΔCt values also applies to (C) and (D). (**C**) Relationship between ΔCt values and input amount of template (gBlocks of Legionella spiritensis and Streptococcus pneumoniae). (**D**) Ct delay concordance for Staphylococcus aureus using different PCR template types (gBlocks and extracted DNA from three cell lines). (**E**) Robustness testing. Comparison of ΔCt values for Staphylococcus epidermidis gBlocks with different host DNA interferences (human genomic DNA, cattle DNA, chicken DNA) or background interferences (human whole blood, PBMC, plasma, sputum, cheek swab, dry blood spot). (**F**) Limits of detection (LoD) for all 21 bacterial species. For each species, synthetic bacterial DNA was mixed with human gDNA to desired copy numbers and tested in triplicate. Missing or extra colors, incorrect order, or failed separations were considered detection failures. Colors represent number of successful measurements across three tests. Negative control (NC) represents human gDNA background only. Individual species data in Supplementary Figure S13. (**G**) Quantitative copy number detection using the first Ct value of CCMA. Linear regression of the logarithm of template quantities (3 Staphylococcus aureus genomes from ATCC) and the first Ct value. Complete results for all species in Supplementary Figure S14. (**H**) Statistical analysis of CCMA tests for bacterial genome samples. TP = true positive, FP = false positive, FN = false negative, TN = true negative. (**I**) Occurrence of bloodstream infections caused by monomicrobial and polymicrobial pathogens (43–51).

However, primer-template binding kinetics became rate-limiting at the lowered concentrations. Extended annealing time during each thermocycling cycle was required for complete primer-template hybridization across all 63 sets of primers. Thermocycling parameters for the 21-species panel were systematically examined and results are summarized in Figure 5A. Using 300 copies of *Staphylococcus epidermidis* as template and annealing temperature as 60°C, extended annealing time from 20s to 90s incrementally lowered Ct values across all color channels, allowing sufficient kinetics for oligo binding even at low target inputs. No further Ct improvements occurred beyond 90s, indicating binding equilibrium was reached. Fixing annealing time at 90s, the optimal temperature was likewise assessed. Figure 5A demonstrates 60°C yielded maximal and most stable Ct differences between ordered neighboring fluorescent signals, providing optimal color order and timing determination.

**Panel robustness**. The robustness of CCMA was tested through different approaches. For CCMA tests of 21 bacterial species with different input amount ranging from 5 to 1800 copies, the ΔCt values of between neighboring signals are plotted in Figure 5B and Supplementary Figure S19. CCMA achieved significant and stable timing discrimination with varied template quantity, resulting in median Ct differences of 2.30 and 3.65 cycles between the neighboring signals, respectively. A consistent maintenance of cycle threshold (Ct) differences above 0.9 demonstrates the robustness of the CCMA color coding system. Figure 5C shows Ct differences fluctuations with different template quantity of *Legionella spiritensis* and *Streptococcus pneumoniae*. While some variability is seen for species *Staphylococcus haemolyticus* and *Citrobacter freundii*, other species maintain delays across different template quantity (Supplementary Figures S16 and S17). From the perspective of retaining correct color orders, CCMA is generally robust across concentrations, with refinements possible for limited species to further stabilize quantified delays.

Natural variation between strains within a bacterial species was examined for assay robustness. Figure 5D demonstrates highly consistent ΔCt values across three unique *Staphylococcus aureus* strains relative to the gBlocks control template. This verifies the reliability of the CCMA pipeline for universal identification of a targeted species regardless of strain variation.

The clinical resilience of CCMA assay was interrogated through challenging sample conditions. As shown in Figure 5E, CCMA detection of 300 copies of *Staphylococcus epidermidis* under excess host DNA quantities, including human (10^4^ and 10^5^ copies), cattle (10^5^ copies) and chicken (10^5^ copies), produced minimal Ct divergence versus gBlock-only controls. Furthermore, a low *Staphylococcus epidermidis* input (300 copies) was spiked into complex matrices like whole blood, PBMCs, plasma, sputum, buccal swabs, and dried blood spots for DNA extraction and followed by CCMA tests. Across the sample types, ΔCt values between 3 fluorescent remained stable at approximately 3 and 7 cycles, respectively. Additional robustness analysis based on plateau fluorescence signal intensity is shown in Supplementary Figure S20.

Together, these results strongly supported CCMA assay's robustness against potential interfering host DNA and background matrix components, enabling reliable identification even with low pathogen inputs across diverse clinical sampling procedures.

**Detection sensitivity and specificity**. Analytical limit-of-detections (LoD) were assessed individually for all 21 species with different input template quantities (5, 10, 30, 100, 300, 900 and 1800 copies). As shown in Figure 5F, and Supplementary Figure S14, CCMA achieved successful detection of 30 copies for all species. For 12 out of 21 species such as *Listeria Monocytogenes*, LoD as low as 5 copies was achieved.

**DNA target quantitation accuracy and reproducibility**. CCMA allows accurate quantitation of DNA targets, a feature unavailable to many multiplex qPCR assays. The first Ct value is not artificially delayed by any blockers, and, thus, can be used to infer the DNA target concentration using a standard calibration curve. Furthermore, in the detection of 21 species with an input of 100 copies each, the standard deviation across three replicates was calculated as detailed in Supplementary Excel 1. The median standard deviations for the 1st, 2nd and 3rd Ct values were 0.18, 0.25 and 0.31, respectively. Regression analysis showed the calibration curve coefficients of determination to range from 0.994 to 0.999 (Figure 5G, Supplementary Figure S15).

The quantitation reproducibility of CCMA was validated through repetitive testing across differing production batches, operators, and timepoints. A tight distribution was observed in the first Ct values (0.25 cycles standard deviation), corresponding to less than 20% input variation (Supplementary Figures S18).

**Genomic DNA validation and quantification**. The performance of CCMA using gDNA derived from bacterial species was examined to better recapitulate clinical testing settings. gDNA yielded similar LoDs to those determined when using gBlocks in 13 different strains of 8 bacterial species (Figure 5F, Supplementary Figure S14). The qPCR curves of the CCMA reactions remained largely consistent across strains from the same species (Supplementary Figure S21). CCMA successfully achieved detection of 7 out of 8 species with template quantity at 5 copies, and 8 out of 8 species with template quantity at 10 copies (Supplementary Figure S22). Ct values for unblocked amplicons showed high concordance with input quantities of bacterial gDNA across strains and concentrations (Figure 5F, Supplementary Figure S23). Statistical analysis of CCMA tests of bacteria genome samples yielded a sensitivity of 96.9% and a specificity of 100% (Figure 5H). No significant correlation was observed between off-target amplicons and LoD (Supplementary Figure S24).

**Overcoming the single-target-present limitation**. CCMA relies on more than one color to identify a single target. When multiple targets present in a s single sample, interference between same color from different target species will significantly affect species identification and quantification results. CCMA with 2-tube strategy can over overcome the single-target-present limitation. Successful determination of the existence of two simultaneous species amongst a total of 20 species can be achieved using CCMA with a modified color code for 2-tube detection using the same five fluorescence channels (Supplementary Figure S25). Preliminary proof-of-principle data for this concept is shown in Supplementary Figure S26. In tube one, targets α, β and γ are encoded by Blue > Red, Red > Green and Blue > Green, respectively. The co-existence of any two of these three targets (αβ, βγ or αγ) will all lead to the fluorescence readout order Blue > Red > Green, but each distinct target combination will be identified by distinct color codes. Thus, using this approach, we can detect the presence of any individual or pair of the 20 species within a sample.

In cases of bloodstream infection (BSI), approximately 89.3% are attributed to infections by a single bacterial species (Figure 5I) (43–51). In instances of polymicrobial bloodstream infections (PBSI), the predominant scenario involves simultaneous infection by two distinct pathogens (around 78–96% of PBSI cases, approximately 9.3% of all BSI cases), while three pathogens are found in a smaller proportion of cases (around 4–22% of PBSI cases, approximately 1.4% of all BSI cases). Infections with more than 3 pathogens are relatively rare (45,50,51). CCMA coupled with two-tube strategy enables the expedited identification of BSI cases caused by either one or two pathogens. This approach is applicable to about 98.6% of clinical BSI cases.

**Figure 6.**
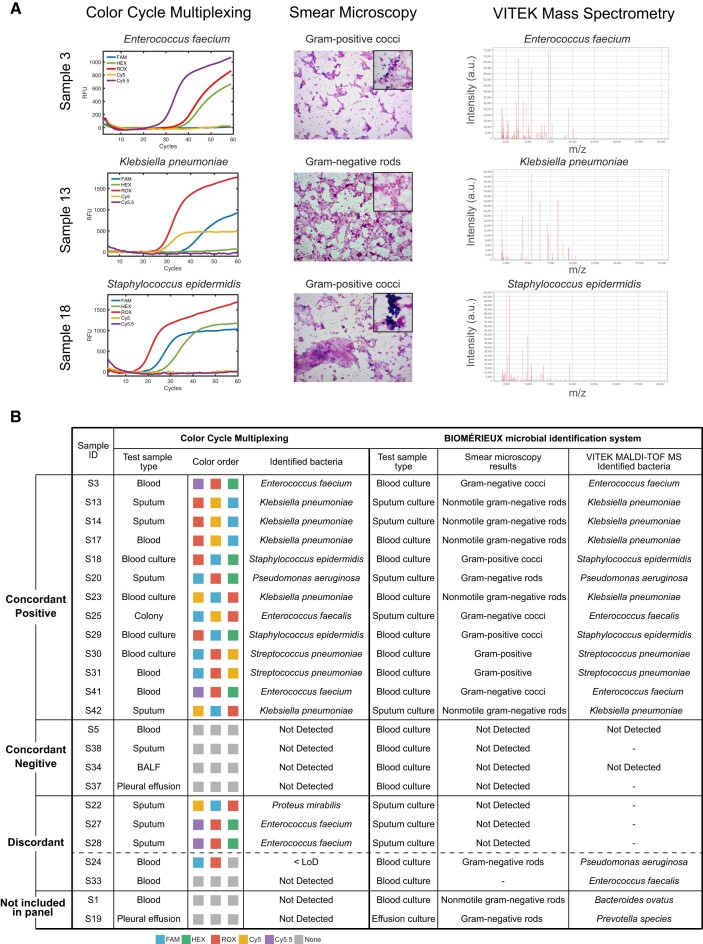
Comparison of CCMA and Biomérieux microbial identification system for the detection of pathogen in clinical samples. (**A**) The pathogen existence in the three clinical samples (S3-blood, S13-sputum and S18-blood culture) were identified by CCMA, and Biomérieux microbial identification system respectively. Left panel: CCMA fluorescence curve and corresponding identified pathogen results; middle: smear microscopy photos of cultured samples with magnification of 10 × 40 (magnification of zoom-in: 10 × 100); right: MALDI-TOF mass spectrum and corresponding identified pathogen results using Biomérieux VITEK MS. (**B**) Summary of cross-validation results. CCMA applied to 45 clinical samples (18 blood, 10 blood culture, 10 sputum, 3 pleural effusion, 3 bronchoalveolar lavage fluid (BALF) and 1 colony). The infectious pathogen existence in the sample is identified by the color order. The pathogen existence in samples were also identified by Biomérieux microbial identification system including standard automatic bacteria culture, smear microscopy identification and VITEK MALDI-TOF MS microbial identification. The results of the cross-validation were divided into four categories: (i) concordant positive, (ii) concordant negative, (iii) discordant and (iv) not included in panel.

**Application of CCMA to detect low concentration pathogen in clinical samples**. The sepsis-related bacteria detection panel based on CCMA has been applied to 45 clinical samples and totally 16 positive samples were identified (Figure 6 and Supplementary Figures S27). All 45 clinical samples were further validated by ‘gold-standard’ Biomérieux microbial identification system (52,53). After 10–18 h of bacteria incubation, the existence and morphology of the bacteria were identified by smear microscopy. The bacteria type of positive samples was further identified by VITEK MALDI-TOF MS (54,55). Figure 6A shows the test results of three clinical samples (S3-blood, S13-sputum and S18-blood culture) by CCMA as well as the smear microscopy and mass spectrum of the corresponding cultured samples. The test results of CCMA show good concordance with the ‘gold-standard’ methods. S3, S13 and S18 were identified as positive for *Enterococcus faecium*, *Klebsiella pneumoniae* and *Staphylococcus epidermidis*, respectively. Figure 6B and Supplementary Figure S28 shows the comparison summary of CCMA and Biomérieux microbial identification system for the detection of bacteria in clinical samples. Overall, the identification results of two methods showed a fairly good concordance (98.8%) regardless of the sample types variations. Moreover, bacteria in three sputum samples (S22, S27 and S28) could be easily identified by CCMA with first Ct values at 29.4, 30.7 and 34.1, respectively, indicating that the bacteria load of the sample present at low level. However, the pathogen existence of the 3 samples cannot be identified by BIOMÉRIEUX system. Taken together, across of a total of 45 unknown clinical samples (Figure 6B and Supplementary Figure S27), we observed an 88.9% (16/18) clinical sensitivity and a 100% (27/27) clinical specificity. Two false negative results were observed for samples with low bacteria load.

## Discussion

In this report, we introduce the CCMA assay design approach. CCMA allows simultaneous screening of many different DNA targets in a single tube reaction using conventional qPCR instrumentation. Experimentally, we demonstrated a CCMA assay using 4 fluorescence channels targeting 21 sepsis-related pathogenic bacterial DNA, as these bacteria remain a significant cause of global morbidity and mortality (56,57). Previously characterized techniques have leveraged melting temperature analysis, amplitude modulation of TaqMan probes and ligation fluorescence coding of primers (58,59). However, these techniques have not achieved the 21 actual DNA targets or the 136 possible targets given 4 colors and 4 timings identified by CCMA. Other attempts to increase the utility of qPCR have leveraged novel pooling strategies (60).

The programmed timing delay of the Ct values for distinct fluorophores offers a powerful mechanism for increasing the plexity of CCMA. For example, with two fluorescence channels and three programmable Ct timings, we can achieve 6-plex rather than 4-plex based on two programmable Ct timing. In principle, BDA optimization can generate reproducible, discrete delays <3 cycles and the increased number of timings can vastly increase the plexity beyond the two timings shown here.

Modular development can be a potential approach to further increase the multiplicity of CCMA with limited resource consumption. Developing a 136-species assay for instance, we can potentially optimize reactions by splitting into modules (i.e. ∼20 species per module in one tube, with 7 total modules) before mixing for final unified fine-tuning.

To demonstrate that Ct delays can be preserved when consolidating modules, we tested the 21-species CCMA panel in the presence of an additional 168-amplicon Pan-Cancer PCR assay targeting human genomic DNA (40) under different primer ratio between two assays. This reference assay was derived from SADDLE design algorithm and optimized for target specificity and primer dimer internally. Considering obvious difference in genome sequences between CCMA and Pan-Cancer assays, there is no extra primer screening and primer dimer analysis conducted before experiments. As shown in Supplementary Tables S9 and S10, for the detection of *Staphylococcus epidermidis*, the Ct values for all three color channels remained highly consistent with or without the presence of 168-plex assay primers, indicating the primer/blocker system is working as expected without interference from background primers targeting other potential species. In addition, the clean detection of target species confirmed the absence of non-specific amplification and false positive probe signals. These results provide evidence that a modular development workflow is viable for expansive CCMA with computational specificity screening of primer sequences. Rational primer design algorithm limits risks of primer cross-reactivity upon assay merging by optimizing primer interaction orthogonality in advance to experiments.

Despite that SADDLE algorithm successfully generated primers for the 21-species panel shown in Figures 4 and 5 and offered potential in modular CCMA assay development, the variation in the first Ct values for the different DNA targets suggest that these primers have different amplification efficiencies. The variations in first Ct values indicates the multiplexed design of the primer sequences for many targets potentially limits the design of higher-plex CCMA assays. We expect the variability in Ct values for distinct primers to increase at higher plexity; achieving 100-plex or higher single-tube qPCR assays will likely require additional research in multiplex primer design algorithms that result in more uniform PCR amplification.

We successfully applied CCMA 21-species pathogen detection panel to clinical samples and the test results were cross-validated by ‘gold-standard’ Biomérieux microbial identification system. Overall, the identification results of two methods showed a fairly good concordance (85%) regardless of the sample types variations. Compared to the bacteria incubation and detection methods, CCMA could identify the bacteria existence in clinical sample without bacteria incubation, which could reduce the turnaround time from days to hours. Moreover, the clinical sample volume requirements for CCMA assays are substantially reduced versus traditional methods. The ‘gold-standard’ Biomérieux microbial identification system requires three tubes of 8–12 ml whole blood for three bottles of blood culture. By comparison, CCMA provides rapid quantitative screening using <200 μl of whole blood. This significant reduction in sample volume relieves the companion diagnostic burden.

We envision CCMA assuming the role of NGS in mid-plex scenarios that require a large panel of targets, such as syndromic testing and companion diagnostic testing in cancer. Currently available cancer companion diagnostic tests have turnaround times of 4–14 days (61,62). Turnaround time for CCMA is expected to be similar to that of routine qPCR assays, potentially within the time frame of hours. Furthermore, currently FDA-cleared adjunct tests, including the Biofire FilmArray, use multiplex PCR to detect multiple targets. However, these tests can only confirm the presence of DNA targets. CCMA can not only detect, but also quantify DNA targets and, therefore, can provide insight into bacterial load in septic intensive care patients.

As hypothesis-free NGS (63) is being increasingly applied to many areas of medicine, our knowledge of the DNA markers that contribute to disease is being becoming more complete. For routine deployment of diagnostics to known DNA markers, qPCR offers significant advantages in time, cost and ease-of-use. Here, CCMA offers another fascinating way to advance the multiplicity of the routine qPCR using color permutation. CCMA and other approaches that can empower qPCR can accelerate the onset of a world with frequent and affordable molecular diagnostic testing, improving patient outcomes through rapid and accurate guidance of optimal treatment.

## Supplementary Material

gkae683_Supplemental_Files

## Data Availability

The data underlying this article are available in the article and in its online supplementary material. Source data and code are available upon request.

## References

[1] Forshew T. , Murtaza M., Parkinson C., Gale D., Tsui D.W., Kaper F., Dawson S.J., Piskorz A.M., Jimenez-Linan M., Bentley D. et al. Noninvasive identification and monitoring of cancer mutations by targeted deep sequencing of plasma DNA. Sci. Transl. Med. 2012; 4:136–168. 10.1126/scitranslmed.3003726 22649089

[2] Monard C. , Pehlivan J., Auger G., Alviset S., Tran Dinh A., Duquaire P., Gastli N., d’Humieres C., Maamar A., Boibieux A. et al. Multicenter evaluation of a syndromic rapid multiplex PCR test for early adaptation of antimicrobial therapy in adult patients with pneumonia. Crit. Care. 2020; 24:434. 32665030 10.1186/s13054-020-03114-y PMC7359443

[3] Cassidy H. , Van Genne M., Lizarazo-Forero E., Gard L., Niesters H.G.M. A discussion of syndromic molecular testing for clinical care. J. Antimicrob. Chemother. 2021; 76:iii58–iii66. 34555161 10.1093/jac/dkab243 PMC8460109

[4] Gray J. , Coupland L. The increasing application of multiplex nucleic acid detection tests to the diagnosis of syndromic infections. Epidemiol. Infect. 2014; 142:1–11. 24093495 10.1017/S0950268813002367 PMC9152551

[5] Dumkow L.E. , Worden L.J., Rao S.N. Syndromic diagnostic testing: a new way to approach patient care in the treatment of infectious diseases. J. Antimicrob. Chemother. 2021; 76:iii4–iii11. 34555157 10.1093/jac/dkab245 PMC8460095

[6] Ramanan P. , Bryson A.L., Binnicker M.J., Pritt B.S., Patel R. Syndromic panel-based testing in clinical microbiology. Clin. Microbiol. Rev. 2018; 31:e00024-17. 29142077 10.1128/CMR.00024-17 PMC5740973

[7] Mutalik V.K. , Novichkov P.S., Price M.N., Owens T.K., Callaghan M., Carim S., Deutschbauer A.M., Arkin A.P. Dual-barcoded shotgun expression library sequencing for high-throughput characterization of functional traits in bacteria. Nat. Commun. 2019; 10:308. 30659179 10.1038/s41467-018-08177-8 PMC6338753

[8] Kim S. , De Jonghe J., Kulesa A.B., Feldman D., Vatanen T., Bhattacharyya R.P., Berdy B., Gomez J., Nolan J., Epstein S. et al. High-throughput automated microfluidic sample preparation for accurate microbial genomics. Nat. Commun. 2017; 8:13919. 28128213 10.1038/ncomms13919 PMC5290157

[9] Hu T. , Chitnis N., Monos D., Dinh A. Next-generation sequencing technologies: an overview. Hum. Immunol. 2021; 82:801–811. 33745759 10.1016/j.humimm.2021.02.012

[10] Schwarze K. , Buchanan J., Taylor J.C., Wordsworth S. Are whole-exome and whole-genome sequencing approaches cost-effective? A systematic review of the literature. Genet. Med. 2018; 20:1122–1130. 29446766 10.1038/gim.2017.247

[11] Schwarze K. , Buchanan J., Fermont J.M., Dreau H., Tilley M.W., Taylor J.M., Antoniou P., Knight S.J.L., Camps C., Pentony M.M. et al. The complete costs of genome sequencing: a microcosting study in cancer and rare diseases from a single center in the United Kingdom. Genet. Med. 2020; 22:85–94. 31358947 10.1038/s41436-019-0618-7 PMC6944636

[12] McCombie W.R. , McPherson J.D. Future promises and concerns of ubiquitous next-generation sequencing. Cold Spring Harb. Perspect. Med. 2019; 9:a025783. 30478095 10.1101/cshperspect.a025783 PMC6719590

[13] Greninger A.L. , Naccache S.N., Federman S., Yu G., Mbala P., Bres V., Stryke D., Bouquet J., Somasekar S., Linnen J.M. Rapid metagenomic identification of viral pathogens in clinical samples by real-time nanopore sequencing analysis. Genome Medicine. 2015; 7:99. 26416663 10.1186/s13073-015-0220-9 PMC4587849

[14] Cheng H. , Sun Y., Yang Q., Deng M., Yu Z., Zhu G., Qu J., Liu L., Yang L., Xia Y. A rapid bacterial pathogen and antimicrobial resistance diagnosis workflow using Oxford nanopore adaptive sequencing method. Briefings Bioinf. 2022; 23:bbac453. 10.1093/bib/bbac453 36259361

[15] Curry K.D. , Wang Q., Nute M.G., Tyshaieva A., Reeves E., Soriano S., Wu Q., Graeber E., Finzer P., Mendling W. Emu: species-level microbial community profiling of full-length 16S rRNA Oxford Nanopore sequencing data. Nat. Methods. 2022; 19:845–853. 35773532 10.1038/s41592-022-01520-4 PMC9939874

[16] Carroll K.C. , Reid J.L., Thornberg A., Whitfield N.N., Trainor D., Lewis S., Wakefield T., Davis T.E., Church K.G., Samuel L. Clinical performance of the novel GenMark Dx ePlex blood culture ID Gram-positive panel. J. Clin. Microbiol. 2020; 58:e01730-19. 31996444 10.1128/JCM.01730-19 PMC7098771

[17] Neely L.A. , Audeh M., Phung N.A., Min M., Suchocki A., Plourde D., Blanco M., Demas V., Skewis L.R., Anagnostou T. T2 magnetic resonance enables nanoparticle-mediated rapid detection of candidemia in whole blood. Sci. Transl. Med. 2013; 5:182ra154. 10.1126/scitranslmed.3005377 23616121

[18] Lucignano B. , Cento V., Agosta M., Ambrogi F., Albitar-Nehme S., Mancinelli L., Mattana G., Onori M., Galaverna F., Di Chiara L. Effective rapid diagnosis of bacterial and fungal bloodstream infections by T2 magnetic resonance technology in the pediatric population. J. Clin. Microbiol. 2022; 60:e00292-22. 36069557 10.1128/jcm.00292-22 PMC9580347

[19] Berinson B. , Both A., Berneking L., Christner M., Lütgehetmann M., Aepfelbacher M., Rohde H. Usefulness of BioFire FilmArray BCID2 for blood culture processing in clinical practice. J. Clin. Microbiol. 2021; 59:e00543-21. 33980648 10.1128/JCM.00543-21 PMC8373244

[20] Trujillo-Gomez J. , Tsokani S., Arango-Ferreira C., Atehortua-Munoz S., Jimenez-Villegas M.J., Serrano-Tabares C., Veroniki A.-A., Florez I.D. Biofire FilmArray Meningitis/Encephalitis panel for the aetiological diagnosis of central nervous system infections: a systematic review and diagnostic test accuracy meta-analysis. E. Clinical Medicine. 2022; 44:101275. 10.1016/j.eclinm.2022.101275 PMC8851290 35198914

[21] Metzker M.L. Sequencing technologies - the next generation. Nat. Rev. Genet. 2010; 11:31–46. 19997069 10.1038/nrg2626

[22] Hess J.F. , Kohl T.A., Kotrova M., Ronsch K., Paprotka T., Mohr V., Hutzenlaub T., Bruggemann M., Zengerle R., Niemann S. et al. Library preparation for next generation sequencing: a review of automation strategies. Biotechnol. Adv. 2020; 41:107537. 32199980 10.1016/j.biotechadv.2020.107537

[23] Duff S. , Hasbun R., Ginocchio C.C., Balada-Llasat J.M., Zimmer L., Bozzette S.A. Economic analysis of rapid multiplex polymerase chain reaction testing for meningitis/encephalitis in pediatric patients. Future Microbiol. 2018; 13:617–629. 29316801 10.2217/fmb-2017-0238

[24] Arya M. , Shergill I.S., Williamson M., Gommersall L., Arya N., Patel H.R. Basic principles of real-time quantitative PCR. Expert Rev. Mol. Diagn. 2005; 5:209–219. 15833050 10.1586/14737159.5.2.209

[25] Heid C.A. , Stevens J., Livak K.J., Williams P.M. Real time quantitative PCR. Genome Res. 1996; 6:986–994. 8908518 10.1101/gr.6.10.986

[26] Taylor S.C. , Nadeau K., Abbasi M., Lachance C., Nguyen M., Fenrich J. The ultimate qPCR experiment: producing publication quality, reproducible data the first time. Trends Biotechnol. 2019; 37:761–774. 30654913 10.1016/j.tibtech.2018.12.002

[27] Bustin S.A. , Benes V., Garson J.A., Hellemans J., Huggett J., Kubista M., Mueller R., Nolan T., Pfaffl M.W., Shipley G.L. et al. The MIQE guidelines: minimum information for publication of quantitative real-time PCR experiments. Clin. Chem. 2009; 55:611–622. 19246619 10.1373/clinchem.2008.112797

[28] Huang Q. , Zheng L., Zhu Y., Zhang J., Wen H., Huang J., Niu J., Zhao X., Li Q. Multicolor combinatorial probe coding for real-time PCR. PLoS One. 2011; 6:e16033. 21264249 10.1371/journal.pone.0016033 PMC3021529

[29] Patel J.B. 16S rRNA gene sequencing for bacterial pathogen identification in the clinical laboratory. Mol. Diagn. 2001; 6:313–321. 11774196 10.1054/modi.2001.29158

[30] Ackerman C.M. , Myhrvold C., Thakku S.G., Freije C.A., Metsky H.C., Yang D.K., Ye S.H., Boehm C.K., Kosoko-Thoroddsen T.-S.F., Kehe J. Massively multiplexed nucleic acid detection with Cas13. Nature. 2020; 582:277–282. 32349121 10.1038/s41586-020-2279-8 PMC7332423

[31] Marras S.A. , Tyagi S., Antson D.-O., Kramer F.R. Color-coded molecular beacons for multiplex PCR screening assays. PLoS One. 2019; 14:e0213906. 30883590 10.1371/journal.pone.0213906 PMC6422326

[32] Rajagopal A. , Yurk D., Shin C., Menge K., Jacky L., Fraser S., Tombrello T.A., Tsongalis G.J. Significant expansion of real-time PCR multiplexing with traditional chemistries using amplitude modulation. Sci. Rep. 2019; 9:1053. 30705333 10.1038/s41598-018-37732-y PMC6355831

[33] Jacky L. , Yurk D., Alvarado J., Leatham B., Schwartz J., Annaloro J., MacDonald C., Rajagopal A. 2021) Virtual-partition digital PCR for high-precision chromosomal counting applications. Anal. Chem. 93:17020–17029. 34905685 10.1021/acs.analchem.1c03527

[34] Holzschuh A. , Lerch A., Gerlovina I., Fakih B.S., Al-mafazy A.-w.H., Reaves E.J., Ali A., Abbas F., Ali M.H., Ali M.A. et al. Multiplexed ddPCR-amplicon sequencing reveals isolated Plasmodium falciparum populations amenable to local elimination in Zanzibar, Tanzania. Nat. Commun. 2023; 14:3699. 37349311 10.1038/s41467-023-39417-1 PMC10287761

[35] Whale A.S. , Huggett J.F., Tzonev S. Fundamentals of multiplexing with digital PCR. Biomol. Detect. Quant. 2016; 10:15–23. 10.1016/j.bdq.2016.05.002 PMC5154634 27990345

[36] Huang Q. , Chen D., Du C., Liu Q., Lin S., Liang L., Xu Y., Liao Y., Li Q. Highly multiplex PCR assays by coupling the 5′-flap endonuclease activity of Taq DNA polymerase and molecular beacon reporters. Proc. Natl. Acad. Sci. U.S.A. 2022; 119:e2110672119. 35197282 10.1073/pnas.2110672119 PMC8892341

[37] Wu L.R. , Chen S.X., Wu Y., Patel A.A., Zhang D.Y. Multiplexed enrichment of rare DNA variants via sequence-selective and temperature-robust amplification. Nat. Biomed. Eng. 2017; 1:714–723. 29805844 10.1038/s41551-017-0126-5 PMC5969535

[38] Song P. , Chen S.X., Yan Y.H., Pinto A., Cheng L.Y., Dai P., Patel A.A., Zhang D.Y. Selective multiplexed enrichment for the detection and quantitation of low-fraction DNA variants via low-depth sequencing. Nat. Biomed. Eng. 2021; 5:690–701. 33941896 10.1038/s41551-021-00713-0 PMC9631981

[39] Zhang D.Y. , Chen S.X., Yin P. Optimizing the specificity of nucleic acid hybridization. Nat. Chem. 2012; 4:208–214. 22354435 10.1038/nchem.1246 PMC4238961

[40] Dai P. , Wu L.R., Chen S.X., Wang M.X., Cheng L.Y., Zhang J.X., Hao P., Yao W., Zarka J., Issa G.C. et al. Calibration-free NGS quantitation of mutations below 0.01% VAF. Nat. Commun. 2021; 12:6123. 34675197 10.1038/s41467-021-26308-6 PMC8531361

[41] Alvarez M. , Mendoza M.C. Molecular epidemiology of two genes encoding 3-N-aminoglycoside acetyltransferases AAC (3)I and AAC (3)II among gram-negative bacteria from a Spanish hospital. Eur. J. Epidemiol. 1993; 9:650–657. 8150069 10.1007/BF00211441

[42] Xie N.G. , Wang M.X., Song P., Mao S., Wang Y., Yang Y., Luo J., Ren S., Zhang D.Y. Designing highly multiplex PCR primer sets with Simulated Annealing Design using Dimer Likelihood Estimation (SADDLE). Nat. Commun. 2022; 13:1881. 35410464 10.1038/s41467-022-29500-4 PMC9001684

[43] Chotiprasitsakul D. , Han J.H., Cosgrove S.E., Harris A.D., Lautenbach E., Conley A.T., Tolomeo P., Wise J., Tamma P.D., Group A.R.L. Comparing the outcomes of adults with Enterobacteriaceae bacteremia receiving short-course versus prolonged-course antibiotic therapy in a multicenter, propensity score–matched cohort. Clin. Infect. Dis. 2018; 66:172–177. 29190320 10.1093/cid/cix767 PMC5849997

[44] Doualeh M. , Payne M., Litton E., Raby E., Currie A. Molecular methodologies for improved polymicrobial sepsis diagnosis. Int. J. Mol. Sci. 2022; 23:4484. 35562877 10.3390/ijms23094484 PMC9104822

[45] Lin J.N. , Lai C.H., Chen Y.H., Chang L.L., Lu P.L., Tsai S.S., Lin H.L., Lin H.H. Characteristics and outcomes of polymicrobial bloodstream infections in the emergency department: a matched case–control study. Acad. Emerg. Med. 2010; 17:1072–1079. 21040108 10.1111/j.1553-2712.2010.00871.x

[46] Pammi M. , Zhong D., Johnson Y., Revell P., Versalovic J. Polymicrobial bloodstream infections in the neonatal intensive care unit are associated with increased mortality: a case-control study. BMC Infect. Dis. 2014; 14:390. 25022748 10.1186/1471-2334-14-390 PMC4226990

[47] Park S.Y. , Park K.-H., Bang K.M., Chong Y.P., Kim S.-H., Lee S.-O., Choi S.-H., Jeong J.-Y., Woo J.H., Kim Y.S. Clinical significance and outcome of polymicrobial Staphylococcus aureus bacteremia. J. Infect. 2012; 65:119–127. 22410381 10.1016/j.jinf.2012.02.015

[48] Pavlaki M. , Poulakou G., Drimousis P., Adamis G., Apostolidou E., Gatselis N.K., Kritselis I., Mega A., Mylona V., Papatsoris A. Polymicrobial bloodstream infections: epidemiology and impact on mortality. J. Global Antimicrob. Resist. 2013; 1:207–212. 10.1016/j.jgar.2013.06.005 27873614

[49] Rodríguez-Baño J. , López-Prieto M.D., Portillo M.M., Retamar P., Natera C., Nuño E., Herrero M., Del Arco A., Muñoz Á., Téllez F. Epidemiology and clinical features of community-acquired, healthcare-associated and nosocomial bloodstream infections in tertiary-care and community hospitals. Clin. Microbiol. Infect. 2010; 16:1408–1413. 19845694 10.1111/j.1469-0691.2009.03089.x

[50] Tsai M.-H. , Chu S.-M., Hsu J.-F., Lien R., Huang H.-R., Chiang M.-C., Fu R.-H., Lee C.-W., Huang Y.-C. Polymicrobial bloodstream infection in neonates: microbiology, clinical characteristics, and risk factors. PLoS One. 2014; 9:e83082. 24454692 10.1371/journal.pone.0083082 PMC3891628

[51] Zheng C. , Zhang S., Chen Q., Zhong L., Huang T., Zhang X., Zhang K., Zhou H., Cai J., Du L. Clinical characteristics and risk factors of polymicrobial Staphylococcus aureus bloodstream infections. Antimicrob. Resist. Infect. Control. 2020; 9:76. 32460851 10.1186/s13756-020-00741-6 PMC7254655

[52] Pincus D.H. Microbial identification using the bioMérieux Vitek® 2 system. Encyclopedia of Rapid Microbiological Methods. 2006; 2006:Bethesda, MD Parenteral Drug Association 1–32.

[53] Petrosino J.F. , Highlander S., Luna R.A., Gibbs R.A., Versalovic J. Metagenomic pyrosequencing and microbial identification. Clin. Chem. 2009; 55:856–866. 19264858 10.1373/clinchem.2008.107565 PMC2892905

[54] Fang H. , Ohlsson A.K., Ullberg M., Özenci V. Evaluation of species-specific PCR, Bruker MS, VITEK MS and the VITEK 2 system for the identification of clinical Enterococcus isolates. Eur. J. Clin. Microbiol. Infect. Dis. 2012; 31:3073–3077. 22706514 10.1007/s10096-012-1667-x

[55] Singhal N. , Kumar M., Kanaujia P.K., Virdi J.S. MALDI-TOF mass spectrometry: an emerging technology for microbial identification and diagnosis. Front. Microbiol. 2015; 6:791. 26300860 10.3389/fmicb.2015.00791 PMC4525378

[56] Rudd K.E. , Johnson S.C., Agesa K.M., Shackelford K.A., Tsoi D., Kievlan D.R., Colombara D.V., Ikuta K.S., Kissoon N., Finfer S. et al. Global, regional, and national sepsis incidence and mortality, 1990-2017: analysis for the Global Burden of Disease Study. Lancet. 2020; 395:200–211. 31954465 10.1016/S0140-6736(19)32989-7 PMC6970225

[57] Hajj J. , Blaine N., Salavaci J., Jacoby D. The “centrality of sepsis”: a review on incidence, mortality, and cost of care. Healthcare (Basel). 2018; 6:90–100. 30061497 10.3390/healthcare6030090 PMC6164723

[58] Park J.S. , Pisanic T., Zhang Y., Wang T.H. Ligation-enabled fluorescence-coding PCR for high-dimensional fluorescence-based nucleic acid detection. Anal. Chem. 2021; 93:2351–2358. 33427441 10.1021/acs.analchem.0c04221 PMC8574133

[59] Liao Y. , Wang X., Sha C., Xia Z., Huang Q., Li Q. Combination of fluorescence color and melting temperature as a two-dimensional label for homogeneous multiplex PCR detection. Nucleic Acids Res. 2013; 41:e76. 23335787 10.1093/nar/gkt004 PMC3627564

[60] Dewald F. , Suarez I., Johnen R., Grossbach J., Moran-Tovar R., Steger G., Joachim A., Rubio G.H., Fries M., Behr F. et al. Effective high-throughput RT-qPCR screening for SARS-CoV-2 infections in children. Nat. Commun. 2022; 13:3640. 35752615 10.1038/s41467-022-30664-2 PMC9233713

[61] Milbury C.A. , Creeden J., Yip W.K., Smith D.L., Pattani V., Maxwell K., Sawchyn B., Gjoerup O., Meng W., Skoletsky J. et al. Clinical and analytical validation of FoundationOne (R)CDx, a comprehensive genomic profiling assay for solid tumors. PLoS One. 2022; 17:e0264138. 35294956 10.1371/journal.pone.0264138 PMC8926248

[62] Sakata S. , Otsubo K., Yoshida H., Ito K., Nakamura A., Teraoka S., Matsumoto N., Shiraishi Y., Haratani K., Tamiya M. et al. Real-world data on NGS using the Oncomine DxTT for detecting genetic alterations in non-small-cell lung cancer: WJOG13019L. Cancer Sci. 2022; 113:221–228. 34704312 10.1111/cas.15176 PMC8748216

[63] Frickmann H. , Künne C., Hagen R.M., Podbielski A., Normann J., Poppert S., Looso M., Kreikemeyer B. Next-generation sequencing for hypothesis-free genomic detection of invasive tropical infections in poly-microbially contaminated, formalin-fixed, paraffin-embedded tissue samples–a proof-of-principle assessment. BMC Microbiol. 2019; 19:75. 30961537 10.1186/s12866-019-1448-0 PMC6454699

[64] Avershina E. , Frye S.A., Ali J., Taxt A.M., Ahmad R. Ultrafast and cost-effective pathogen identification and resistance gene detection in a clinical setting using Nanopore Flongle sequencing. Front. Microbiol. 2022; 13:822402. 35369431 10.3389/fmicb.2022.822402 PMC8970966

