# Correction to ‘Advancing quantitative PCR with color cycle multiplex amplification’

**DOI:** 10.1093/nar/gkae827

**Published:** 2024-09-18

This is a correction to: Wei Chen, Kerou Zhang, Fei Huang, Lan Zhao, George C Waldren, Qi Jiang, Sherry X Chen, Bonnie Wang, Wei Guo, David Y Zhang, Jinny X Zhang, Advancing quantitative PCR with color cycle multiplex amplification, *Nucleic Acids Research*, 2024;, gkae683, https://doi.org/10.1093/nar/gkae683

This manuscript has been amended to correct the affiliation of the fourth-listed author, Lan Zhao.

The author's affiliation was originally stated as follows:

Department of Respiratory Diseases, Shanghai Pulmonary Hospital, Tongji University School of Medicine, Shanghai, Shanghai 200433, China.

The affiliation has been corrected to:

Department of Respiratory and Critical Care Medicine, Shanghai Pulmonary Hospital, School of Medicine, Tongji University, Shanghai 200433, China.

