# Supplementary material for: Advancing quantitative PCR with color cycle multiplex amplification
Source: Nucleic Acids Res. 2024 Aug 9;52(17):e81. doi: 10.1093/nar/gkae683 (PMC11417387; doi:10.1093/nar/gkae683)
Supplement: gkae683_Supplemental_Files [file gkae683_supplemental_files.zip › NAR-CCMA-SI-20240610-FINAL.pdf]

Supplementary Materials for

Advancing Quantitative PCR with Color Cycle Multiplex Amplification

Wei Chen *et al.*

*Corresponding author. Email: jinnychang92@gmail.com, genomic.dave@gmail.com, guo.wei@zs-hospital.sh.cn

Section 1 Comparison of multiplex qPCR approaches ... 2
Section 2 Proof of concept and design principle of color cycle multiplex amplification ... 4
Section 3 Primer design and primer specificity validation ... 6
Section 4 Data analysis ... 10
Section 5 Panel development and experiments optimization ... 11
Section 6 Panel performance: analytical LoD results ... 14
Section 7 Quantification through the Ct value of the first color ... 16
Section 8 Robustness ... 18
Section 9 Quantitative testing of pathogen genomic DNA samples from clinical samples ... 21
Section 10 CCMA applied to multiple-target-present situation ... 25
Section 11 CCMA applied to clinical samples ... 27
Section 12 Supplementary Tables ... 30

Section 1 Comparison of multiplex qPCR approaches

qPCR and color cycle multiplex amplification (CCMA) have similar low costs and quick turnaround time. However, CCMA multiplexity is not limited by the 6 colors available on most commercially available qPCR instruments as it leverages permutations of colors. Several techniques have been developed to increase the number of possible DNA target sequences in a single qPCR assay. For example, color mixing interprets two fluorescent signals from a single target as a unique target, resulting in 3 possible targets for 2 colors (Supplementary Figure 1a). CCMA utilizes rationally designed blockers to introduce programmable Ct delays (timings), as seen in Figure 1a and Supplementary Figure 1b. These timings result in more potential DNA sequences identified by conventional qPCR instrumentation. More blockers can be added to increase the number of timings and number of targets. Given 3 channels and 1 timing, a total of 9 targets can be identified (Supplementary Figure 1c).

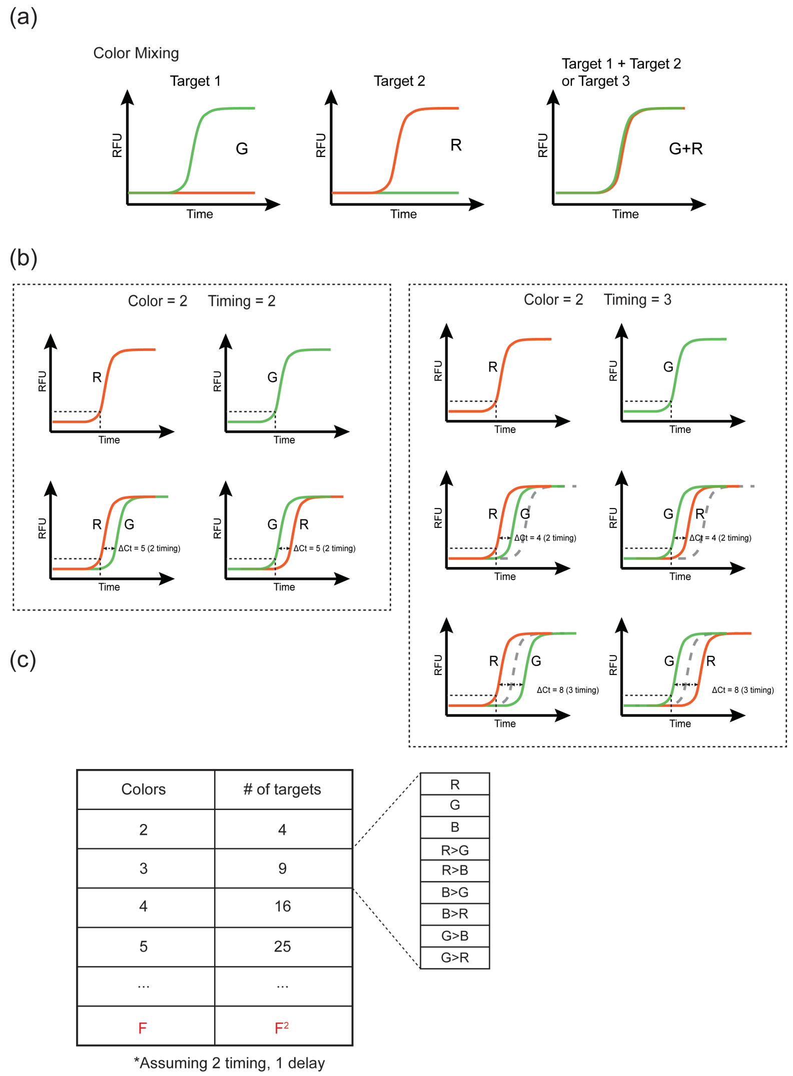

Colors	# of targets
2	4
3	9
4	16
5	25
…	…
F	F^2

Supplementary Figure 1. Color cycle multiplex amplification (CCMA) leverages fluorescence permutation instead of color combination to increase assay multiplexity. (a) Diagram illustrating the concept of color mixing. Color mixing uses different color combinations to identify different targets. (b) Introduction of programmable delays (timings) further increases the number of DNA targets identifiable by CCMA. Grey dashed

line indicates the virtual timing. (c) Number of possible targets identifiable by CCMA given 2 timing.

Section 2 Proof of concept and design principle of color cycle multiplex amplification

Blocker design is flexible and can be fine-tuned to yield different $\Delta G°_{rxn}$. To determine the optimal sequences, blockers with various $\Delta G°_{rxn}$ are generated, ranging between 0.09-4.84 kcal mol^{-1}. Equation 1 gives the thermodynamic favorability of the binding of the template sequence, $\Delta G°_{rxn}$, by the blocker, $\Delta G°_{NH}$, over the primer, $\Delta G°_{toehold}$.

$$\Delta G°_{\text{rxn}} = \Delta G°_{\text{NH}} - \Delta G°_{\text{toehold}} \quad (1)$$

Lower $\Delta G°_{rxn}$ values indicate increased binding affinity between the blocker and the template DNA, resulting in greater expected Ct delays. Designing blockers to yield more negative $\Delta G°_{rxn}$ reduces amplification efficiency and delays Ct for qPCR reactions with blockers compared to a blocker-free reaction. Additional blockers can be designed and introduced to further decrease $\Delta G°_{rxn}$, leading to increased delays in fluorescence signal relative to other amplicon reactions. Thus, by assigning multiple probes and designing multiple blockers with different $\Delta G°_{rxn}$ for different regions on a single target, the order in which each channel achieves Ct can be manipulated and becomes an additional parameter for target identification.

To demonstrate the viability of color cycle multiplex amplification (CCMA), we designed forward and reverse primers for an *aac(3)-II* sequence. This target was chosen as it confers antibiotic resistance to many bacterial species, resulting in its high prevalence. TaqMan probes were uniquely labelled with the fluorophores FAM, HEX, Cy5, and ROX, and were used to monitor amplification of templates. The resulting qPCR amplification curve is given in Supplementary Figure 2. Relationship between $\Delta G°_{rxn}$ and Ct delay is shown in Supplementary Figure 3. The concentration of the blocker can also be manipulated to further adjust $\Delta G°_{rxn}$. (Supplementary Figure 4).

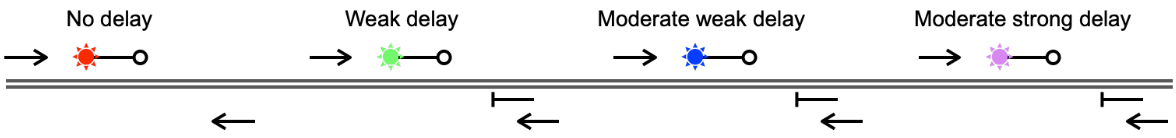

Channel	Ct avg	Ct delays
T1-FAM	18.00	
T2-HEX	21.25	3.25
T3-Cy5	26.75	5.50
T4-ROX	33.00	6.25

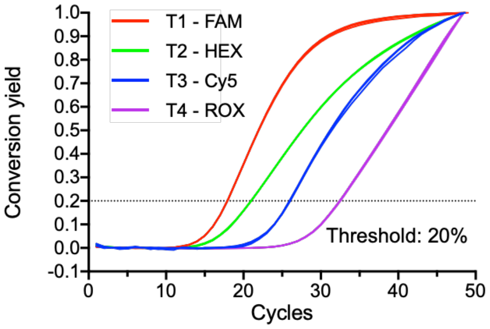

Supplementary Figure 2. Proof of concept: single target performance. 4 amplicons with different fluorophores are designed to label the same pathogen target. A desired fluorescence order for this target can be obtained by optimizing blocker selection. Nucleic acid sequences are given in Tables S1. qPCR was performed according to parameters in Supplementary Table 8. Ct delays are calculated were calculated by subtracting from the Ct average of the fluorophore with the closest lower Ct value.

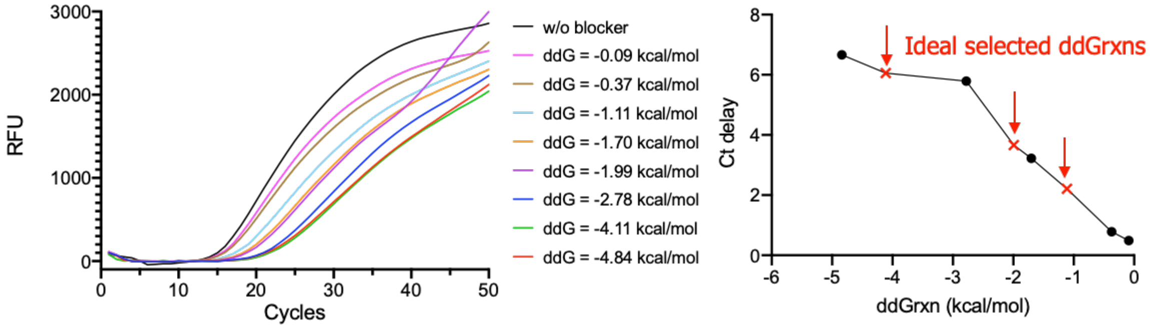

Supplementary Figure 3. Relationship between ΔG_{rxn} and Ct delay. qPCR was performed using different blockers with various ΔG_{rxn} for an *aac(3)-II* template. The ΔG_{rxn} were then plotted against the resulting Ct delay of the qPCR reaction. Red arrows point to ideal selected blockers. Nucleic acid sequences are given in Supplementary Table 1. qPCR was performed according to parameters in Supplementary Table 8.

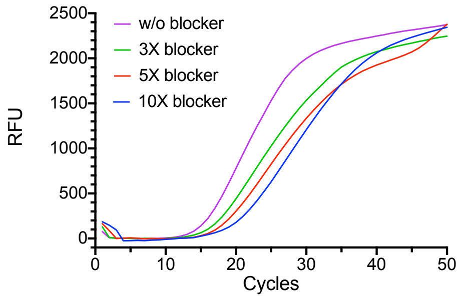

Supplementary Figure 4. Relationship between blocker concentration and Ct delay. 3X/5X/10X represents the ratio of concentration of blocker sequences to reverse primers sequences is 3/5/10 respectively. Nucleic acid sequences are given in Supplementary Table 1. qPCR was performed according to parameters in Supplementary Table 8.

Section 3 Primer design and primer specificity validation

As shown in Supplementary Figure 5, the whole genome sequences of the microbial targets of interest were first split into 300 bp segments and then further split into five 100 bp sub-segments which were subsequently aligned by Basic Local Alignment Search Tool (BLAST) against a database containing the genomes of all target pathogens, to minimize potential overlap between binding regions. For each target species, we then selected three 300 bp fragments that passed both rounds of filtering and applied our previously validated Simulated Annealing Design using Dimer Likelihood Estimation (SADDLE) algorithm to the selected sequences to obtain primers for multiplexed PCR. For each target amplicon, Taqman probes and blocker sequences with different Gibbs free binding energies were also designed using an internal algorithm.

The distribution of the length of all 63 designed amplicons are shown in Supplementary Figure 7. Primers were first validated for specificity using contrived samples. gBlock containing DNA targets were spiked into human gDNA and amplified (Supplementary Figure 8).

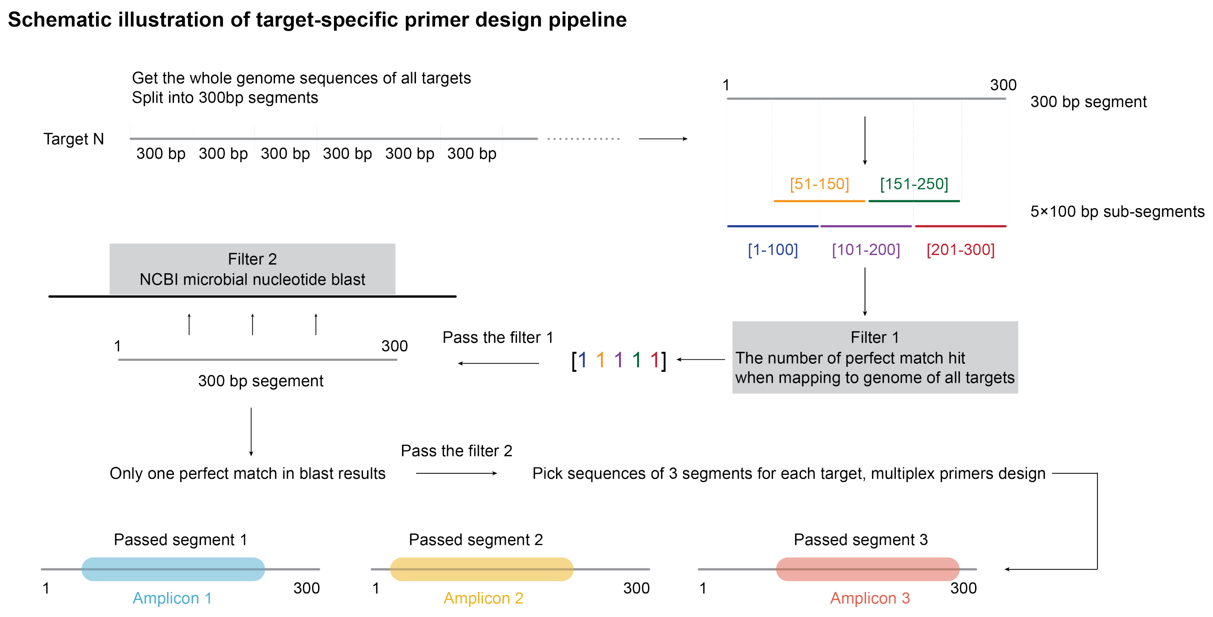

Supplementary Figure 5. Sequence specificity of multiplexed target detection. Schematic illustration of target specific primer design pipeline.

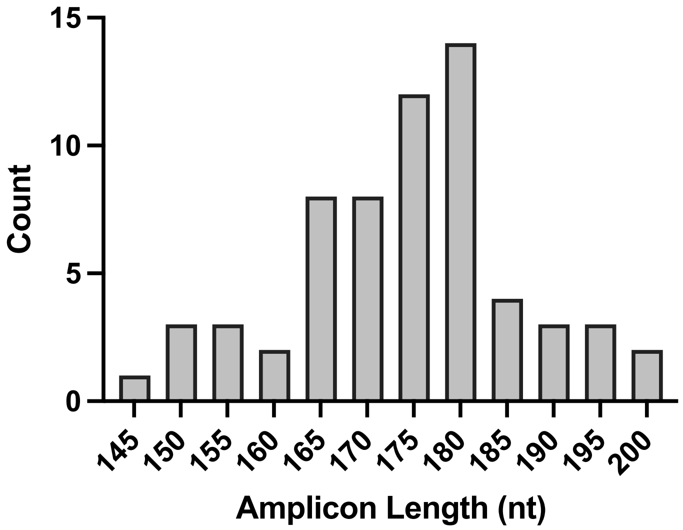

Supplementary Figure 6. Histogram of amplicon length distribution. The length of 63 amplicons ranges from 145 to 200 nt, with a median length of 175 nt.

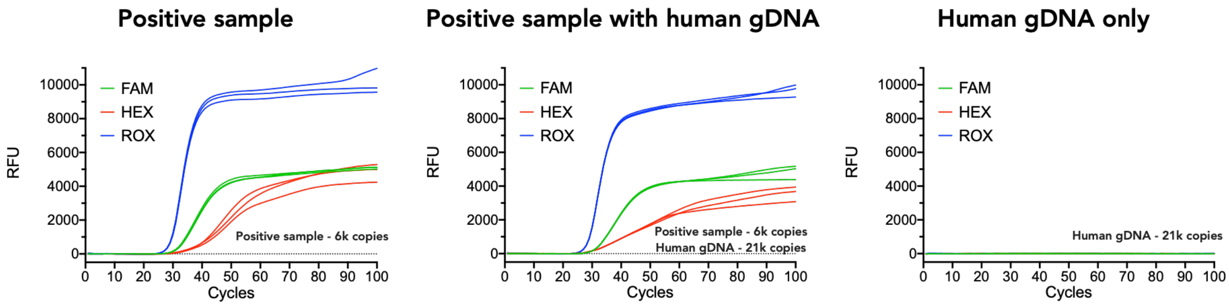

Supplementary Figure 7. Optimized primer design enables highly specific and sensitive detection of bacterial target DNA. Samples with a high concentration of human gDNA will not compromise assay performance. Nucleic acid sequences are given in Supplementary Table 1. qPCR was performed according to parameters in Supplementary Table 8.

Minimum mismatch nucleotide nucleotides are given. Primer dimers, off-target amplifications and on-target amplifications are outlined in the heatmap in Supplementary Figure 8.

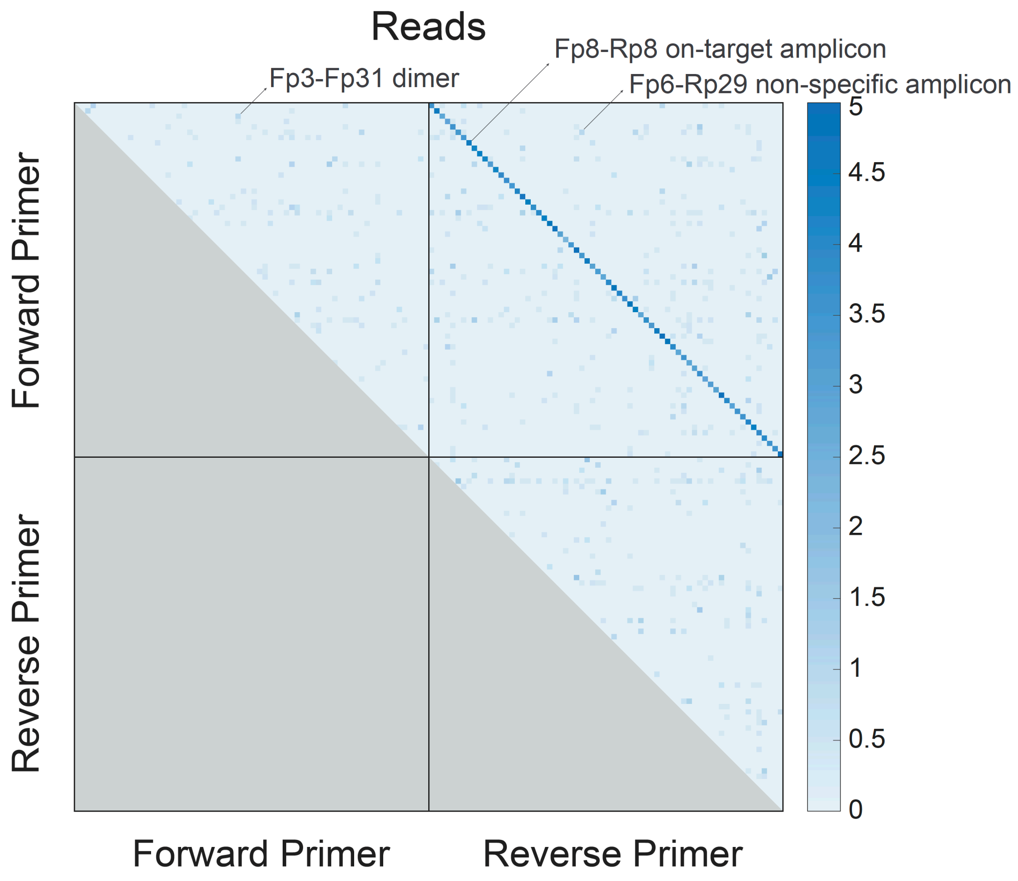

Supplementary Figure 8. Next-generation sequencing (NGS)-based validation of primer specificity. Multiplexed primers and gBlocks of all 21 bacterial species were combined with PCR master mix. After amplification, the PCR products were purified, end-repaired, adapter-ligated, indexed, and sequenced. The number of reads (log-scaled) for each on-target amplicon, primer dimer pair, or non-specific amplicon formed by the combinations of forward and reverse primers are shown in the heatmap.

Genomic Specificity of Primers

Minimum mismatch nucleotide number of each primer blast to 21 microbial sequence database excluding target species

e.g. 1

S. aureus forward primer 1

GCAAGCATTGACGAGTCCTATACAT

•••TCGTAACT•CTCAGGA••••••

E. coli sequence

Mismatch nucleotide numer: 10

e.g. 2

E. coli forward primer 1

CGCGAATAAAGCAGCAGAAGC

•CGCTT•TTTCGTCGTCT•••

S. saprophyticus sequence

Mismatch nucleotide numer: 5

Target Species	Forward Primer 1	Reverse Primer 1	Forward Primer 2	Reverse Primer 2	Forward Primer 3	Reverse Primer3
Staphylococcus aureus	10	3	19	5	16	4
Staphylococcus epidermidis	5	9	11	13	6	9
Staphylococcus capitis	12	10	10	3	19	13
Staphylococcus haemolyticus	8	9	8	11	10	4
Staphylococcus lugdunensis	4	7	5	7	5	7
Streptococcus pneumoniae	7	10	26	8	7	4
Streptococcus agalactiae	7	3	4	11	11	13
Acinetobacter baumannii	20	16	5	5	10	14
Citrobacter freundii	10	14	13	11	7	12
Clostridium sporogenes	12	10	10	3	19	13
Chlamydia pneumoniae	6	12	5	6	7	10
Escherichia coli	4	6	6	8	3	5
Enterococcus faecium	6	12	10	10	4	11
Enterococcus faecalis	13	6	7	4	8	9
Legionella spiritensis	9	8	3	1	7	7
Listeria monocytogenes	10	8	8	10	5	7
Klebsiella pneumoniae	1	5	5	5	7	9
Neisseria meningitidis	13	2	4	4	2	4
Pseudomonas aeruginosa	4	3	8	7	5	4
Proteus mirabilisitensis	4	9	11	11	2	10
Salmonella entericatogenes	4	2	3	4	12	5

Supplementary Figure 9. Genome specificity of the primers. Minimum mismatch nucleotide number of each primer blast to 21 microbial sequences database excluding target species.

Section 4 Data analysis

Raw data from the qPCR instrument were initially subjected to baseline correction:

Perform baseline correction

iff the slope of linear fitting of RFU (cycles 10-20) is greater than 1.5 or lower than -1.5.

Baseline correction consisted of creating a new linear fitting curve based on the slope given from cycles 10-20. Once the baseline correction was applied, Ct for each color was determined in the signal output step using either fixed or dynamic Ct calling.

Perform dynamic Ct calling

iff End-point RFU is lower than 100.

For cases in which Ct calling was applicable, the cycle at which 10% fluorescence plateau was reached was used in place of Ct. Following this, color output was assigned for each species based on Ct. Only the first 3 signals will be outputted.

In the majority of cases, approximately 95%, raw data are not subjected to baseline correction or dynamic Ct calling. Consequently, it is straightforward to obtain Ct values directly from the qPCR instrument. See Supplementary Figure 10 for detailed illustration of qPCR curve changes after baseline correction.

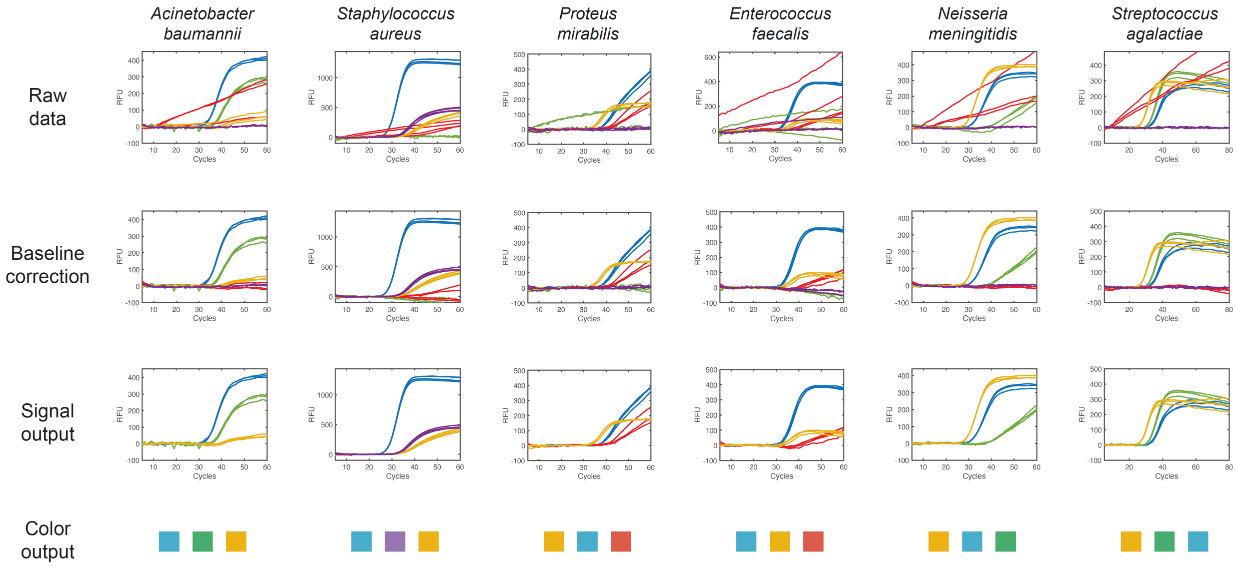

Supplementary Figure 10. Examples of the data analysis process. Data analysis workflow for 6 bacterial species is shown.

Section 5 Panel development and experiments optimization

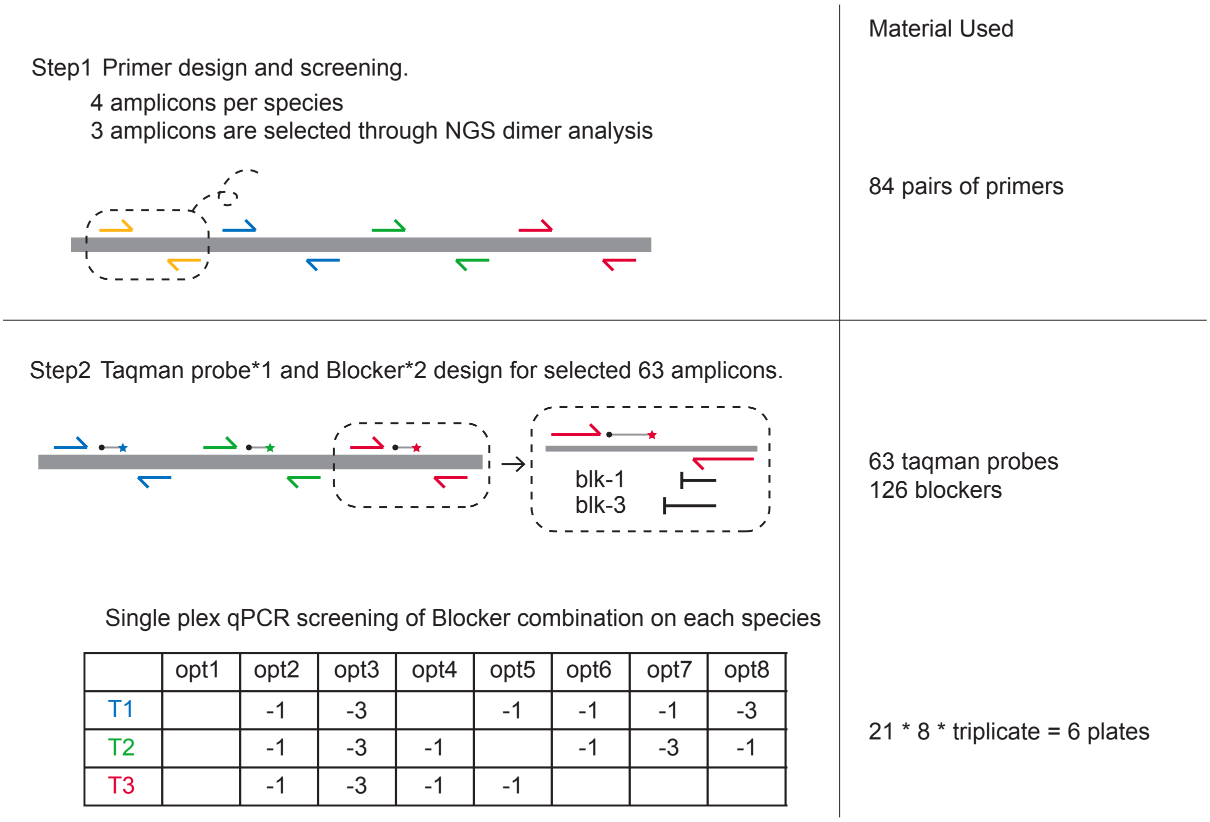

	opt1	opt2	opt3	opt4	opt5	opt6	opt7	opt8
T1		-1	-3		-1	-1	-1	-3
T2		-1	-3	-1		-1	-3	-1
T3		-1	-3	-1	-1			

Supplementary Figure 11. Illustration of streamlined CCMA panel development and optimization workflow.

To demonstrate that developing higher-plex CCMA panels is feasible without excessive cost or labor, we have outlined a streamlined assay design and optimization workflow (Supplemental Figure 11). This leverages computational tools for rapid in silico modeling to minimize experimental validation needs.

The detailed steps are listed:

Step 1: Automated multiplex primer design using our specially developed algorithm, SADDLE (https://doi.org/10.1038/s41467-022-29500-4), that maximizes specificity of each primer pair while minimizing risk of primer dimer formation. For each target species, 4 potential primer pairs are computationally selected. All candidate primers (84 pairs covering 21 species) are simultaneously amplified by multiplex PCR, then barcoded, pooled and sequenced via next generation sequencing. Only high performing primer pairs with robust on-target amplification and no detectable cross-reactivity or primer dimer formation are advanced to the next round (Figure 3A and Supplementary Figure 8-9). This results in 3 locked down primer pairs per species that can be further used for testing.

Step 2: Design of TaqMan probes and blockers. One TaqMan probe is designed per amplification sequence, targeting a conserved probe binding region. Additionally, two blocking oligonucleotides are designed flanking the primers for each set, with different ΔG_{rxn} roughly ranging from -1 kcal/mol to -3 kcal/mol. We methodically screened order of color combinations per target using the combinations provided in Supplementary Figure 11, totaling 8 single-plex tests per species performed in triplicate, enabling the screening process to be completed in just 6 plates for 21 species. Based on prior expertise with

blocker displacement amplification and Supplementary Figure 3 and 4, tuning of blocker binding energy provides coarse modulation of Ct delays and hence changing the color order, while adjusting blocker concentration can fine tune Ct delay in later steps. Upon completing Step 2, combinations of blockers, primers, probes, and color codes per species can be assigned. We have developed an automated blocker design tool that generates sequence based on Gibbs binding energy of DNA duplex. The source code can be shared upon request.

Step 3: Concentration optimization in multiplex setting. The concentrations of primers and blockers are both reduced to minimize PCR oligo loads in the multiplex system while maintaining the color orders for each target. The color order can be preserved when the primer to blocker ratio per assay is not dramatically altered. Thus, oligo mixes detecting multiple targets can be directly diluted together to optimize performance in batch to minimize workload in re-pooling. Blocker sequences are only changed from Step 2 when necessary to further tune Ct delays. In step 3, only blocker alteration, change of the oligo concentrations are performed. TaqMan probe sequences remain unchanged. According to our development record, only less than 5% of the blocker sequences are changed from Step 2, with 100% sequence preservation of primers and TaqMan probes.

Small-scale reactions rapidly screen conditions, enabling cost-effective translation to higher plex scales. Systematic concentration optimization preserves color order while minimizing redesign of assay components needed. In summary, well-developed computational design algorithm couples with targeted rapid prototyping could greatly simplify the development of higher-plex CCMA panels.

Take *E. coli* as an example, real-time fluorescence curves using blockers with different ΔG_{rxn} are shown in Supplementary Figure 12. Furthermore, Supplementary Figure 13 shows the qPCR curve for 21 bacterial DNA templates using optimal blocker sequences (Supplementary Table 7).

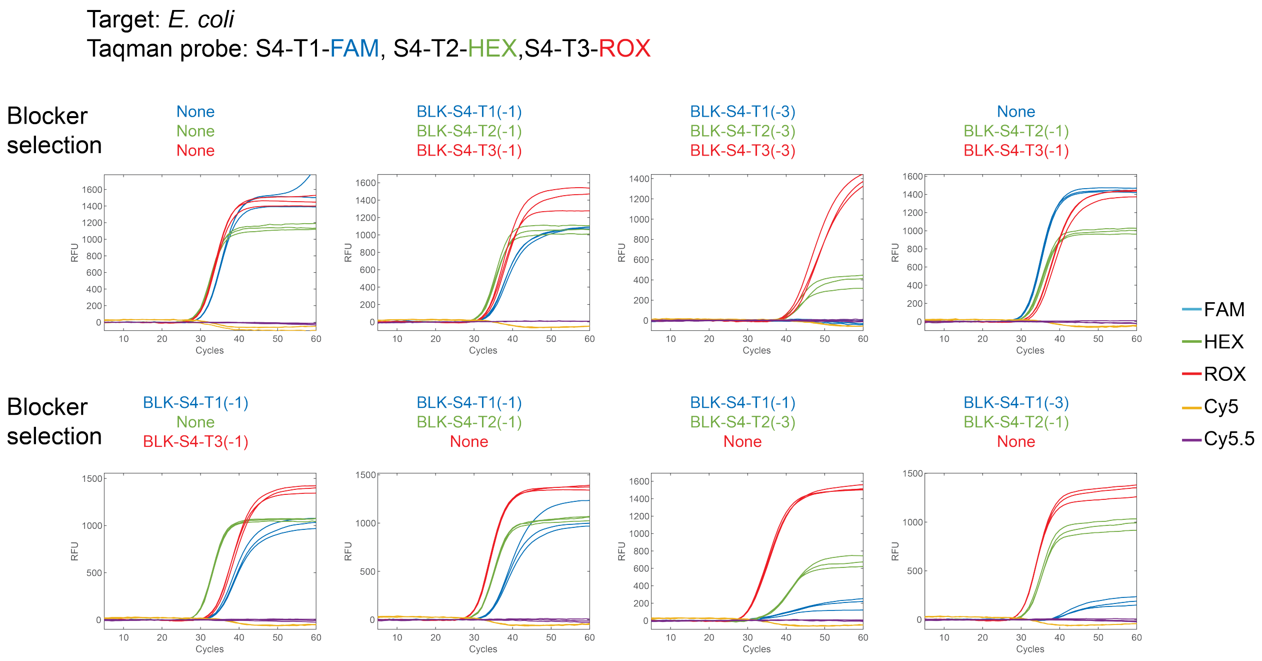

Supplementary Figure 12. Real-time fluorescence curves using *E. coli* gBlock DNA as the detection target and blockers with different binding energies (ΔGrxn ranging from -1 kcal /

mol to -3 kcal / mol) targeting different amplicons. Primer, probe, blocker, and template sequences are given in Supplementary Table 2-5; reagent concentrations are given in Supplementary Table 7. qPCR reaction was carried out under conditions described in Supplementary Table 8.

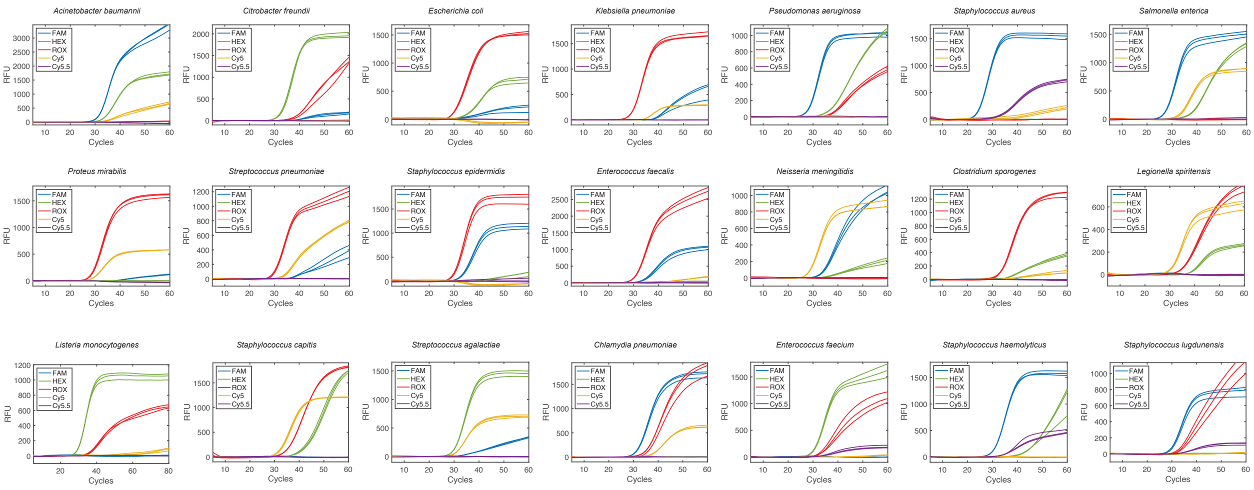

Supplementary Figure 13. Summary of single-plex color cycle multiplex amplification test results. Primer, probe, blocker, and template sequences are given in Supplementary Table 2-5; reagent concentrations are given in Supplementary Table 7. qPCR reaction was carried out under conditions described in Supplementary Table 8.

Section 6 Panel performance: analytical LoD results

The limits of detection differ for each bacterial DNA template. To ensure accurate detection, we determined the limits of detection (LoD) of our assay by performing CCMA in triplicate on decreasing copy numbers of the 21 bacterial gBlock templates. All templates could be detected at 30 copies.

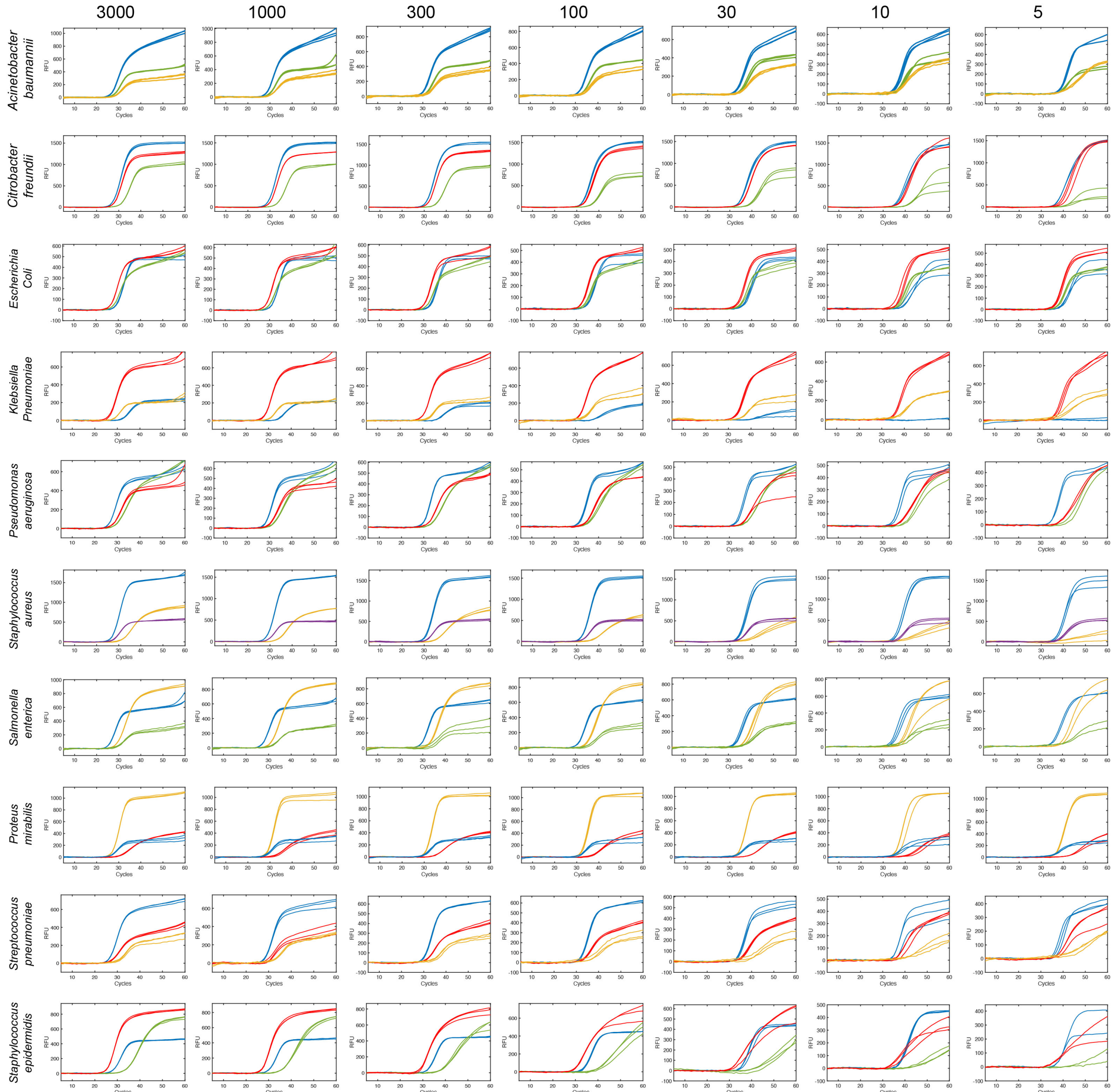

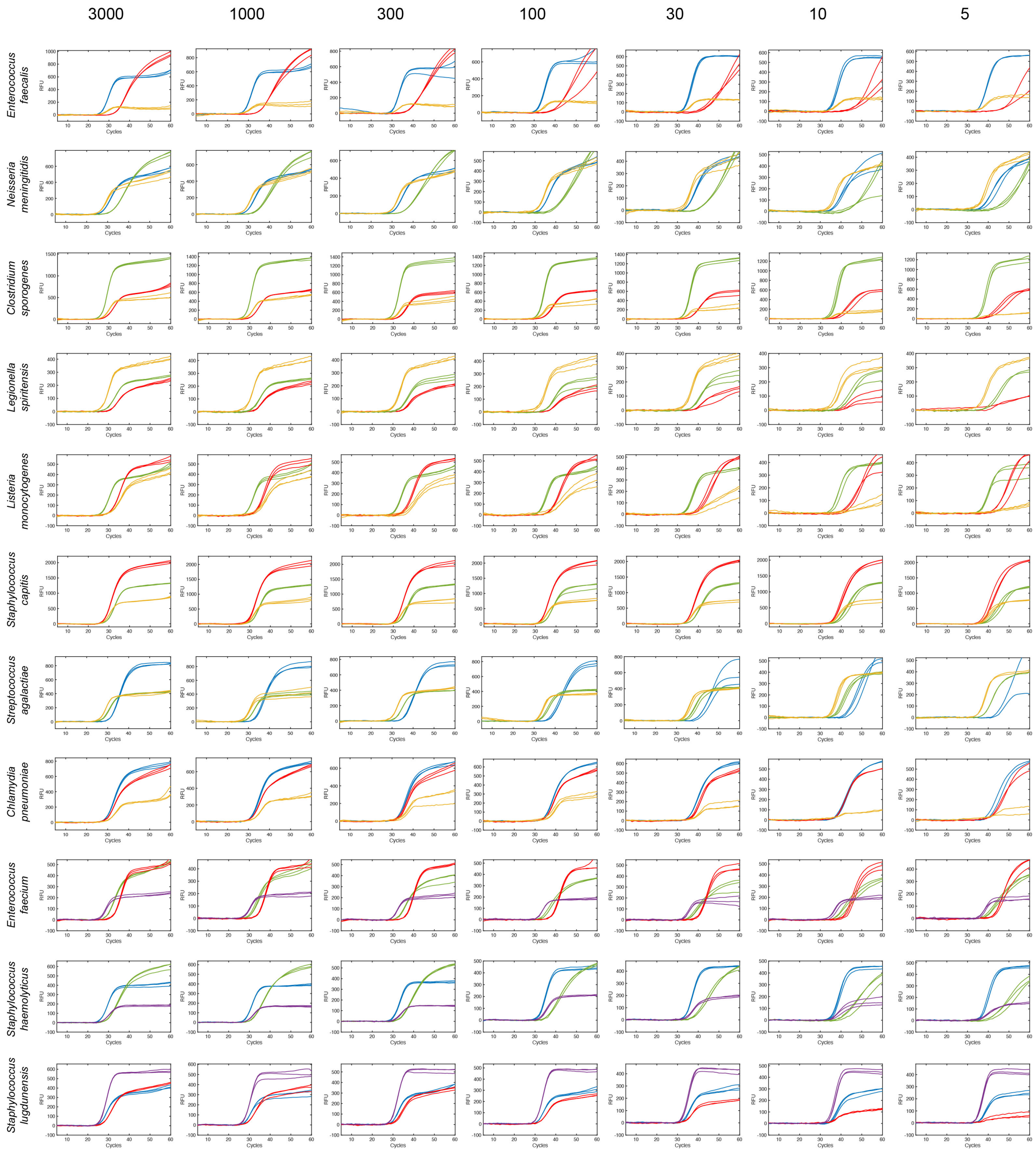

Supplementary Figure 14. Limits of detection for synthetic bacterial DNA sequences using a sepsis-related bacterial detection panel based on color cycle multiplex amplification. The real-time PCR curves corresponding to each species' lowest reliable copy number detection threshold using gBlock DNA are shown. Primer, probe, blocker, and template sequences are given in Supplementary Table 2-5; reagent concentrations are given in Supplementary Table 7. qPCR reaction was carried out under conditions described in Supplementary Table 8.

Section 7 Quantification through the Ct value of the first color

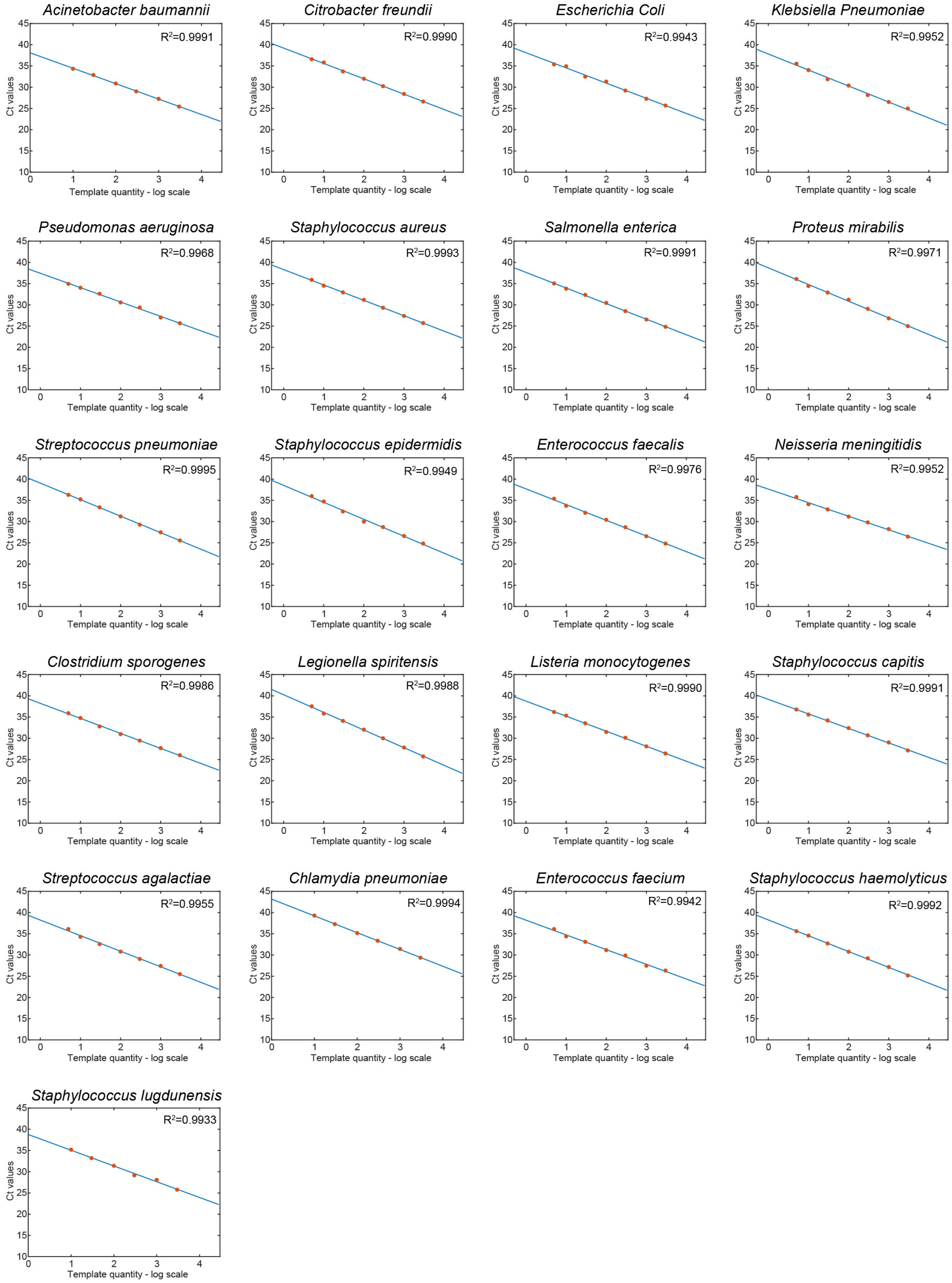

Supplementary Figure 15. Quantitative detection of gBlock DNA by CCMA using the first Ct value. For each target species, linear fitting results of the logarithm of the input quantities (copies) of gBlock DNA and the Ct value of the first fluorescence are shown. We can infer bacterial concentration from the first Ct value as it is not delayed by any blockers. These

values can be used to create a standard calibration curve and ensure accurate quantitation of DNA targets.

Section 8 Robustness

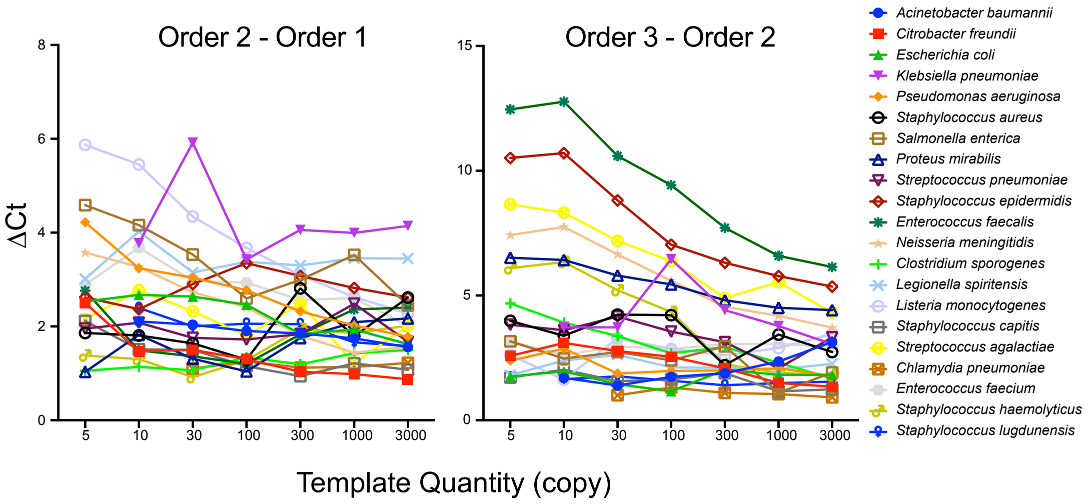

Supplementary Figure 16. Summary of the Ct differences between the first two order and the last two order for all species with different input quantity (5 copies to 3000 copies).

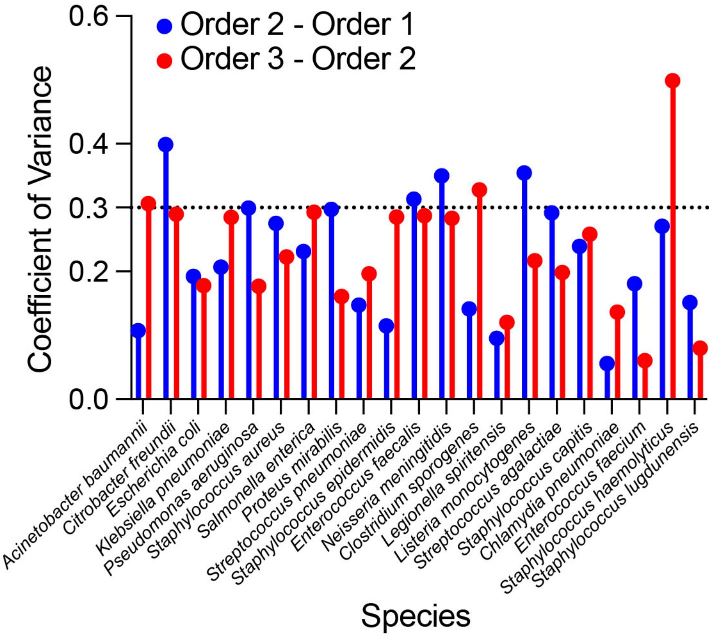

Supplementary Figure 17 Summary of coefficient of variance (CV) of Ct difference for each species with different input quantity. See detailed Ct difference in Supplementary Figure 16.

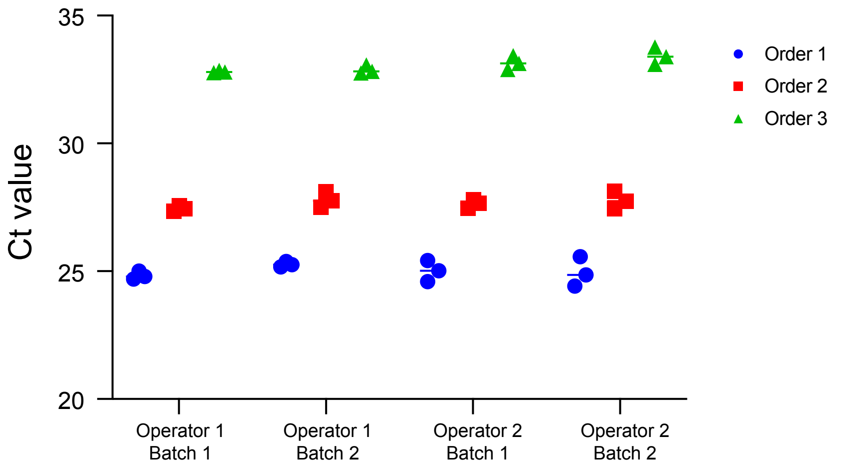

Supplementary Figure 18. Ct robustness of CCMA with different operators and reagent batches. *Staphylococcus epidermidis* with input quantity at 3000 copies were used as PCR template.

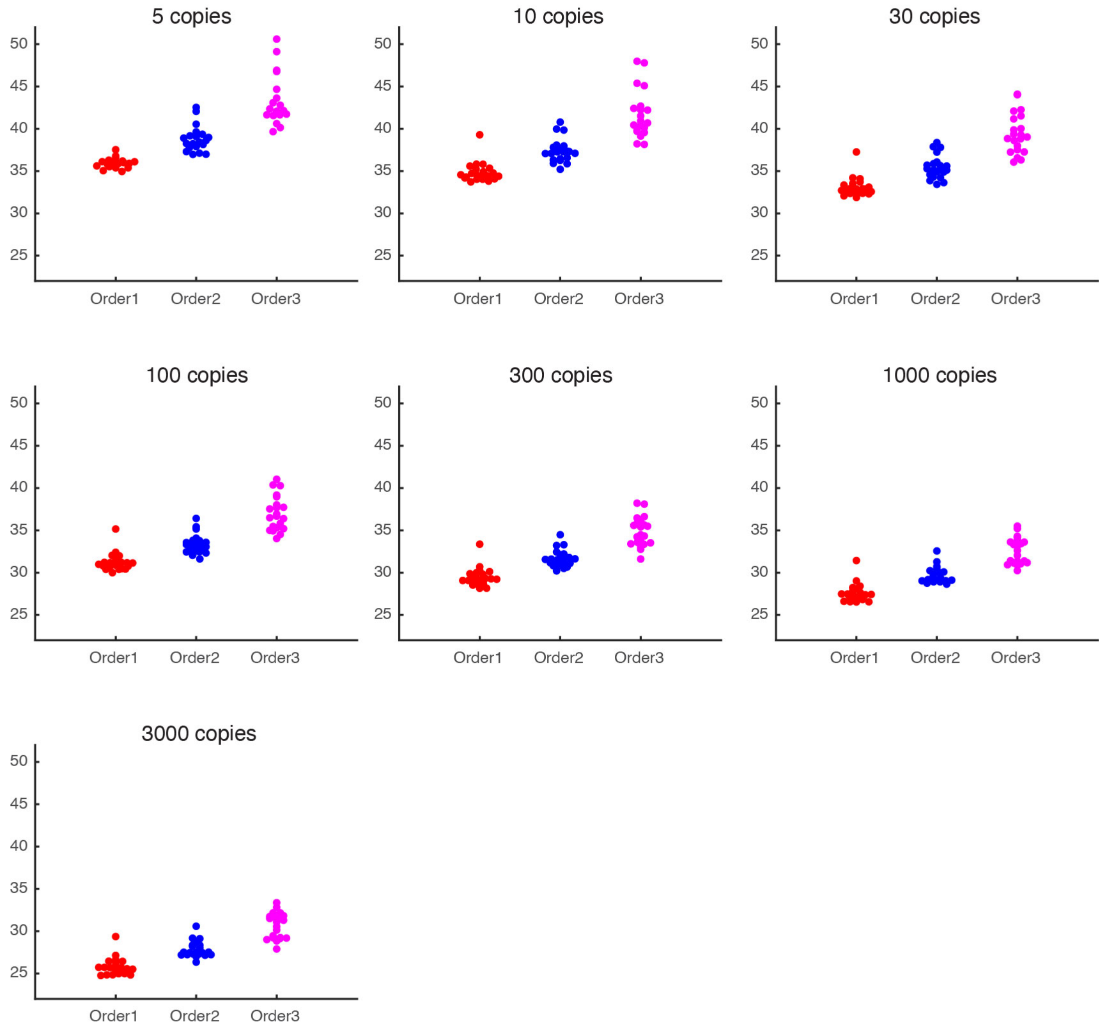

Supplementary Figure 19. Summary of Ct values of all 21 species and all color orders with different input quantity ranging from 5 copies to 3000 copies.

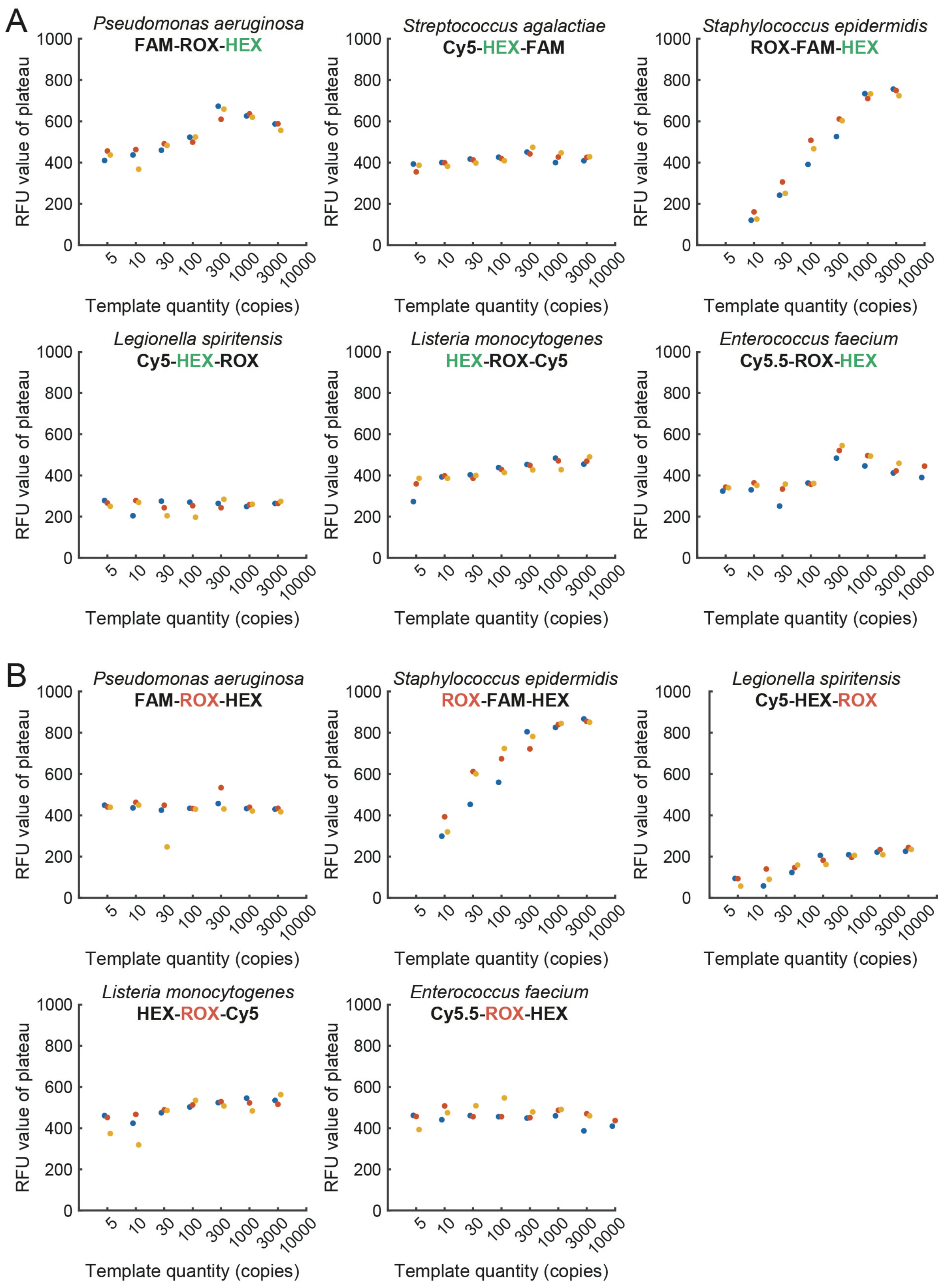

Supplementary Figure 20. Summary of end-point RFU of (A) HEX channel and (B) ROX channel. RFU values of plateau of the CCMA test of *Pseudomonas aeruginosa, Streptococcus agalactiae, Staphylococcus epidermidis, Legionella spiritensis, Listeria monocytogenes, Enterococcus faecium* with different template quantity (5 to 10000 copies) are plotted. 3 color dots represent 3 parallel experiments.

Section 9 Quantitative testing of pathogen genomic DNA samples from clinical samples

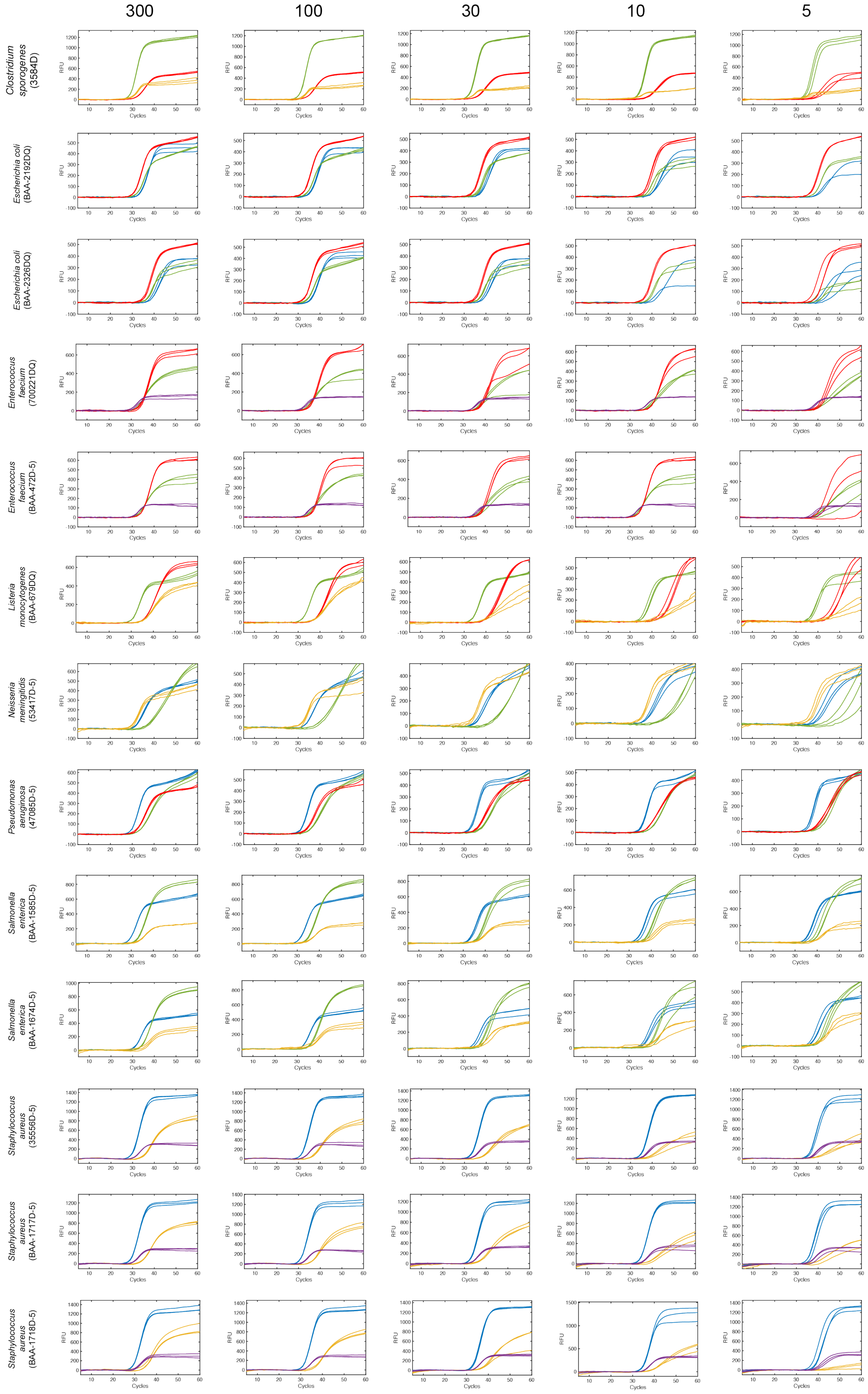

Supplementary Figure 21. Limits of detection for bacterial strain DNA using a sepsis-related bacterial detection panel based on color cycle multiplex amplification. The real-

time PCR curves corresponding to each species' lowest reliable copy number detection threshold using extracted bacterial genomic DNA (gDNA) are shown. Primer, probe, blocker, and template sequences are given in Supplementary Table 2-5; reagent concentrations are given in Supplementary Table 7. qPCR reaction was carried out under conditions described in Supplementary Table 8 and in materials and methods.

**Species Name**	**Strain (ATCC Catalog)**	**LoD (copies)**
Clostridium sporogenes	3584D	5
Enterococcus faecium	700221DQ	10
Enterococcus faecium	BAA-472D-5	10
Listeria monocytogenes	BAA-679DQ	5
Staphylococcus aureus	35556D-5	5
Staphylococcus aureus	BAA-1717D-5	5
Staphylococcus aureus	BAA-1718D-5	5
Escherichia coli	BAA-2192DQ	5
Escherichia coli	BAA-2326DQ	5
Neisseria meningitidis	53417D-5	5
Pseudomonas aeruginosa	47085D-5	5
Salmonella enterica	BAA-1585D-5	5
Salmonella enterica	BAA-1674D-5	5

Supplementary Figure 22. LoD for bacterial cell line DNA. Genomic DNA (gDNA) derived from 13 different strains across 8 bacteria species were purchased from ATCC and individually assessed in 3 parallel experiments.

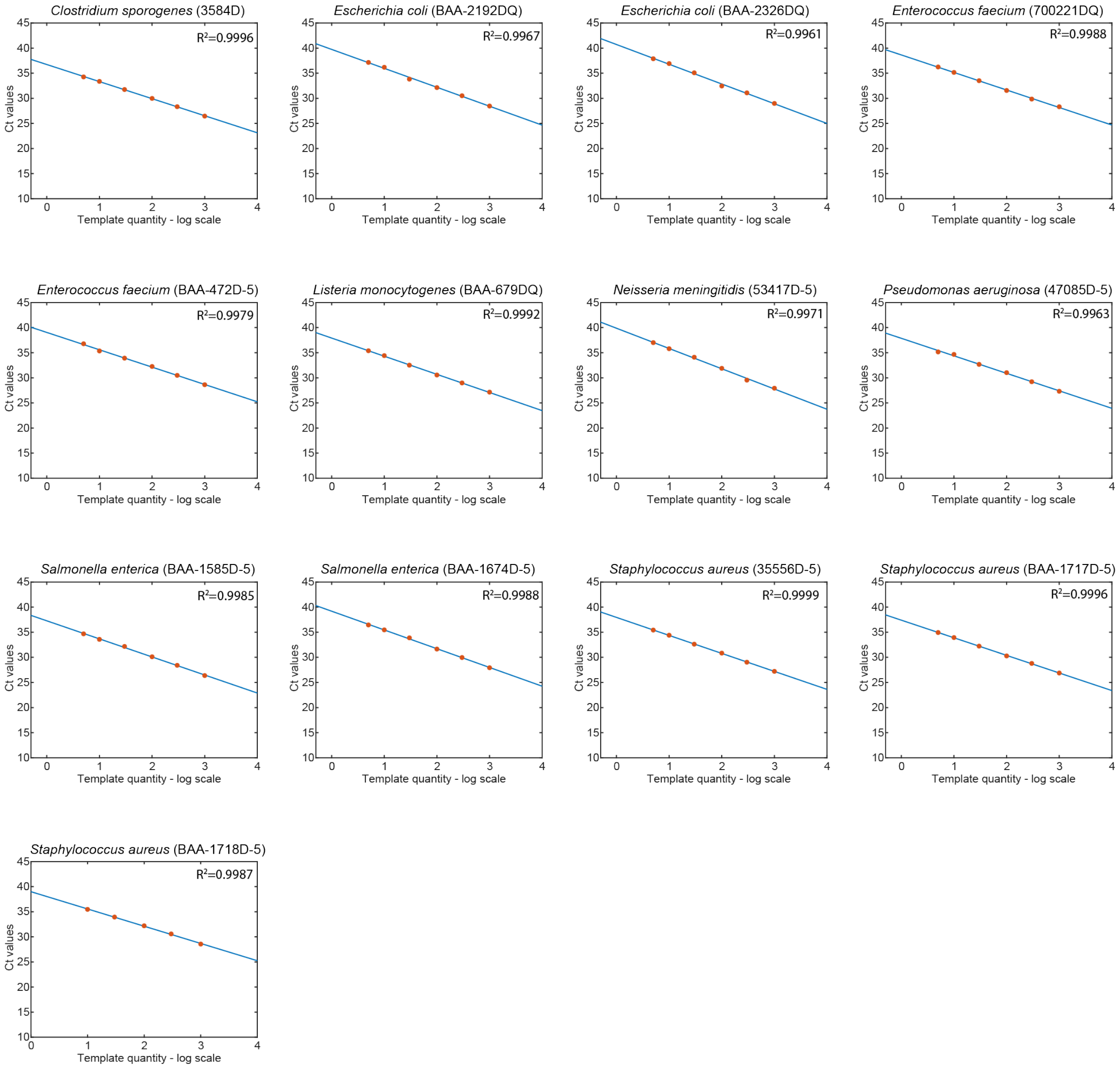

Supplementary Figure 23. Quantitative detection of bacterial cell-line genomic DNA by CCMA using the first Ct value. For each target species, linear fitting results of the logarithm of the input quantities (copies) of extracted gDNA and the Ct value of the first fluorescence are shown.

We investigated whether off-target amplification had any effect on LoD, as the LoD for each bacterial species can vary. To do so we compared the log-LoD of each bacterial sequence with its median nucleotide mismatches to off-target sequences (Supplementary Figure 24). No correlation between the two factors were found, suggesting that off-target amplifications do not affect LoDs of CCMA.

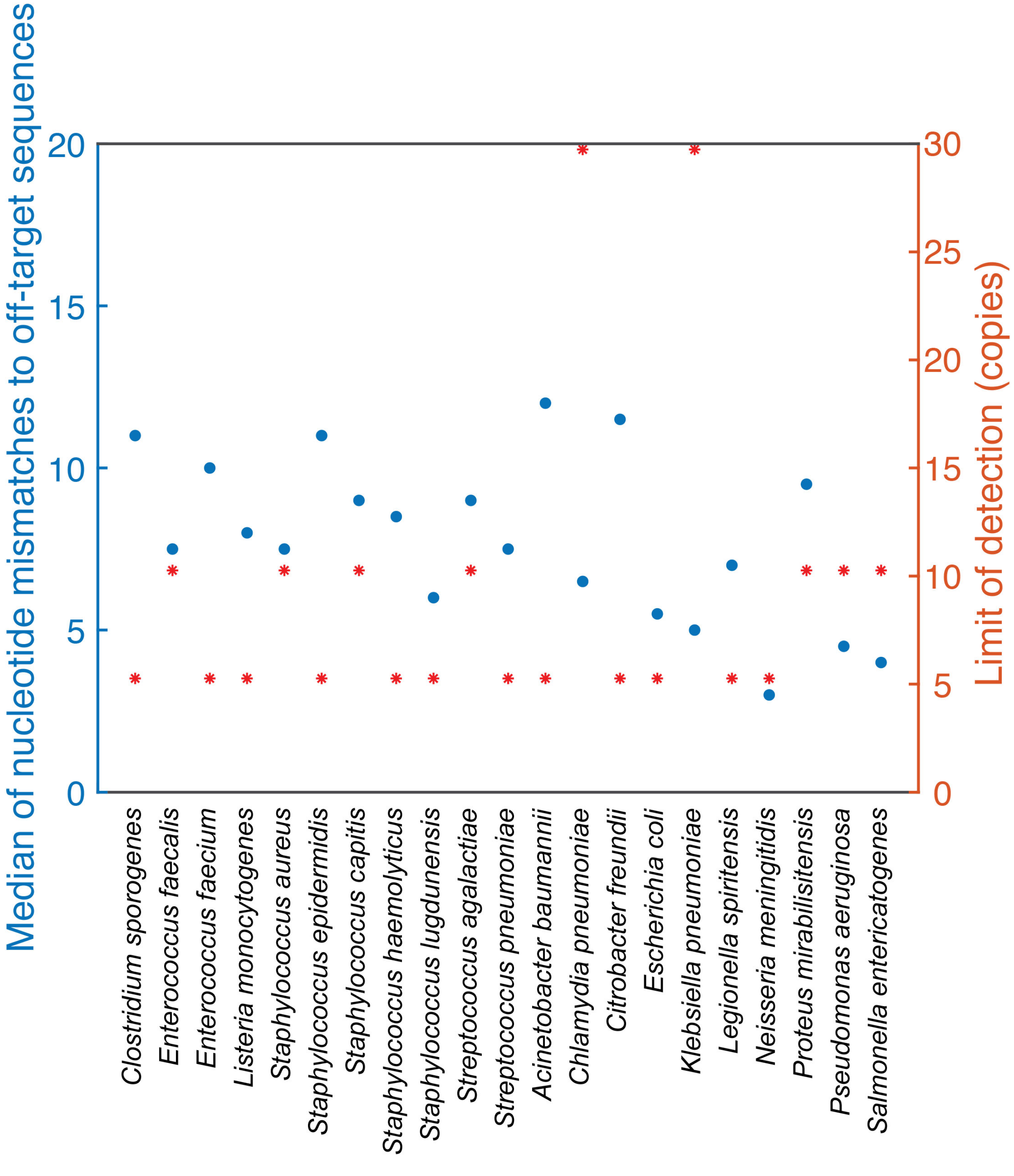

Supplementary Figure 24. Relationship between the limit of detection for each species and the median number of nucleotide mismatches to off-target sequences of 3 sets of primers of each species.

Section 10 CCMA applied to multiple-target-present situation

Color cycle multiplex amplification assumes that only a single target exists in a detection sample. In practice, simultaneous infection of two pathogens within one sample sometimes occurs. By using a two-tube detection strategy, color cycle multiplex amplification can theoretically achieve the successful determination of the existence of one target or two targets within a total of 20 targets.

Target	Color code	
	Tube 1	Tube 2
1(α)	A>B	B>C
2(γ)	A>C	A>D
3	A>D	A>C
4	A>E	B>A
5	B>A	A>E
6(β)	B>C	D>C
7	B>D	B>E
8	B>E	C>D
9	C>A	C>B
10	C>B	C>A
11	C>D	C>E
12	C>E	D>E
13	D>A	D>B
14	D>B	E>C
15	D>C	B>D
16	D>E	E>A
17	E>A	E>B
18	E>B	D>A
19	E>C	E>D
20	E>D	A>B

Supplementary Figure 25. Color code for a two-tube color cycle multiplex amplification detection strategy. ABCDE represent 5 distinct fluorescent colors. α, β and γ represent 3 demo targets tested in Supplementary Figure 26.

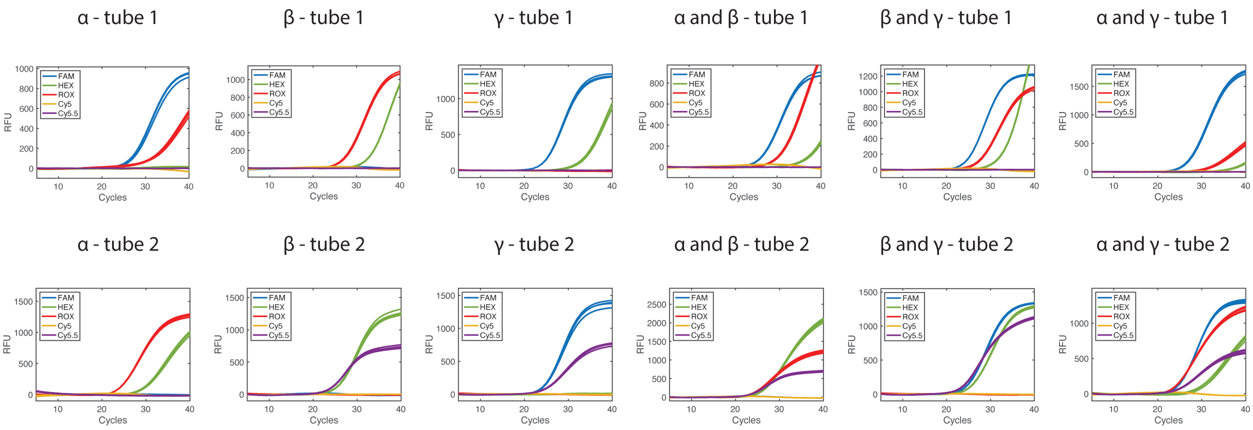

Supplementary Figure 26. Proof of concept: Identification of up to 2 co-existing species in the same sample via color cycle multiplex amplification with a 2-tube strategy. For tube 1, the color code for targets α, β and γ are Blue>Red, Red>Green and Blue>Green, respectively. The co-existence of α/β or β/γ or α/γ will all lead to a Blue>Red>Green order of fluorescence readouts. However, the color codes for these situations featuring co-existing species can be distinguished from each other. Thus, we can easily identify the presence of up to 2 co-existing species in the same sample via color cycle multiplex amplification using this kind of 2-tube strategy.

Section 11 CCMA applied to clinical samples

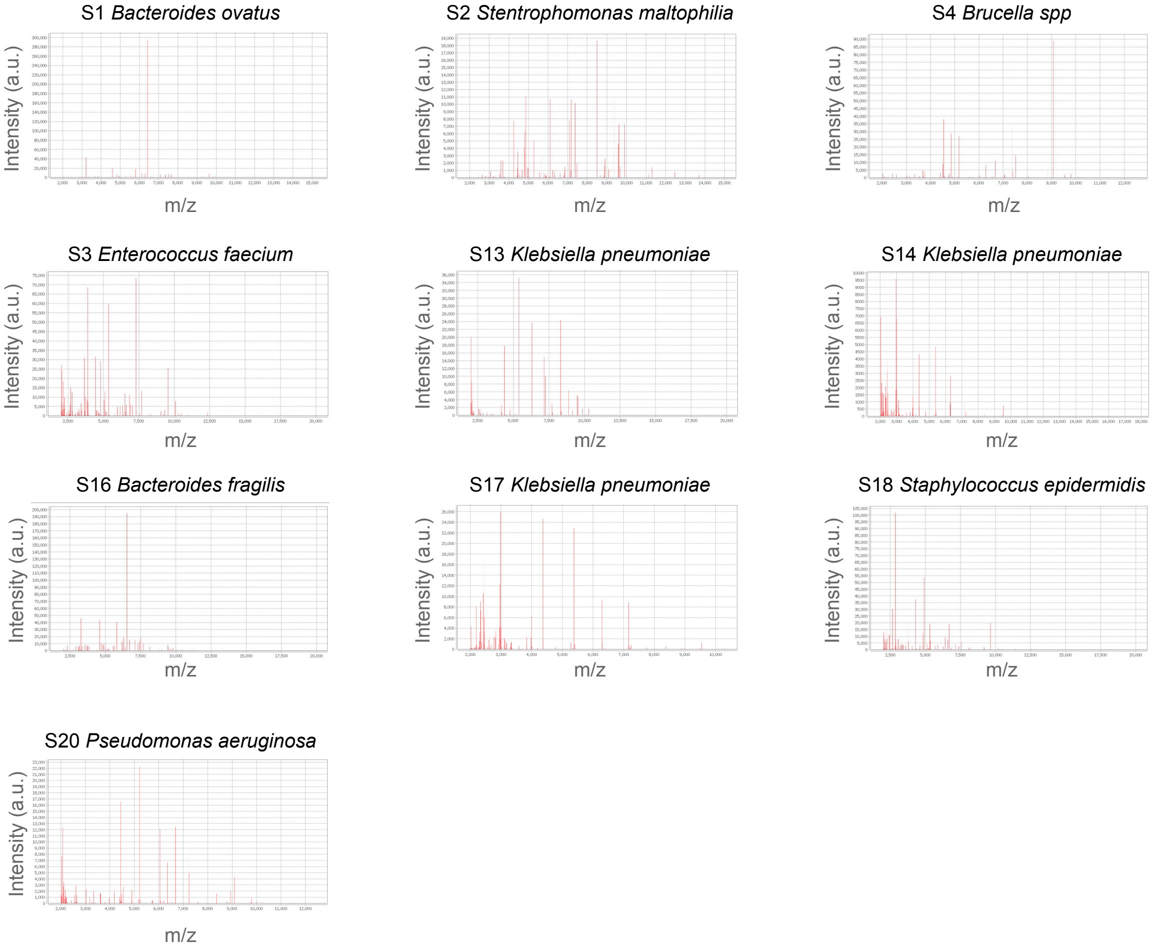

Supplementary Figure 27. Mass spectrum and identification results of clinical samples via Biomérieux VITEK MS microbial identification system.

Sample ID	Sample Type	Color cycle multiplex amplification	BIOMÉRIEUX microbial identification
S1	Blood	Not Detected	*Bacteroides ovatus*
S2	Blood	Not Detected	*Stenomonas maltophilia*
S3	Blood	*Enterococcus faecium*	*Enterococcus faecium*
S4	Blood	Not Detected	*Brucella spp*
S5	Blood	Not Detected	Not Detected
S6	Blood	Not Detected	*Bacteroides fragilis*
S7	Blood	Not Detected	*Streptococcus sanguinis*
S8	Blood	Not Detected	Not Detected
S9	Blood culture	Not Detected	Not Detected
S10	Blood culture	Not Detected	Not Detected
S11	Blood culture	Not Detected	Not Detected
S12	Blood culture	Not Detected	Not Detected
S13	Sputum	*Klebsiella pneumoniae*	*Klebsiella pneumoniae*
S14	Sputum	*Klebsiella pneumoniae*	*Klebsiella pneumoniae*
S15	Blood culture	Not Detected	*Bacteroides fragilis*
S16	Blood	Not Detected	*Bacteroides fragilis*
S17	Blood	*Klebsiella pneumoniae*	*Klebsiella pneumoniae*
S18	Blood culture	*Staphylococcus epidermidis*	*Staphylococcus epidermidis*
S19	Pleural effusion	Not Detected	*Prevotella species*
S20	Sputum	*Pseudomonas aeruginosa*	*Pseudomonas aeruginosa*
S21	Sputum	Not Detected	*Candida albicans*
S22	Sputum	*Proteus mirabilis*	Not Detected
S23	Blood culture	*Klebsiella pneumoniae*	*Klebsiella pneumoniae*
S24	Blood	Not Detected	*Pseudomonas aeruginosa*
S25	Colony	*Enterococcus faecalis*	*Enterococcus faecalis*
S26	Pleural effusion	Not Detected	*Prevotella species*
S27	Sputum	*Enterococcus faecium*	Not Detected
S28	Sputum	*Enterococcus faecium*	Not Detected
S29	Blood culture	*Pseudomonas aeruginosa*	*Pseudomonas aeruginosa*
S30	Blood culture	*Streptococcus pneumoniae*	*Streptococcus pneumoniae*
S31	Blood	*Streptococcus pneumoniae*	*Streptococcus pneumoniae*
S32	Blood	Not Detected	*Escherichia Coli*
S33	Blood	Not Detected	*Enterococcus faecalis*
S34	BALF	Not Detected	Not Detected
S35	BALF	Not Detected	Not Detected
S36	BALF	Not Detected	Not Detected

S37	Pleural effusion	Not Detected	Not Detected
S38	Sputum	Not Detected	Not Detected
S39	Blood	Not Detected	Not Detected
S40	Blood	Not Detected	Not Detected
S41	Blood	*Enterococcus faecium*	*Enterococcus faecium*
S42	Sputum	*Klebsiella pneumoniae*	*Klebsiella pneumoniae*
S43	Sputum	Not Detected	Not Detected
S44	Blood	Not Detected	Not Detected
S45	Blood culture	Not Detected	Not Detected

Supplementary Figure 28. Comparison of clinical sample test results of Color cycle multiplex amplification and BIOMÉRIEUX microbial identification system.

Section 12 Supplementary Tables

Supplementary Table 1. Sequences used in PoC experiments

Name	Sequences (5' -3')
Taqman probe sequences	
PoC_aac3II_T1	HEX-GTCCGGTCGAAGGAGGAGCGGAGA-BHQ1
PoC_aac3II_T2	FAM-ACCGTGGGTTCGGCCTGCTGAATC-BHQ1
PoC_aac3II_T3	Cy5-GCATTGCACTACGCCGAGGCGGTT-BHQ2
PoC_aac3II_T4	ROX-CATCGAGAAGGTGTCGTGGGCTTTGC-BHQ2
Primer sequences	
PoC_aac3II_F1	GATGGTGCATGCCTCACTTA
PoC_aac3II_R1	TATCCCATCACAGTGCCAGT
PoC_aac3II_F2	CCGTTCGATCCCGCAAC
PoC_aac3II_R2	CAACCGCGACCATCGAT
PoC_aac3II_F3	TTGGGTGCGCCGCTAA
PoC_aac3II_R3	CTTTCCTTCGATAGCAAAGCAATC
PoC_aac3II_F4	TATAGCAAATGCTTACGTGAAGCT
PoC_aac3II_R4	TCAAGATAGGTGACGCCGAA
Blocker sequences	
PoC_aac3II_B11	GCCAGTCGGCCCAACCGAAATA
PoC_aac3II_B12	GCCAGTCGGCCCAACCAAAAT
PoC_aac3II_B21	CATCGATGCATCGGGGTGCGAAAAA
PoC_aac3II_B22	CATCGATGCATCGGGGTGCAAAAA
PoC_aac3II_B31	AAAGCAATCGAGAATGCCGTTTGAATCAATAA
PoC_aac3II_B32	AAAGCAATCGAGAATGCCGTTTGAATCGTTAAAA
PoC_aac3II_B33	AAAGCAATCGAGAATGCCGTTTGAATCGATAAA
PoC_aac3II_B34	AAAGCAATCGAGAATGCCGTTTGAAAAAAT
PoC_aac3II_B35	AAAGCAATCGAGAATGCCGTTTGATAAAA
PoC_aac3II_B36	AAAGCAATCGAGAATGCCGTTTGTTAAA
PoC_aac3II_B37	AAAGCAATCGAGAATGCCGTTTATTAA
PoC_aac3II_B38	AAAGCAATCGAGAATGCCGTTAATTA
PoC_aac3II_B41	GCCGAACGTCACGATGTCCTGCAAAAA
PoC_aac3II_B42	GCCGAACGTCACGATGTCCTGAAAAA
PoC_aac3II_B43	GCCGAACGTCACGATGTCCTAAAAA
PoC_aac3II_B44	GCCGAACGTCACGATGTCCAAAAA
PoC_aac3II_B45	GCCGAACGTCACGATGTCAAAAA
PoC_aac3II_B46	GCCGAACGTCACGATGTAAAAA

Supplementary Table 2. Primer sequences

Species Name	Forward Primer	Forward Primer Sequence	Reverse Primer	Reverse Primer Sequence
Acinetobacter baumannii	fP1_2	tcagaaccggctgaatcagg	rP1_2	tgatactcaacatctacttctgaacttagat
	fP1_3	ggagtcaacaaggcaataccatga	rP1_3	aatgaatgctacaggccagct
	fP1_4	tcccaacgactagaggagaagtta	rP1_4	cctttcttagtacaaggtcgaatacttt
Citrobacter freundii	fP3_1	agatcttcttacaattcaatgaaccatagat	rP3_1	gaaaactttagttacttatatcattcctcgc
	fP3_3	tttgttaatgaaagcggatgaaatagaatt	rP3_3	gatttaatgagtggttggggttgtatat
	fP3_4	agaatcgtgaaaagatgcaccatg	rP3_4	cttttctcaataaacaaaacgtagagctt
Escherichia coli	fP4_2	cgcgaataaagcagcagaagc	rP4_2	caaaatcgatgaggttatctgccat
	fP4_3	gcatggagtcagcggtcca	rP4_3	aacgtttccgatcagaatccct
	fP4_4	gtggcgcaggtaataccct	rP4_4	accaaatcactggtcagtccat
Klebsiella pneumoniae	fP5_1	ccccgccgggacgttt	rP5_1	cgcaataccgcgagaggtag
	fP5_2	gtggtgataaagtagaaggtggtgt	rP5_2	ggtttcagcgattccagctaac
	fP5_3	atggacgcttatacgctgatga	rP5_3	tttttgaccacacatcactaaccc
Pseudomonas aeruginosa	fP6_1	ccgccgctgtagatataactga	rP6_1	tcacccattcgggccca
	fP6_2	caagtcagtggttctcactgga	rP6_2	cggatgtatgtgttccataccct
	fP6_3	gcgacatggggtcagcct	rP6_3	gccttggcactccctcc
Staphylococcus aureus	fP8_1	gcaagcattgacgagtcctatacat	rP8_1	ggggcatcaacagctcca
	fP8_2	ctgaaatgaaaggatgtgttatatatttatccttag	rP8_2	tcttttttgccatcgtttactggatt
	fP8_4	gtttttatgaattaaaataggtgaaagtaagttgg	rP8_4	agacatattcactcatccacgtatca
Salmonella enterica	fP9_1	cagttgcaggatatcgggcat	rP9_1	agacgctgtcacccagga
	fP9_2	gaacgggttctgcggattg	rP9_2	cggctgaatgatggagatacgat
	fP9_4	ttatatgcattgatgcattatttttatgaatttttatg	rP9_4	agtgagtcagctcaacccag
Proteus mirabilis	fP10_1	tgccgcaagctgagagag	rP10_1	gggatgaagataaaatggcgacatta
	fP10_3	tgcattaggataagctgattttaacgaa	rP10_3	ggtagatgcattctacggatttgtt
	fP10_4	ccgttttcagcaactttgcg	rP10_4	gcaacaattgggggactattccat
Streptococcus pneumoniae	fP11_1	agataaagccatggggaatctagg	rP11_1	cagaccatgcacctactgtttttt
	fP11_3	agaatgctactagtaagacagaagca	rP11_3	ttgctacagttcctgatgagttttg
	fP11_4	cgttacaactgctgtaatgctact	rP11_4	cccacgtcgactgaagcc
Staphylococcus epidermidis	fP13_1	catatctattttttgtatgtcgataccaatgattat	rP13_1	ttaaaaagaataaacaacaccgcgac
	fP13_2	ctggtataagttggtctcactggtt	rP13_2	aattattgcatcgatataacctgggaaa
	fP13_4	gtgtgaaagcacgtgacactg	rP13_4	tgagcgtttaaacctattaatgcctt
Enterococcus faecalis	fP15_2	ggatgagaagggtaaaaagttagtcaat	rP15_2	tgaactgataaagccccggttt
	fP15_3	gataccacaggacaagcggtt	rP15_3	cgcttgctgcaatcgtgaa
	fP15_4	caaatagtcaagggaaaattattgcagag	rP15_4	ggaaagcccctttgtaattcacaa
Neisseria meningitidis	fP16_1	tttaaacgataattgaccttgagttaatacg	rP16_1	agttgcgcccgcgatt
	fP16_2	cgagctgaacgcggaact	rP16_2	tgtctggacacgcggttg
	fP16_3	gcttttgcacttgcctcagaa	rP16_3	ggcagctcccgaaccat
Clostridium sporogenes	fP17_2	tgcttagaagaatttatatggattttacagga	rP17_2	atgtctttgtaaggtcctctattcca
	fP17_3	caggaatttattttagcgggtgtactt	rP17_3	gtatcgccttttattacttttgagactttta
	fP17_4	ttccaccgctccattttgatatattag	rP17_4	gttgctaaataccttgatactgattttagtaaa
Legionella spiritensis	fP18_1	gcttattgctatcatcttgggttcac	rP18_1	attaaaagcggatccctgtcga
	fP18_2	tggatggtgatgtttgccga	rP18_2	acatgctccggcaccg

	fP18_3	aatttggcataggggtgagcta	rP18_3	gccgtttgaaaagatagtgaccga
Listeria monocytogenes	fP19_1	caacaactaccaagcgaactttgat	rP19_1	caaagtaatatcgttcgcaggca
	fP19_2	actacagaactagcaacagctaaact	rP19_2	gattaaaccaaaaacccagcactct
	fP19_3	aatgcgggagtccaactagg	rP19_3	acttctgatggggaaatgattaacg
Staphylococcu s capitis	fP20_1	ctaggaaacctaaatcttttaagtgtctagtat	rP20_1	gcttacaaaaattacatcaaatgaatccga
	fP20_2	agttggcatttttgccatatctatctaaa	rP20_2	atgatgaaacggtaaatcgtactacaaa
	fP20_4	cattcaatcatatttcaaaggtattaagttaataatgat	rP20_4	ctatcagagtatcaagcacaacattctag
Streptococcus agalactiae	fP21_1	tcgtgatttcctccttcaagctaa	rP21_1	aacctttaatggcgagccct
	fP21_3	agaatcataaccgtattggcggta	rP21_3	ctctactatatctttaacagcgagtcca
	fP21_4	ggaaaggtattgttttaattttatctagcatagt	rP21_4	gccattaccttgtggtaaaaatacatatttt
Chlamydia pneumoniae	fP22_1	ctagttctcaatccccagcagaa	rP22_1	caggtagatctgagcaagtgtattca
	fP22_2	cctggcgttacccacaact	rP22_2	ggattccatggcgtagacatcg
	fP22_3	tgatgatgaaatgcaacaagcaga	rP22_3	gtacgaaacttaaggcaagctactc
Enterococcus faecium	fP26_1	ggttggcgctaatttaggtacttt	rP26_1	tcttatcactgtctttctattcgtcct
	fP26_2	gccatcggaacgtcaagtttaaga	rP26_2	aaagatacgaatttttgctttaccagc
	fP26_3	cgagcagtgaatggctgtg	rP26_3	cgagctttcatttttcttgtcattcctat
Staphylococcu s haemolyticus	fP27_1	attttctcgtggtacagatagtggt	rP27_1	tgtcagtaacgtatttccacgctt
	fP27_2	cctaaaagagtttggcgtagacttt	rP27_2	tgttatcaagatacttaataaatcgaccgtc
	fP27_3	gttttgggaaacatgttatcgtaagag	rP27_3	aatgacctgtcattgagccga
Staphylococcu s lugdunensis	fP28_1	cctatgacgcgtgatgaagca	rP28_1	gtaatactgccacaacaggttctttaa
	fP28_2	tttattgaagcagcatcaacagca	rP28_2	gcccaacattcaccagtagtatga
	fP28_4	atcaagcacggccagtcaa	rP28_4	gactgcctttgttaattggccat

Supplementary Table 3. TaqMan Probe sequences

Species Name	Probe	Sequence
Acinetobacter baumannii	S1_T1_HEX	/5HEX/ACAAGCTGA/ZEN/GGATGAGGTGGTTGCAGG/3IABkFQ/
	S1_T2_FAM	/56-FAM/TGCTGCATG/ZEN/GGACTCAGACCGTTACC/3IABkFQ/
	S1_T3_CY5	/5Cy5/TAGCTATGC/TAO/AATACCAGCTAAAGTTGTTCCCC/3IAbRQSp/
Citrobacter freundii	S3_T1_ROX	/56-ROXN/TTCCAGTTCAATGATCTGTCTGTTCAGTGTGC/3IAbRQSp/
	S3_T2_FAM	/56-FAM/TAGACAACT/ZEN/GGAATCACAAGGTGTTTTTGCTG/3IABkFQ/
	S3_T3_HEX	/5HEX/TTTGATAGC/ZEN/GAAAAGTGCTTTGCAGCAGG/3IABkFQ/
Escherichia coli	S4_T1_FAM	/56-FAM/TGCAGTTCC/ZEN/AGGTTTAGATTCAGAGGCTG/3IABkFQ/
	S4_T2_HEX	/5HEX/AGCATGGAT/ZEN/TGATGTTGCTCCCCCAAG/3IABkFQ/
	S4_T3_ROX	/56-ROXN/ACCACAGATTGTGTTTGGCGAAGGTGC/3IAbRQSp/
Klebsiella pneumoniae	S5_T1_CY5	/5Cy5/CGATCTGAA/TAO/TGAAATCGGCATCACTGAGC/3IAbRQSp/
	S5_T2_ROX	/56-ROXN/CGGTGGTATAGCGAATATGCAGGCGAA/3IAbRQSp/
	S5_T3_FAM	/56-FAM/GATTCCGGG/ZEN/TTAGAGTTTGGTGGCAGC/3IABkFQ/
Pseudomonas aeruginosa	S6_T1_ROX	/56-ROXN/AGAAGCTGCCACGCCCGGCTTTG/3IAbRQSp/
	S6_T2_HEX	/5HEX/TTTCCATAT/ZEN/GCGGTCACATCAGCTGCA/3IABkFQ/
	S6_T3_FAM	/56-FAM/ACATTGCAG/ZEN/TAGGCATGGCACTTGACG/3IABkFQ/
Staphylococcus aureus	S8_T1_FAM	/56-FAM/CGTCATTCC/ZEN/AAATACGAGGTTACCGAACG/3IABkFQ/
	S8_T2_CY5	/5Cy5/AGTGCAGAT/TAO/TCAGGGTTGAAAATGACAGC/3IAbRQSp/
	S8_T4_CY5.5	/5Cy55/TGGAGCGTTGTGATTTTTATGAGAAATTGTATGAG/3BHQ_2/
Salmonella enterica	S9_T1_FAM	/56-FAM/ATCGCCAGC/ZEN/CAGTGCATTTCACCC/3IABkFQ/
	S9_T2_CY5	/5Cy5/AGTCCAGAT/TAO/CCAGGTACTCAGCAACGG/3IAbRQSp/
	S9_T3_HEX	/5HEX/AACTGGCCG/ZEN/GCCCACTATTCCGG/3IABkFQ/
Proteus mirabilis	S10_T1_CY5	/5Cy5/TCATCTCAA/TAO/GCTCTTTCGCTTCTGGCC/3IAbRQSp/
	S10_T2_FAM	/56-FAM/CAGGTGTCG/ZEN/TTAATAACATATCTCCATGATGTTGAA/3IABkFQ/
	S10_T3_ROX	/56-ROXN/CCCGTTTGGCTAACCAACTATGAAATGGC/3IAbRQSp/
Streptococcus pneumoniae	S11_T1_FAM	/56-FAM/CCTTGATGT/ZEN/TTCCTGTTGCTGAACGGC/3IABkFQ/
	S11_T2_CY5	/5Cy5/GCTTCTGAA/TAO/GTTTTCGGAAATAGAGCCGC/3IAbRQSp/
	S11_T3_ROX	/56-ROXN/CAATAGTTGCTGCTATTGCCGCTATTACACTC/3IAbRQSp/
Staphylococcus epidermidis	S13_T1_HEX	/5HEX/ATGTATTTG/ZEN/CGTGGCCGGGTTTAGGAC/3IABkFQ/
	S13_T2_FAM	/56-FAM/AGGTCGAGA/ZEN/TGTATTAACACGGATAATATACGCC/3IABkFQ/
	S13_T3_ROX	/56-ROXN/AGGACACATGGTCAGATGAGACGCTCG/3IAbRQSp/
Enterococcus faecalis	S15_T1_CY5	/5Cy5/AAATTAGAG/TAO/CATGCCGTAACCACGCCG/3IAbRQSp/
	S15_T2_FAM	/56-FAM/TGCAAACGG/ZEN/AAAAGTCACAGTGACGGA/3IABkFQ/
	S15_T3_ROX	/56-ROXN/CCACGCCAGTCCCATTTGAAATTGCTG/3IAbRQSp/
Neisseria meningitidis	S16_T1_HEX	/5HEX/TGGCAAACG/ZEN/GGAACATGAAACTGTCGT/3IABkFQ/
	S16_T2_CY5	/5Cy5/GGCACGGGA/TAO/TTTGCGGACAAGATGG/3IAbRQSp/
	S16_T3_FAM	/56-FAM/AAAACCCTG/ZEN/AAAGATGGCGGCATGAGC/3IABkFQ/
Clostridium sporogenes	S17_T1_HEX	/5HEX/ACCTATTGA/ZEN/TGAGGAAGAAACTATTGCAAACCCA/3IABkFQ/
	S17_T2_ROX	/56-ROXN/TGGAAAGACTCCACAGGAAGCCATCGA/3IAbRQSp/
	S17_T3_CY5	/5Cy5/ACATAAGCC/TAO/GGTTTTCCCATGGTACCG/3IAbRQSp/
Legionella spiritensis	S18_T1_ROX	/56-ROXN/ACTCTGACGTCTGCCCCTGGCTG/3IAbRQSp/
	S18_T2_CY5	/5Cy5/TGGAGTTCA/TAO/GTATAGGTTATGGACGCGGT/3IAbRQSp/
	S18_T3_HEX	/5HEX/GACTCAGTG/ZEN/TGACGGCTTCATCATCGC/3IABkFQ/

Listeria monocytogenes	S19_T1_ROX	/56-ROXN/CCTGAACCAACATCACCGTCCAAAACG/3IAbRQSp/
	S19_T2_CY5	/5Cy5/AATGCCCCG/TAO/TGGAAAACATTGTTTGCC/3IAbRQSp/
	S19_T3_HEX	/5HEX/AAAATGATG/ZEN/CGAGCGCGACCTTCAACT/3IABkFQ/
Staphylococcus capitis	S20_T1_CY5	/5Cy5/CAAGTCCGT/TAO/GTATTCTGGACCGACACC/3IAbRQSp/
	S20_T2_ROX	/56-ROXN/AGGATTTGCTTTGTTAAACCAACTTCCAGCAT/3IAbRQSp/
	S20_T3_HEX	/5HEX/CGGCTCAAC/ZEN/AGGACAAGATTCCGTTAAAC/3IABkFQ/
Streptococcus agalactiae	S21_T1_CY5	/5Cy5/ATTACCGTT/TAO/TTAGAAGCCGATTTGTCGAGTTC/3IAbRQSp/
	S21_T2_HEX	/5HEX/TGCGAATGG/ZEN/CTTAGTATGGAGAAAGACACTAC/3IABkFQ/
	S21_T3_FAM	/56-FAM/ACAAATTCA/ZEN/CTTAATCCTGCATTTTGCCATGC/3IABkFQ/
Chlamydia pneumoniae	S22_T1_CY5	/5Cy5/TTGCCTCTA/TAO/GATTGATGCATGAAGAAAGAGGT/3IAbRQSp/
	S22_T2_ROX	/56-ROXN/AGCTACTTATTTTGTCATACCCTCGATCAGTAGAG/3IAbRQSp/
	S22_T3_FAM	/56-FAM/TGGGATGCT/ZEN/TACTCTATCGGCTTAGTTATAAACAC/3IABkFQ/
Enterococcus faecium	S26_T1_ROX	/56-ROXN/AAACACTTGCTGTTTTCGTCAATGGCAGT/3IAbRQSp/
	S26_T2_HEX	/5HEX/CAACCTTCC/ZEN/ATGTTTAAAGTGACGATCCAAGG/3IABkFQ/
	S26_T3_CY5.5	/5Cy55/TCGAATCTATCACTCTATGTTATGGAGAGTCCGA/3BHQ_2/
Staphylococcus haemolyticus	S27_T1_FAM	/56-FAM/ACGTATGCT/ZEN/AAGAGAAGCTAAGAAACGCAAGA/3IABkFQ/
	S27_T2_HEX	/5HEX/TGCTTTGGG/ZEN/ACGCAATGTGGAAACAAC/3IABkFQ/
	S27_T3_CY5.5	/5Cy55/TGGACAACGTGTTAAACCTGGTGACGC/3BHQ_2/
Staphylococcus lugdunensis	S28_T1_ROX	/56-ROXN/AAAGATGCTATTGTAGCGCGGGAAGCC/3IAbRQSp/
	S28_T2_FAM	/56-FAM/ACTATAAAG/ZEN/GAGGCGTCAGGACATGGTGT/3IABkFQ/
	S28_T3_CY5.5	/5Cy55/ATGGGAAATTGATGGCACGTTACCATATGAGA/3BHQ_2/

Supplementary Table 4. Blocker sequences

Species Name	Blocker name	Blocker Sequence	ΔGrxn (kcal/mol)
Acinetobacter baumannii	BLK_S1T1	TCTGAACTTAGATGACCAATACTTGTGTCTAAAAAA	-1.34
	BLK_S1T2	CCAGCTTTAATTAGTGACCAAAACATGGAAATAT	-1.19
Citrobacter freundii	BLK_S3T3	GTAGAGCTTACGTTAGTTGATAAAGATATAAGTGAAAAGATAAAAA	-3.50
Escherichia coli	BLK_S4T2	AGAATCCCTGTCCCACCATGTTTAAAAAT	-1.50
	BLK_S4T1	TATCTGCCATTTCATCGCTAAAAAAAGAGAAATGTAATTA	-3.01
Klebsiella pneumoniae	BLK_S5T1	GAGAGGTAGTAGACAATGTCATCTGAGAGATGTAAAAAA	-3.35
	BLK_S5T3	CACTAACCCGTTGCGGTAGTAAATAACAAAAAAAAT	-3.06
Pseudomonas aeruginosa	BLK_S6T1	GGCCCAGACCGGACGCAATAAA	-1.01
	BLK_S6T2	CCATACCCTGAAAAACTCAGAGTGGCGAAAAAA	-3.71
Staphylococcus aureus	BLK_S8T1	AGCTCCAGGAAACTTTAAAGATGGTACAGATTATAAA	-3.12
	BLK_S8T2	GTTTACTGGATTAGTTTAAATCCATCGAAAATCGTTGTATTAA	-3.56
Salmonella enterica	BLK_S9T2	GAGATACGATTGAATGGGGTCTGTCATCCTGGAAAAAA	-5.68
	BLK_S9T3	AACCCAGGGATTAGAGCGTAGTTTAATTTT	-1.14
Streptococcus pneumoniae	BLK_S11T2	GATGAGTTTTGTGCTGCTTCAGCTAAATCAAATTTT	-3.44
Staphylococcus epidermidis	BLK_S13T1	CGCGACAATGAGCACGTATGCTTGTTAATA	-3.38
Enterococcus faecalis	BLK_S15T3	TTGTAATTCACAAAGTTGTCACTAGCAACAACGTAAAAA	-4.01
Neisseria meningitidis	BLK_S16T1	CGCGATTTCGCGGTACGATCATTAAA	-1.31
	BLK_S16T3	CGAACCATATCCCGCACCTCGTTTAAA	-2.01
Clostridium sporogenes	BLK_S17T2	TTTGAGACTTTTAATTTATTAATAAAACCCTCTTGCCAAAAATA	-3.09
Legionella spiritensis	BLK_S18T1	CTGTCGATTGATAAACATCGATACCTGCAAGTAAAAA	-3.51
	BLK_S18T3	ATAGTGACCGACGTCGAGCATGATAAAATA	-1.14
Listeria monocytogenes	BLK_S19T1	CAGGCATTTTCATCGTTTTAAAATCCCATTTTGAAAAAA	-3.10
	BLK_S19T2	AGCACTCTCCATTTTAGAAAATGCTGGAAAAAA	-1.25
Streptococcus agalactiae	BLK_S21T3	GGTAAAAATACATATTTTCAATACCTTGAAAGTAACTTTCAAGTAT CCATTTAA	-5.89
Chlamydia pneumoniae	BLK_S22T1	CAAGTGTATTCAGCCGTACGAGAACATACAATAAAA	-3.24
	BLK_S22T2	GTAGACATCGGAAAGGCCAGTGGTAATAA	-1.06
Enterococcus faecium	BLK_S26T1	TATTCGTCCTGTGTACTACTAGAAGGCATTATT	-1.30
	BLK_S26T2	TTTACCAGCTGGACAAGTCAGCCATTTTCCTATAAA	-5.28
Staphylococcus haemolyticus	BLK_S27T2	GACCGTCGCCATCGCCACCAATTTAA	-3.44
Staphylococcus lugdunensis	BLK_S28T1	CAGGTTCTTTAACTGCAGCTGACTTGGCTAAAAA	-4.29

Supplementary Table 5. gBlock sequences

Species Name / Sequence
Acinetobacter baumannii<br>TTACCGAAAAAAGATGGCTGAGTTCCATCCGGATAAATACCAGACCTTACCTGAATCAGTACGGCAGCTTATTGAGCAACAGGCACAGCAATTGAATGAAGCGAGAGCTGTATTAAAGAGATATCTGGACAAT<br>AACTAAGAATAAAGAATTTTCAAATACATATTACCTCAATGAAGGCAGCATCTAATGCGCCGTATATCTTCTATTTCAAGGTAACAATATGAATATGATAAAGAACAATGTTAATTCAGCCCCAAAAATACCG<br>CCATTATATTGGCTGTTAACTATAGTCATAATGGTAGTGTTAATTGCAGGTGGAATTTTATTTAACCAACATCAAAAACGACAAACTGAACTTCAGTTAGCTCAACTGGCTTTAGAGCAATCAAAAGTGCGTG<br>TTCAAACCTCTAGTACTCAAGCAGCATCAGAACCGGCTGAATCAGGTTTCACTGGAATTGAAAGTATGGTCGAGGACACTGAATCTAAAGCTACTAATTTACAAGCTGAGGATGAGGTGGTTGCAGGTGCAAT<br>AGCCGCAGTTGATCAGGCAAAAGCACAAGACACAAGTATTGGTCATCTAAGTTCAGAAGTAGATGTTGAGTATCATGAAGGAAAAACCTTAAGTTCCAATGCTTCCCGTTACGGATTAAAACCAATCAAATCT<br>GCTTCAGCCGATATAAAAGCAACCATTCCTACCATTGATCAAATGCTAGTGTCGCATCTCATGAATGTGTGGGGCCGTTCAAGTTCAGATCAGGTGGCTAAACGAGTTGAAGGTTGGAGTCAACAAGGCAATA<br>CCATGACTTACCGTGCTGCATGGGACTCAGACCGTTACCAATGCACCTGGTATGTGGTGAGCTGGGATAAGAACAGCAATCAAGTCCTCAAAGAGGGATATCAGCCATGTTTTGGTCACTAATTAAAGCTGGC<br>CTGTAGCATTCATTTAATTATCACTTTACTTAATCAGAGAAAATATCATGAACTCAATCAAACTTATCGCATGTGGAATTTTAAGCCTTTCAGTCAGTTCTATTGCCTTTGCTAAAACAGAGCAGATTACACT<br>TAAAGCCAATGTCTATTACGGAGAAGAATCAGTTGTATTCCCAACGACTAGAGGAGAAGTTATTTTAAATAGCTATGCAATACCAGCTAAAGTTGTTCCCCATATTAAACCTTATAAGAAAGGGCAATGTTTA<br>GAAATCAAATCTAAACATGGTTTCTTCAAAGATACTGGTGATGGGCAATATATTGAAAGTATTCGACCTTGTACTAAGAAAGGTTTAGCGACTCCAAAAGTAAATCGTTAAGTTAAAAAACGTCATAAACCTA<br>ATCACACCA
Citrobacter freundii<br>TGTGTTTCTTCAGTCATACTAAGATAAACCTTATCTATCAAAATCCGCTTGGGAAGATCTTCTTACAATTCAATGAACCATAGATCATTTGCTTTAGATTTTTTCTCCGTATTCCAGTTCAATGATCTGTCTG<br>TTCAGTGTGCTTCTGAAAGACATCACTAAAATCGTCTAAAACAATAACATCATGCCTTGCACTCCAAAAATGCTATGATCATGCGAGGAATGATATAAGTAACTAAAGTTTTCTAATTACCTTCCCAATATAT<br>TAACTCTTTACTCCAAAGGTAGTATTTAATTCGCTGCCACTAAATCGTGCCGGTGAATAATATGTCTTCTGTACTGCGCAAATATTAGCTTTATCAAATACACCTGCAATTACATGTGCTGGCAAAGGTTCAG<br>AGATATCATTCATTAATGTAATGCACTCTTTATTATCTTTAATCTCTTCATATCCTATCATCGTATGTTTTGACCCATAGACATGCAGTCCATAAGACAATGCTTCTGCAACTTTTGTTTTCATTCCACTACC<br>TAACATTAAGGGAGAATAAAAAACATCACATGAGGATAAGAGCACTGACATTTCATCATTACTTAAGTCTTCAAACATATATACATTTTCATTTTTTCCGAGTGACAGACTTGATAACTTCCTTCCAGCTAGA<br>ACAAAAACCACATTATTATTATCTTTTGCTTTCTCTATTATTTTATATGCAGCCTCAACATTTGGAAAAAAATCAAAAGATGCAGTAAAAACCAACTTACGTCTGGAGTCAGCATTTAATATATTAATTTTGT<br>TAATGAAAGCGGATGAAATAGAATTCTTAGACTGTGTATTTTCATAGACAACTGGAATCACAAGGTGTTTTTGCTGACAATTTTTATAATAATCATTCATTAATTCTCTGTCATTCTCGGTTATATACGTCAC<br>CAGATCAGAAATATACAACCCCAACCACTCATTAAATCTAATAATTAATTTTTCATAGATACTAGTTACCGTTGCCTTTTTTTGACTGATTAAGTTAAATTCAAAGTTATCAGTAAATAAAATAATATTCTTT<br>TTGGGAAAGAATAACTTAATAAGATAGCAAATAATTCCCAATCTGGAGTTGTGAATACCAATAATATCATTATCTCGACAAATGCGAAGAATCGTGAAAAGATGCACCATGTAAAAAGAAGGCAATAGCAAAG<br>CTCGTGAAATTACATCTTGCAATAACCCTTTTGATAGCGAAAAGTGCTTTGCAGCAGGAACATCAAAATTCTTTTCACTTATATCTTTATCAACTAACGTAAGCTCTACGTTTTGTTTATTGAGAAAAGATAC<br>TAGCGTTCTTAAGTACACCCCTCCCCCAGTTAATTCATTGTAAGAATTTATAGATATCAA
Escherichia coli<br>TTAGTATTTATGCAAGCGATATAAGTAGTCACCGCTTAGACTATTGACATTCATGATGCCTGTTAATTCAAATTTCAGGTATGATCAGTCATTTATTGTTGTGATACTGATTCCGCTAGTTTAGCATAACTGT<br>CCTGCAAATCATTGTTCAAAAAAGAAAGCTCCTACAAACTGTATCCTGAATCTCTACCAGAAGATAATATACTTGGTTATTTTTGTGCATAAGCTCACATAAAAATAATATTAATTATGTTTTCTTAGAGATA<br>GAAAAATAAACAGGTAAGCAGAAGACGGACTTTTTTAATGCGAAAACGTATTCTTTTTATTGGCCCACCGCTGTACGGTTTGTTATACCCATTGATTTCTCTGGCTCAGGCCTTTCGTGTAATTGGACATGAT<br>GTAGTAATTAGTAGCGCTGGCAAATTCGCGAATAAAGCAGCAGAAGCTGGACTGGTTGTTTTTGATGCAGTTCCAGGTTTAGATTCAGAGGCTGGATATCGCCATCAGGAAGAGTTGAGGAAAAAAAGTAATA<br>TTATTGGTCATTTCTCTTTTTTTAGCGATGAAATGGCAGATAACCTCATCGATTTTGCAGGAAAATGGAGGCCAGATTTAATAGTCTATCCCCCTCTTGATCCGGCAGGCCCATTGGTTGCTGCTAAATATAG<br>AATTCCTTCAGTGATGCTGGCTGTTGGATTCGCGCATACATCTGCCCATATTCAGATGTTAAACCGTTCTTTAAGCAATGCTTACAGGCGGCATGGAGTCAGCGGTCCACTATGTGATTTAGCATGGATTGAT<br>GTTGCTCCCCCAAGTATGAGCATTCTTAAAAATGCTGGAGAACCGGTTATCTCAATGAGATATATTCCTTATAACGGAGGTGCTGTAAAGGAAACATGGTGGGACAGGGATTCTGATCGGAAACGTTTACTTA<br>TCAGCCTTGGCACTGTAAAACCAATGGTTGATGGTCTGGAGCTGATTTCATGGGTTATGGATTCTGCAAATGAAGTTGATGCTGATATCATTTTGCAACTTGCAATAAATGCTCGTACTGGTTTACGAAAACT<br>ACCATCAAATGTACGTCTGGTTGACTGGATACCTATGGGTGTATTCCTTAATGGAGCTGATGGATTTATTCATCACGGTGGCGCAGGTAATACCCTGACAGCGCTGTATAGTGGAATACCACAGATTGTGTTT<br>GGCGAAGGTGCAGATCGCTCTGTTAATGCAGAAATTGTTGCGAAGCGTGGGTGTGGGATTATTCCGGACAAGCATGGACTGACCAGTGATTTGGTAAATCGCCTTCTTTATGATGATTCACTACGCTTCTGTT<br>CAGATCAGGTAGCCGCTGAAATGGCTGAACAAC
Klebsiella pneumoniae<br>CGGTGGCGGCAGTTAAGCGGGTGATCGAGGGGCTGGGGTATTCGATGACCATTGAGGAATGGTGGGAGGTGGCCGACCCCGCCGGGACGTTTCGGCTTGAGATCGATCTGAATGAAATCGGCATCACTGAGCC<br>GATGATTTATGAGCTTGAGAGGATTATAGGTGATGCCAAACCGGCGAGCAGACACATCTCTCAGATGACATTGTCTACTACCTCTCGCGGTATTGCGCATCTTGGTGCTGCCGCAATTATTGGCGACGAAGTA<br>AGTGTTTATCCGCAGCAATATAAGCCGGGCAACGATATTTTTTACGATGGCCTGATTTTCCATGACGGCAATTATCAGTATGAGGAATGATATGGCGAGCATAAAAGAATTGCCGCGCTGGGAAGATGAGGTC<br>TATCAGATCGCGCGTGGTGATAAAGTAGAAGGTGGTGTCGGTGGTATAGCGAATATGCAGGCGAAGACGCTGGCAGAACGTACCCGGTATCTAAAAAACGTTGTTGAATCTATTCCGGATTACCGCGAATTCA<br>CATTTTATAAAACTGAAAATGATCCGGAAGGAAAGTTAGCTGGAATCGCTGAAACCCATGACGGCCAGCTATTTCGTGTTGCTCAGGGGATCGATAGCGAAAACTCTTTTATCTATTATCGGAATGATGATGG<br>TGATGCTGTTCCGGTTGCATGGCAGCCTGGCACAGAGTTAGTCAAAATATTGAGCAACCTGATTAGTGATCAGGGAGATAACCCATTTTCTGTCGTTTTCGATAATGGGCTTTCTCCGTTGGGCTATAAAAAT<br>GGACGCTTATACGCTGATGAATTTGAAAAGCTATACTCCTCAGATTCCGGGTTAGAGTTTGGTGGCAGCATAATTGATAATAATCCTCCAGAAGGTTGGAGATTTGTTATTTACTACCGCAACGGGTTAGTGA<br>TGTGTGGTCAAAAAGATGACGGCACAATGATCGGTTTTGGTGAGGGGGGGAGCGGTGGCGGTTCTATTGAACCAGGTGATACAGCTCCTGACTATGATTCAATTCGAATGTATTCAGGTACAGCGACAGTGCG<br>TGACGTAGTTGGGAAACGCGCTGGCGGTCGTTTTGTTGTTAATCCGGATGACACCACATCGGAGGAGATCCCCGGAGGGATACTGGTCGATGTGCTGGGGCGTCGATGGTATCGACAGGCTGATTTTGTTTCC<br>TATGATATGTTTCTTGCGCCGCGAATACCCGCAGCGACTCTGTTGTCCGTACAGGTTGCACTGGCAATGGGGAACAGGTCATCAGCAATAGCATACCTCGCAGGAGTGGAGGCAGCAGATAAAGCTATCCAGG<br>CGGCTCACCGCTTTGCGAACATCATGAAAATCCCCGTACGTCAGAATGACGGTGCATTCCTGATGTTGGTTGATCATGAAGCAGAAGTACGAACAAATACCGACCTGTCAGGGGCGATAATTCTTACCTCTGC<br>AAATTCCGGTGTGAACGAGATACGCTGGGGACCTTTACGACTGCTTGACCCTTCAGCACCTGAACCGATGCGCATGTTCAACATAAAAGGTAAACCGCGTATTGAACTGACAGCTGAAGAGTTAGCGACATTT<br>AACGCAACGTATTCAAAGTATCTGAAAAAGGCTCTCAGTACCTGCCTTATCCGAAACTGTACGCGTATTACGGTGGGATGTTCTATGCGCTGTCGCATGAAGTGGAAATTTACCGCAATGGAGAAAGGAATA<br>AGCCGCGTGAC
Pseudomonas aeruginosa<br>CAGAGTTGAGATGGGGCTTTTACAGTCATGAGTATCTATGGATTACGTATCTATCGAGAGAATGGGACCGCCGCTGTAGATATAACTGACAGGGCGTTGCGGGTTGTATATTCCAGACGGGTTGATGCAGTTT<br>CGAAAGGAGAAGCTGCCACGCCCGGCTTTGGCCCGAACAATGCTTCCGTCTACGTGATTGGTGATGAGCACAATAAGCGTCCGGTCTGGGCCCGAATGGGTGATGGCGTTGTTCAATGGGGCTACGAGGATTG<br>GTGGCCATCACTATTCCATACTTCTGGAACCTTATATGTGGTGGCCAAGGTATGAGTGAGTATGGGGTCTTGGTAAGGAATGGCTTGGGGCAGACTGTAATAGATGGAGAGTTTCATAACCTTTCTCTTCTGT<br>TGGAAAGAAATATTGAAGTCGAGACAAGTCAGTGGTTCTCACTGGATTTTCCATATGCGGTCACATCAGCTGCACCTCCTGTTCTAGCAGTTCAAGCTTGGAAGAATAAAATTCTTTACTTCGACTCAGTACA<br>GTATCGAGGTGGGCCGGGAAACTGGACAGGCGCCACTCTGAGTTTTTCAGGGTATGGAACACATACATCCGGCTCCGTAAGAATTAGAGTTTATGCCTACGCATTGCCTTTGCTGAGAGGATATGGACTTCGT<br>GTTCGAAACTCGGCGGGAAGTGTCGTTTTCGATTCTCTAAGACTTCCCTTGGTTTTTAGTGCGGAACTGGGCGGGGCGCCAGAAGATTGGGTTAGGGTATCTGGGGAACCGATTATAGGAGCTGGCCGCATAG<br>ACATATATCGCCCAGCGACATGGGGTCAGCCTGCCGATAGCTACATTGCAGTAGGCATGGCACTTGACGTGGAATTTGGCAGAGTGGCGGTATCTGGTGGTACGGCTAATGCAATTATTTATATACGCTGGGG<br>TTTCACTGAGGAGGGAGTGCCAAGGCTTATGCAGCATGCATATACCGGGCTTCCAAGTTCTTATCCTGGAATTCCAGCTGCTGTTTATTATGCTATGCCTTCAATTCCGATAATAAAGGCTTGAGTTGATTTT<br>TGGGAGATAATGCTATGGCTTGGCATTCGAAAGGTTCGGTTTCCGTCACGCTGAATTCCGAAGCGGTGTTAGGAAACGCTACTGATTTTATCGCCAATGTGCGAACCGGCGATGCGTTCCGTGGCCCTGACGG<br>CCGGTGGTACGAAATCACCAATGTGACCAGTGCAACGGTTATTTCTATCAAGCCCAATTATCAGGGTGCGACCGCTAGCGGCCAAGTTTATGCGGTTGTCCCAATACATGGTTATTCAAAAAACTTGGCAGAC<br>CAATTCCGCGACATCAACAACCAGTGGGGGGCCACCCTGGCAGGGATCAAACCCTGG
Staphylococcus aureus<br>GATAGAGAAAATAAGTGCGAATATTTTATAAGAAAACTTAGGTACGTGTTTCTTAGTAAATGTGGCGCAAGCATTGACGAGTCCTATACATGTTGTTAGGCATGCAAGGATAACCGTCATTCCAAATACGAGG<br>TTACCGAACGAACCAAATAATCGTAATGAGTTGTACGTCAATATATCTGTACCATCTTTAAAGTTTCCTGGAGCTGTTGATGCCCCAACGTATGCAAGTGCAAAGTAAATCATTCCAAGTAATATGGCTGCAA<br>TAAGACCTGAAAAGCAGACATATTTTAAAATTTTCATGCGATCTGTGAGGCCTTTAAACTTATAGCCATTGACAATGACTACGGAAAAAGCTAACGCAGCAACAAGATCCATTGTAAAATAGCCTTCCAAACT<br>TCCTGAAATGAAAGGATGTGTTATATATTTATCCTTAGGTGCACTTAGTGCAGATTCAGGGTTGAAAATGACAGCAATACTTAATAGAGCGACCATTAATAGTAATAACGGTGTTAATAATTTACCTAAATTA<br>TCAACGATTTTCGATGGATTTAAACTAATCCAGTAAACGATGGCAAAAAAGATTGCTGCGAATATAATTAAAGTCCATTGGTTATGCACAGGTAAAATGTGTCTTGTACCAATTTCGTACGCGACATTTGCAG<br>CACGTGGAATACCGTAAAATGCTCCGATAGACATGTAAATCACGACAGCAAAAATAAACCCGAACCATGGATGTATACGATTGCCTACACTTTCAACACCTTCATCATAAAATGCAACAACAATAACAGTAAT<br>AAAGGGGAGTAATATGCCTGTAAGGGCAAAGCCTAGCATACCAATCCACATATTTTGACCCGCTGTATGGCCAAGCATGGGCGGGAATATTAAATTTCCGGCTCCAAAAAATAGTGAAAATAACATGAGGCCC<br>GAAATAATAACTTGTTTTTCAATGTAATTTCCTTCCTAAAACTAGATATTCATAATAATTTAAAAAATCTGAAAAATAAAAACGTTCTTACTTTATCTTTAAATGACAATACTATGATTCTATTATTTTATA<br>AAAAAGATTGCAATAATAACGTCTTGCTATTATTGGATAATGACAATTAAAGTGTCGTTTTTATGAATTAAAATAGGTGAAAGTAAGTTGGAAATACGTTGGAGCGTTGTGATTTTTATGAGAAATTGTATGA<br>GTGAGGACTGCTATTTATAAAAGGTAAACTATGGATGAGTTTATGGTTGTTCCAATAGGTAAATAAGAGATAGCACACTGTACACATAATGATACGTGGATGAGTGAATATGTCTGATAGGAAGGCCTAGTCA<br>CTCAAATGAATTTAAGTTTATTACGTGATAAA
Salmonella enterica<br>CGATGCTCGCGGCGGGTCAGATCGGGCAGTATCTCGCTATGTGCGGTTTTCCCCCCCAGTTCCGGCGAGAGCAGTTGCAGGATATCGGGCATTGATTCCATCGCCAGCCAGTGCATTTCACCCTCACCGGTCG<br>TGTCCAGCGTATCCAGAATGGCATCAATACCGGGCGCGCCTGCCACCATATCCTGTAACGTGTCGGTGTCGCCCTTTTTATCGTATAACGACGTCAAATCCTGGGTGACAGCGTCTTCATTTCGGGGAAACGG<br>TTTAGCACGGAGGGGCTTTTCCTGCCCGGAGGGCCGCAATGCCTGCTGATATTCCTGGTAAAGCTGGTGTAGTGTGCTATCCGCTTCATGGCCAGTGTAAGAGGATGCGGTTTCCCGGACGGGAATAATATCG<br>AACGGGTTCTGCGGATTGAGCTGTTGTTGTTTAAACCAGTCCAGATCCAGGTACTCAGCAACGGATTCAGATGACTGAACCAACTGAGGAAACGAAACATCGGGACGGGACTCATCATTGGTGCGGGCAAGGC<br>ACCAGGATGACAGACCCCATTCAATCGTATCTCCATCATTCAGCCGCATACGGTGATGAGGTTCCATCACCTGCTCATTGACGGCACAGCACAGTTCATCAGAGTGATTAACAATCCACCACGCCTCTTCGTG<br>GCGAACAAGACTGAGCGCGATATCGTCTTTCCCATGTTGCTCAAAAAGCGGAATGTAGGGGGCGGCGGCAGTAAATACGATGTATGTCCCTGCGGGATACTGTGCGTTATCCGTTATACCCTGCGACTGTATT<br>TTCCGTAATTCCCACATGTCTTGTTTCATTATGTCCCTTAAATGTATTTATTTTTCTGGAGGAAACGTTTAGGGAGTTTTAATTCATAAATAATTTTAACTAAATTTATAGGGAGTCATTATTGATGACACCC<br>TTTTTATTATTTTCGTCGTGAATGCATTGTTGTATGCATAGATGTCTTTTTTGAAATATTATTTCTTTTAATTCTGCAATTGCGATTTTTATCTTGCTGTTCATTGATTATTTAATATTAACGGCTTAGTTGC<br>TAATTAGTCCCTGATCTGTATCATTGTTTTGTTTCGATATTTTTTCGAGGCTATCAATAAAGAATTGTTTATATATTTATATGCATTGATGCATTATTTTTATGAATTTTTATGTCACAAGGCATAACACATG<br>GAAACTCCTGTTTCACGCAGTGCGTTGTATGGAAAACTGGCCGGCCCACTATTCCGGTCGCTGGAATCGGCAACGGCATTTTGCAAACTACGCTCTAATCCCTGGGTTGAGCTGACTCACTGGCTGCACCAGT<br>TAACACAGCAGCCCGATAACGATATTCTCCACGTTCTTCGGCATTACC
Proteus mirabilis<br>TGCAGCCGCCATATGCATAGCTACAGAATCAACACCAATAAAAAGAGCAGCATGATCAATTAATGCCGCAAGCTGAGAGAGTGATGTTTTTCCTGCTAATACATTAATTGATGTTTTATCAGGACACTGAGCT<br>AAAATATTTTCTATCATCTCAAGCTCTTTCGCTTCTGGCCCTGATGTTAACACAATTGAGTAATTATCTTGCTGAAGCTGAGTAATTAATGTCGCCATTTTATCTTCATCCCAACATTTAAAAAACCAACGAG<br>AGGTTGGTTGAATAACAATATAGTTTTTAGGGATATGATGCTTTTTAATTGTCTCTTCTAACCATTGTTTATCTTGGTGGCTATATGCCATGGTAACATCAGAAATAATTGGTACATTAAGAGGGGCTAGAAT<br>AGAAAGATTTTGCTCTACAGTGTGTAATTGTCCATGCTCTGCAGTAGGGACAATAATGTTATGACATTTTTGCCAAAACTTACTATTTTGCCGTTTTTCAAATTCAAACCCTAGGCGCACTGGGGCTTTGGTA<br>ACTAAGGCGAAAATAGCGGCTTTCCATTGGTCAGCTAAATTCACCACTAAGTCATATTGCTGTTTTTGTAAGATTTTCCCTAGTTGCCACTCTTTTTTTAATCGTGTGAACTTACCTTGATATTTCCAATTTC

GGTCAAGATAAAAAATATGATTTATTAATGGATTATGTTCAAGCATCGGTAATGTTTCTTTGTATAATAAAACATCAATCTTTGCATTAGGATAAGCTGATTTTAACGAATTAATAACAGGTGTCGTTAATAA
CATATCTCCATGATGTTGAAGTTTAATAACCAGTATTCTGTTAAATGATTGATTTATATTATTCATAATTACTTTAGCTTCAATGAGATACTTTAATTGTAAACAAATCCGTAGAATGCATCTACCATACCTT
GTTTATATTATTTGAATTGACGTTTTCTACTGCGTAACATAAGTCAATTGATACAATTATGAGCCTATAATCCATAAGTTGACGGGGAATGCATGAAAAAACCAGCATTTATTATCACATTAGATACCGAAGG
CGATAATCTTTGGGAAAATGAGCATAACATTACTACGCAAAATACGCATTTTTACCCCGTTTTCAGCAACTTTGCGAACGTTTTCAATTTAAACCCGTTTGGCTAACCAACTATGAAATGGCAATAGATGAA
GCCTATATTGAGTTTGCACAAGATGTGATTGCCCGTAACACTGGGGAAATTGGTATGCACCTTCATGCATGGAATAGTCCCCCAATTGTTGCGTTAACGGACAATGATCTTCATTATCAGCCTTATTTAATTG
AATACCCCAAATCGCAGATG

Streptococcus pneumoniae

GGAAAAAATCGGAACAGAGGAATGGCGACAACTTCAAAATGAAATTGGGCAAGCAGAAGTCGAGGTGTTAAAGATAGATAAAGCCATGGGGAATCTAGGGGATTCGAGCCGTTCAGCAACAGGAAACATCAAG
GAAGCTACAGGATACTTAAAAGCCGATGTAATGATGAACATTGCTGAAAAGGCAGGACAACTAGGTCAAAAAATGGTTGATGCTGGTAAAAAAACAGTAGGTGCATGGTCTGAAATCGACGAAGCGATGGATA
CTGTTACGACGAAGACTGGACTTACTGGCGAAGCCTTGTTAGGACTTCAGGAAATTGCAAAAGGAATCGCTACATCCTTACCATCGGCTACATTTCAAGAATCTGCTGACGCAGTTGGTGAGTTAAATACACA
ATTTGGACTTACTGGTGATACTTTGCAATCTGCAGCAGAGTACCTATTGAAATATTCGAAAATAACTGGAGAAGATATTTCAAATTCCGCAATAAATGCCAAGAAAGCAATTGATGCTTACGGTTTATCTAAT
GAGGATCTAGCGAGAGTATTGGACTCAGTAACAAAGGTCGGCCAGGATACTGGTCAATCTTATGACTCCATCTTTCAAAAAGCAATTGATGGAGCTCCACAGATTAAGATGCTAGGATTATCTTTTAAAGAGG
GGGCGACATTAATTGGTAGATTTGAAAAAAGTGGGATTGACTCTTCTGCAGCTTTAGCTTCACTTTCAAAGGCTACAGTAAACTATGCTAAAGACGGAAAGACATTGACTGAGGGGTTGAACGAGACTGTCAA
TGCAATTCAGAATGCTACTAGTAAGACAGAAGCAATAAGAATTGCTTCTGAAGTTTTCGGAAATAGAGCCGCTCCTAAAATGGTAGATGCTATCCAACGTGGGGCATTTAGTTTTAATGATTTAGCTGAAGCA
GCACAAAACTCATCAGGAACTGTAGCAACAACATTTGCTGAGACAAAAGATCCATTTGATGACCTAACAACCTATTCCAACAAAGCAAAAGAAGGGCTTGCCGAGATAGGTGGTACACTACTTGAGACTGTTA
TACCAGCTTTAGAACCTTTGATGGGCATGCTTGAATCTGCTGTTAATTGGTTTACCAGCTTAAATGAAACTGATCAACAGACTATCGTGATTCTTGGCCTCGTTACAACTGCTGTAATGCTACTGCTTGGTGC
AATAGCACCGCTAGTCATTGCTATAGGTGCAATAGGTGCGCCTGTCGGAATTGTCGTAGCGGCAATAGTTGCTGCTATTGCCGCTATTACACTCATCATTCAGGCCATCATGAACTGGGGGACCATATCTGAA
TGGCTTCAGTCGACGTGGGATGCTTGCGCCGCTTGGCTTTCTGAATTGTGGACTAATATTGTTACGACT

Staphylococcus epidermidis

TGTACCTCGTCTGACAATGTTGCTGCATGTATTACGTAATGCATTACAGGTAGCCATATCTATTTTTTGTATGTCGATACCAATGATTATGGGAGGTCTAGTCGTTATTGAAAATGTATTTGCGTGGCCGGGT
TTAGGACCATTAAGTATTAAAGCGATTACTGAACATGATTTTCCAGTAATTCAAGCATACGTGCTCATTGTCGCGGTGTTGTTTATTCTTTTTAATACACTTGCAGACATCATTAATGCAATATTAAATCCGC
GTTTAAGGGAGGCAATGAAATGATTGTGTTACGTCGTCTATTTCAAGATAGAGGTGCCATATTTGCTATAGCTATTATTACAATCTACGTAGTGCTTGGAGTTTTAGCTCCTTTAATTACATTCTATGAACCG
AATCACATTGATACAGCAAATAAATTTGCTGGTATAAGTTGGTCTCACTGGTTGGGAACAGACCATTTAGGTCGAGATGTATTAACACGGATAATATACGCCATAAGACCTAGTTTGTTATATGTATTTGTCG
CATTGATTATTTCCGTTGTGATAGGAGCGATACTTGGGTTTATTTCAGGTTATTTCCCAGGTTATATCGATGCAATAATTATGCGTATATGCGATGTGATGTTAGCTTTTCCAAGCTATGTGGTCACATTGGC
ATTGATTACGTTGTTTGGCATGGGTGTAGAAAATATTATTATTGCATTTATATTGACTCGATGGGCGTGGTTTTGTCGCGTGATTCGAACCAGTGTAATGCAATATATTGAAGCTGATCATGTAAAATTTGCC
AAAGTAATTGGTATGAATGATTTAACAATCATTCGCAAACATATTTTGCCACTAACCTTTACTGACATAGCGATTATTGCTAGTAGTTCGATGTGTTCAATGATATTACAAATGTCAGGATTCTCATTCCTTG
GATTAGGTGTTAAGGCACCTACAGCCGAATGGGGGATGATGCTTAATGAAGCACGAAAAGTAATGTTCACACATCCTGGAATGATGATGACAACAGGTGTGGCTATCGTCATAATTGTGATGGCGTTTAACTT
TTTATCAGATGCTTTACAAATGGCGATTGATCCTCGTATGTCCGCTAAAGAAAAACGACTGGCTCTGAAGAAAGGTGTGAAAGCACGTGACACTGCTTAAAGTAGCACATTTAGGAATTAAGGACACATGGTC
AGATGAGACGCTCGTTGATAACGTGAATTTCACAGTTCAATGTGGAGAAACATTAGGTATTATTGGAGAAAGTGGAAGCGGAAAATCGATTACTTGTAAGGCATTAATAGGTTTAAACGCTCAACGTTTATCT
GTAAGCGGAGATATCTTTTTTGAACATCAAAATTTAAATAC

Enterococcus faecalis

GTCAATTACTGGAAATGGTTCAGAAGTCGTTCATGGGGATGACAATGGCGATGTGGTCGTTGACATTGATCACAGTGGCGGTCATGCCACAGGGACTAAAGGCAAAATTCAGCTGAAGAAAACAGCCATGGAT
GGGACGACTATTTTAGCAGGCGCCCATTTCCAAATTTGGGACCAAGCCAAAACACAAGTCCTACGTGAAGGTACAGTAGACGCCACTGGGGTTATCACATTTGGTGGGTTGCCGCAAGGGCAATACATTTTGG
TGGAGACAAAAGCACCAGAAGGTTATACAGTTTCGGACGAATTAGCTAAAGGCCGAGTCATTACTATTGATGAAGAAACTTCAGCCGAAGGAGCACAACCAACCATTATTAAAAACGATGTCAATAAAGTATT
TTTAGAAAAAATGGATGAGAAGGGTAAAAAGTTAGTCAATGCTCGCTTTAAATTAGAGCATGCCGTAACCACGCCGTTTACTCATTGGGAAGAAGTTCCCCTTGCGCCGGATCGAACCAACGCGAATGGCCAG
TTAGAGGTGGATAGTTTAAAACCGGGGCTTTATCAGTTCACAGAAATCGAAGCACCGACAGGCTATCTTTTAGACACGACACCCAAACGGTTCATTGTGACACAAAACACGAGCGGACAAATTCATGATGTTC
ATGTTAAAATGCTTAATTACCAAGGTTCTGCTGAACTAATTAAAAAAGACCAAGCAGGCAATCCATTAGCAGGTGCTGAATTTTCAGTCCTTGATACCACAGGACAAGCGGTTCGAGAACACTTAGTTTCGGA
TGCAAACGGAAAAGTCACAGTGACGGATTTAGCCCCAGGCAAATATCAATTTGTGGAAACCAAAGCGCCAGCAGGGTACCTTTTAAACACTGAACCAAGTGCTTTCACGATTGCAGCAAGCGATCGGGGCAAA
CCAGCAACAGTTATAGCAACGGCTAACTTTGTTAACTATCAAGGCGCAGCTAAATTAATCAAAAAAGATGTGAATGGACACTTATTAAGTGGTGCGACATTTAAAGTGCTTGATGCGAAGGGAGAAACGATTC
AAACAGGCTTGACGACAAATAGTCAAGGGAAAATTATTGCAGAGCACTTAGCCCCAGGTAAATATCGCTTTGTAGAAACCAAAGCGCCAACAGGCTATTTATTAAATACCACGCCAGTCCCATTTGAAATTGC
TGAGAAAAATGCTGGTAAACCAGCGGTCGTTGTTGCTAGTGACAACTTTGTGAATTACAAAGGGGCTTTCCAAATCGTGAAAACGAATAGCGCAGACCAACCATTAGCAGGTGCTGTTTTTGAATTA

Neisseria meningitidis

AACGCAACAAACAAATAAAGCACGAAAACGGGGTTGAAGCCCATACCGCCTCCCTTAAACAGCCTTTAAACGATAATTGACCTTGAGTTAATACGTTTAAAGGCTGCTTTTTATGGCAAACGGGAACATGAAA
CTGTCGTTGGTGTTAACCGCCCGAGATGACGGAGCGAGACGGCTACTGGCTGATACTCAACGACAATTAGATCGTACCGCGAAATCGCGGGCGCAACTTGAACGGCAAAGCCATACTTATGCGTTGACCGGCA
TCCGCTCAGAAAAACAGATTCAACGCGAAATCATGCTGACACAGGCTGCGTTTAACCGTTTGGCGCGCAGCGGCAAGGCATCACAAAATGATTTGGCACGGGCGGCGGTCGCTACGCGTAACCGAATTCGCGA
GCTGAACGCGGAACTGAAACAGGGCACGGGATTTGCGGACAAGATGGGAAAAATCGGAAGATTCGGTGCAGCTGCGGTGGCTGGTGGCGCGGCAGCGTATACGGTGCTTAAGCCTGCTATGGACAACAGAAAG
CAGCTTGATGAGAACATCAACCGCGTGTCCAGACAGGCATTTATTGAGGATAACAGTAAATCGGCAGCGTGGATTGCAACTGAAGGTGCGCAACAGATCAAGGATTTGGCACTTGAACTTGTCGAGAAAAATG
GCGGGACCCACGATAAGGCTTTGGATTTAATCAGCGGCATGATGACCACCAGTCTGAATTTTGCCCAAACCAAGAATGAAGCGCAGGCGGCATATGCTTTTGCACTTGCCTCAGAAGGCAGTGGCGAGGATAC
GGCAAAACTGATTAAAACCCTGAAAGATGGCGGCATGAGCGGTAAAGACCTGCAACTCGGGCTTGAGCACGTCTTGCAATCGGGTTTAGACGGCACTTTCGAGGTGCGGGATATGGTTCGGGAGCTGCCGAGC
CTGCTCCCTGCCGCGCAACAGGCAGGGATGAATGGTGTCGGCGGTTTGGACTACCTGCTCTCACTCTTACAATCTGCGGCGAATAAATCGGGCAGTCCTGCCGAAGCGGCGACTAATGTGCAAAATCTTTTGA
GTAAAACTCTGTCGCCTTACACGATAGGTCGTCTGAAGAAGATGGCAAATCCAAATGACCCGAAGAAAGGTGTCGATTGGATAGGCTCGGTTGTGCAAGGCAAGCAAAACGGCGAAAACGCAGTGCAGGTGTT
GTCCCGTCTTGCCGATGCCATGCTAGTAAAGGATAAGCAATACCAAGATTATAAGAAACGCGCGGCTGCAGGCGATAAGACGGCGGCGGAGCAGGCAAATATGCTTAAGGGCGCGCTTTTGGCGCAACTGCTG
CCTGATTTGCAGGCAAAA

Clostridium sporogenes

ATACAGTATGCGCTATGATGATAACAAGGCTTGTGGAAGGAAAGGATTGGGTTGAGAAAATTACAGCTTAGAAGAAAGTTATACTACAGCCTATTAAAAGGGATATGAACAGCTTTAAAATATAAAATAATAT
AACACATCTTTAAATATTAATCATAATATGCTATTGGAGTATAATTTTAGGAGGAATTTTAGTGTACATTATGCCAAGAGGTGAAATTCCAATGATGAATAATATACAATTCCAAGAAGCATTGGAGGAAATT
AAAAGAGCAGTTCAGCATGAGAGAAAAGATGAATTATTTAATGAGTACCTTATAAATATTGCTCCTACCAGGGAAGAAGAGAAAATTATAGAGTCTATAAGAGATGATGAGTATAGGCATAATAAATTGCTTA
GAAGAATTTATATGGATTTTACAGGAATGGAAGTACCTATTGATGAGGAAGAAACTATTGCAAACCCAGAAAACTATTTAGATGGTATTAAAAAAGCTTTATTTAGAAAACTAAGAACAATAGAAAAATATAG
AGCTTGGAATAGAGGACCTTACAAAGACATGGTGTCTAATATAATTACAGATGAGATAAAGCACTCATCAAAGTATAATTATTTATTTGCATTAAATAGTAATATGAAGGTTATGGATATGAATAGAAGCCCT
GTGCAGGATACTTCTAAATTCACACCAGACGATTGGGTAAGATATATAACTCCATTAGTAAATCGAGCTTTAGCAGAAGCTAAGGCAGGAATTAACCCTGAACATTTATATCAGGAATTTATTTTAGCGGGTG
TACTTGTTGGACTTGGAAAGACTCCACAGGAAGCCATCGATCAGGTTGAGGAATGGGAAAATACAGGTGAATCACAGTTGCTTGCAAAAAGTAAAATGGCAAGAGGGTTTTATTAATAAATTAAAAGTCTCAA
AAGTAATAAAAGGCGATACCATTAAAATGGGATATCGCTTTTTATGTTTCAGAGTTTTTTATAATTATTCTTTAAGTATTCCCACTTTAGGTAGTTTTTCTAGTTTGCTTGATATTCCATGGCAGGTCTGAA
ATTCTGAAGTTAAGTTTTTACCAGCGGTTAAGCCATAGTGTTTTCCACCGCTCCATTTTGATATATTAGTCACATCATATACAATTCCATTAACTGCTACATAAGCCGGTTTTCCCATGGTACCGTCATATTG
AGCAAGTTCATTTAAAGTAAATTTCCTTGTTTCCCTACAATCATTTAAATTAAATCTTTCTTTACTAAAATCAGTATCAAGGTATTTAGCAACTTGAAGTGCTTCATCAGTTGTAAATTTAGTTTTAGAAGAG
GAAGTAGTATCTTTATATTTCATC

Legionella spiritensis

CCGGGCTGAAAGATGAAATACCGTTTGACGCCATAATAGATGATTCAAGAGATTTTTATGGCTTATTGCTATCATCTTGGGTTCACTGGTAGTCCCTGACGTTGAAATCACGTAAGCCATTTTTTCGCAGGAC
AGACTCTGACGTCTGCCCCTGGCTGGAGGTGGTGCATCGGAAGTCTTGCAGGTATCGATGTTTATCAATCGACAGGGATCCGCTTTTAATGAAGAAAGCCTTGCAAGATGTTCGGTCGATGTAATGATTGTAT
TGATATCGCATTGTTGAACGATCTTGTCTATACGCGTCACAGGCATTTGATGGGGGTCGATAATAACGAACGCGTTATCGGAGGCCAGTACCGCCAACAGGGCATTGATAAGTGTTGTACCGCGTTCAAGGAC
AATGGCGATGATTTTGTCATGGATGGTGATGTTTGCCGAAAGTGCCAGACTTTTTTCATGGAGTTCAGTATAGGTTATGGACGCGGTATTATGTACAATGGCAATTTTATCCGGGTATTTCAAAGCCTGGTGT
TGAAATACATCAAAGAGATCACCGGCTGGCAAGGTAAACTCCGGTGCCGGAGCATGTAATGGTAATAGAGATGACTGCTGTGAATCTTTTAATAATGGAATGTCGGCAAGGCGAATATCCGGATTTGATAACG
CCGAATCGATGATATTCAGTAAACTGCGTTTGACTTCGCGCATGAAATCATAGGAAAACAATGATCGGTTATAGTCCATCGTAACACCGAGCCTATCTTCTTTATGGGTGAACGAAAAACTGATATCAAATTT
GGCATAGGGGTGAGCTATCTTGTACGGTTTAACATCCAAACCATCAAGACTCAGTGTGACGGCTTCATCATCGCTAAACCAATTGAGTATAACCTGAAAAATTGGATTGCGAAGGGGATCACGATCATGCTCG
ACGTCGGTCACTATCTTTTCAAACGGCAATAGTTGATTTTCATAAGCTTCAAGACAAACCTTTCTGGACGTGCCCAGAAAATCACAAAAACGGTCATCGGCTGAAAATTGATTCCTCAAGACTAATGTATTGA
GCAGGCATCCAATGATCTTGTCGGTATCAGGGATCACCCGTTGTGTGATTGGCAAGCCAATCACAATGTCCACCTGTTGGGTGATTTTATAGAGCAGGATATTGAGCAGACCATACACCACCATGAATGGCGT
GCAGTGTTCCTGTTCGGCAAGTTTATGGAGTGCTTTTAGTCTTTTATTTCCTAAATAAAAATGGAAGGTGTTGCATGAAAATCCGGTAAAGTCCGGTGAGTTCTTATCATAGGGCAGCGAAAGGCGTTCATAA
TTTTTCAGTTTTTCCAACCAGTACTGGCGTT

Listeria monocytogenes

TTTCAAAACGAAGGAAATGCCCGCGAATGATGTCACTCTATATGCACATTTTACCATCAACAACTACCAAGCGAACTTTGATATAGATGGGGCAGTAACAGAGGAAGTAGTAAACTATGACGCGCTTATTCCT
GAACCAACATCACCGTCCAAAACGGGATTTACTTTCGAAGGTTGGTATGACGCAGAAGTTGGCGGAACAAAATGGGATTTTAAAACGATGAAAATGCCTGCGAACGATATTACTTTGTACGCACATTTCAGTA
AGGAAACACCAATTATTCCTAGTCCAGATGAAGGATTAGACTCTGATTCTACCAATGGACCAATTACTATAAATGAACCGAGTGCTACTAGTACGCCATCCCAAAATAATAACATCACAGTAACAGCAGGGGA
AAATACTACAGAACTAGCAACAGCTAAACTTCCAAAAACTGGAGATAATGCCCCGTGGAAAACATTGTTTGCCGGGATATTACTTTCATCATCCGCGTTTTATATTTGGAGAAAAAAAGCATAATTAAAAAAC
CCAGCATTTTCTAAAATGGAGAGTGCTGGGTTTTTGGTTTAATCAAGAATCTCAATATAGCCTTCTGTACCATTGATGCGAATTTGCTGACCATCTTTTATAACGGTAGTTGCATTTTCGACACCCACTACAG
CGGGAAGACCGTATTCGCGGGCAATCACTGCTCCGTGCGTCATAAGCCCGCCAACCTCGGTCACAAGCCCTTTAATAGATACAAATGCGGGAGTCCAACTAGGGTCAGTATAAGCCGTAACTAGAATATCGCC
ATCTTCTAAATCCACTTTTTCCATATTTAAAATGATGCGAGCGCGACCTTCAACTGTCCCAGATGAAACAGGAAGTCCAAGAATGGCATCTTTATGGAGATTTTCGCGTTTGTATTTGCCGTTAATCATTTCC
CCATCAGAAGTAAGGATGCGCGGTGGTGTTAATTTTTCAAAAGTGGCGAAATCGCGTTTTCGAGCGTTAATAAGTTCGTAATCAACATGACCAGTACGCACAACTTCCCGCAGTTCCTCGAAGTAAAGAAAAT
AAATATCTTCTTTATTTTGCAAAACGCCATTGTTTACAAGTTGTTCGCTAGCTCGAAGGAAAGCCAGTTTATAAATAAAGTAGCGGTTAATCATGCCGTATTTTGGATACTCGCGATAGCCAATGAAATGACG
TAAAATATCAATTTTTTCTTTTGTTTCCATCGCTTTTTGTTCCCCGTCCGGCAGTTCTTGCAAGCGCCTTAGGATTTCATGTTCTTTATCGAAAGCTTCTTTTTCTCCTTGAGCAAATTTGTGCATACTCGCG
CCGGGTTCAA

Staphylococcus capitis

CATCTACACCATACTGATTTAAACGTCTCATATCATTAACTTTATTTACTGTATACGGGTGTACTAGGAAACCTAAATCTTTTAAGTGTCTAGTATTTTTTATATTCAAGTCCGTGTATTCTGGACCGACACC
CACTGCATATGATTTAATCGCTTTTAAATCTTGTTCTGAAGCGTTTGTTAATTCGAATTTATTCATTAGTTTAATTAAAGGTACATTCGGATTCATTTGATGTAATTTTTGTAAGCTATCTCCGGAAAATGAT
TGCAACATCACATGTCCATTTTTTAATGAACTATTTGTTAACATACCATGTTGATTTAGAGTATTAAGTAATTGTTTTTCCATTCCTGGATAAACATTCGGTGATTTTGTTTCAATATAATAGTTGGCATTTT
TGCCATATCTATCTAAAATTTCATCTAACGTAGGTACTTTTGCATTTTTATAATTCTTGTTGGCATATTTAGGATTTGCTTTGTTAAACCAACTTCCAGCATCAAGCTGTTTTAATTGTTTTAAAGTATAATG
ATCTACGCGTCCATGGCCATTTGTAGTACGATTTACCGTTTCATCATGCATGGCAACTAAATGACCATCTTTCGTACGTTGTAAATCAATTTCTATGTAAGAGGCGCCTAATTCATTATGACTTTTATCATAA
GATTGAAATGTATGTTCAGGTGCATAACCACTTGCACCTCTATGAGCTATAGTTGTATATTTTTCTCCTGTAGGATTAGTAGTCCAGTGAGGATGTGAATCGTTTGTTTTTCCACTTACTGAATCAGATTCAG
GTTGACGTTTAAAATGATTATCTTGAGTTGTATTAGTTGTTTGTTGTGTTTGACTTTGATTGGCTTTAACTCCTCTAGTATTAAGGAAGTTAGAGGTAGTAATACAAAGGATACCTATAAATAGCACGACCAA
ACTCGTCAAAACTATTCTTTTAGATTTGAACATAAAAACATCCTTTCAAATTGATGTGATAAATATAAATTACACCATTTTAAAAGGATGAAAAAGAGAATGAGAGAAATTTTATATAAAATTTACTAACTTC

AAATATTATTAAAAAACTTAATTAATATTTAACAAATTTCGAAGATATCATAACAACATTCAATCATATTTCAAAGGTATTAAGTTAATAATGATTTTGCTACATTCAATTGATGTTTAACGGAATCTTGTCC
TGTTGAGCCGTAACTTTTACGACGTTTTAAACAATTTTCAGGATTTAAATAGTCATAAATATCTTCATTAATGCTAGAATGTTGTGCTTGATACTCTGATAGTGGTACATCAAGAAGATAAATACCTTTGTGA
ATACAATTTAGCACGATTTTACCAACAATTT

Streptococcus agalactiae

GCTTGAGAGAAAGTTTGGAGGTGGTAGAATGAGATGTTCAACCAAAGAAATGAGGCTTGTCTATCGTGATTTCCTCCTTCAAGCTAATCAAGAAAATAAACAAATTACCGTTTTAGAAGCCGATTTGTCGAGT
TCAATGTCAACAAATGTACTGGCATCAGAATTTGGAAAACGTTATATTAATCTAGGTATTATGGAGGCTGAGATGGTAGGTCTTGCAGCAGGGCTCGCCATTAAAGGTTACAAGCCATACCTTCATACTTTTG
GTCCTTTCGCGTCAAGGCGTGTCTTTGATCAAGTATTTTTATCGTTAGGTTACTCACAATTATCAGCTACGATTATTGGCTCAGATGCAGGAGTTAGCGCAGAAATGAATGGCGGAACTCATATGCCTTTTGA
AGAGTTAGGTCTACTAAGGTTAATTCCAAAGGCGACTATTTTTGAGGTTAGTGATGATATTCAGTTTGAAGCAATTTTAAAACAAACATTAAGTATTGATGGTCTAAAATACATTAGAACTATTCGTAAGGCC
CCCACTGCAGTTTATGAGGGATGCGAAGATTTTTCAAAAGGTTTTATACAATTACGCCAGGGAAAAGATATTGCTCTTGTAGCAAGTGGTATTATGGTTTCTAGAGCGATAGAAGCAGCCGACTACCTCAAAG
AACTAGGGATTGAGGCCTCAGTTATCGACCTTTTTAAAATTAAGCCCCTTCCAGAAGAACTGAAACCATTACTGATAGATCAATCCATTGTAACTATTGAGAATCATAACCGTATTGGCGGTATCGGAAGTGC
ATTATGCGAATGGCTTAGTATGGAGAAAGACACTACTGTTAGTCGTATGGGTATTGATGAGCGATTTGGTCAGGTAGGTCAGATGGAATATTTATTAGAGGAGTATGGACTCGCTGTTAAAGATATAGTAGAG
CATTGCAAATCAATTTATAAATCATAACAAAAAGGCAACATTGGAGTTGCCTTTTTGTTATTTTCTGAAATTTTTAAGTTTTGTATAAGTATCTTGTGGTATTTTATCCTTACTAAAAATAAAGGGGTGGTCG
TTATGCGAGCTTGGAAAGGTATTGTTTTAATTTTATCTAGCATAGTGGTGACACTAGTAGCATGGCAAAATGCAGGATTAAGTGAATTTGTTGTACCAGGATTAGCTTTGACAAGTTTATCGCTAACATTCCT
CTTATCAACCAAATTTAGGATACTTGAAAGTTACTTTCAAGGTATTGAAAATATGTATTTTTACCACAAGGTAATGGCTGTATTTTCAATGATTTTGCTTCTATTGCACAAAATTGGTCTTGTACAAGG

Chlamydia pneumoniae

CTTCCAGAAACGCAACAACAAACATCTCCTTTAACTTCACTAGAAGGGGGACATGAGGTAGCTCTAGTTCTCAATCCCCAGCAGAACCCAGAGGCTCTAAGTATTGCCTCTAGATTGATGCATGAAGAAAGAG
GTGGGAGATTAGAATCTAACTATATGCCTGGACGTTCTAGTAATCCTTTCATGACAAGTATGTATGTTCTCGTACGGCTGAATACACTTGCTCAGATCTACCTGATGTCTCCTTATTATTCTTTCCAAAGCAA
CGACATTGTATGCCTTATCTTTATAAGCAGTGCTGCTGTAGAGACAGTAAGCTACATATTCCTGACTGTAACTGACTCAACTTGTGGGCGTCGGTACCTGCGTGTCCCACGGCTAGTTTGTACAGGGTTACGT
AACCTGGCGTTACCCACAACTCTACTAGAGCTACTTATTTTGTCATACCCTCGATCAGTAGAGGGGGTACCTTTCAATGTTAGATTCATTCTTGGATATATGTGCACTACTAGAGTTGTATTTTTTGCATGGA
ACTTGATCCTCCACTGGCCTTTCCGATGTCTACGCCATGGAATCCAATTGTTTGTTCATAGAAGTATAATAGGACATACGTTGGGAGCAAGAATTACTGATTTAACCCTAGCAAGTATGCGATACGCAATAGT
GTTTCCATCTATAGTAAGTTCATGCTTGTTAACTGCTCTTGCTCATGCAAATACTAACATACTTGCCTTGGACCCTTATAGATTGATCGAATCTGGAGATTTAAGACGTCCCGCATTTAATGATGATGAAATG
CAACAAGCAGATAATCCTTGGGATGCTTACTCTATCGGCTTAGTTATAAACACGTGTATCTACATGTTAATTTTATTCGCAAACCTAATTTTCATGGTGTACTCTGTACGAAGATACCATAGATCCCGCCGCT
AAGAGTAGCTTGCCTTAAGTTTCGTACTATCTATTCTTCGGCAATGCAAGATAAGAAAACATTGATTAAGAGCGAATAGAACCCTAAAAAACTGTGTTTATTTTTTATTACAGTTTTTAGATATAAAGATCT
CTTTTTAGTTCCGTATCTCTAAATGAAATCAAAGGGTTCCTTGTGAAGAAAACCCTACAGATTGCAACCGAAGAATAGACTCGAAATGAGGGGACACCTTTGGGAATCTCTGTTCGTAGGTGCGTGACTGATT
AGAAAAAAAGTTTTGATTTTTCAAATGAAATAAGCCTACTTTGTATTAGAGGCTGATTCATAAACTTTCCTAAGAAATATATGATAAGAAAAACTTGGGGCGGACAGGATTTGAACCTGTGACCTACGGGTTA
TGAGTCCGCAGCTCTAACCACTGAGCTACCGCCCCCCAAGGTAAGAGAGCAATGCT

Enterococcus faecium

CAACCAAGTAAGTATCCAAGCGCCCAAGTACGCGGAGAGTTTCAATACTTCAATCTTATTGGTTGGCGCTAATTTAGGTACTTTCATTGCACCATATGGTTTGCTCTTTCTCTCTTTTGCTTCTCTTGGGAGT
AAAACACTTGCTGTTTTCGTCAATGGCAGTATCTTGCTGTTGATTAGCGCGCTTATTAGCCTTCTAGTAGTACACAGGACGAATAGAAAGACAGTGATAAGAGTAGATGAAGAAATAAAAGAACAATTGACAA
CAAAATAAATTTGATGGAGGTCGTGTAAAATGACAAATGAAAAACATAAAGATAGATGGCTTTGGTATCCTGGAGACTTTGAGATCCGCCATGGACTGCTACAAAATTTCCAACGGGAAGAACGAGGTTTTGA
CTGGCCAGCGTACTGGTACATGGATGATTGCCATCGGAACGTCAAGTTTAAGAGATATTACTTTCTTGATCAACCTTCCATGTTTAAAGTGACGATCCAAGGAGTTGGGTATGTAGAAATTAATGGACAAAAA
CATCCATGTGGAAAATGGCTGACTTGTCCAGCTGGTAAAGCAAAAATTCGTATCTTTGTAGGACATACATCAGGGCTACCAGCGATGTTTATCGAGGGAGACGAAGTAAAATCAGATATTGGCTGGACAGCAA
GTAATTTTATTGAAGAATATCCAGCTGGCTGGAGTCCGCTTTATGTCGATATCGCAAAAGATCCAAATCAAATCTATTATCAAAAAGAATGCGTCCATCCAGTCACTGAAACGGAGATTGGCGGAGGAATCTT
ATTCGATTTTGGGCGAGCAGTGAATGGCTGTGTAGAACTAATATCTCTGGATGACCATATCGAATCTATCACTCTATGTTATGGAGAGTCCGATGTAGAAGCTATGGATATCGATTATTGCTACTATAAGCAA
ATAGGAATGACAAGAAAAATGAAAGCTCGTAAACGCGCTTTTCGCTATTTGTTTATACCAGAGGTTTCTACTGGAATGGTTCAACTTCAAGTTTTTCATGAATTTTTATCTCTTTCGAAACAAGCTGCTTTTT
CAAGCGATTCTTCTTTACTGAATCAGATCTGGTCAGTATCAGAAGAAGCCTTCAAGTTATGTAGTGATTTGTTTTTTATCGACGGTATCAAACGAGATCGCTGGATTTGGTCTGGAGATGCGTATCAATCATT
CATGATCAATCAATATCTTTTTTTTGACGAGGAAATCAATAAACGAACGATATTAGCATTAAGAGGACAAAATGAAATCAAGCAGCATATGAATACTATCGTTGACTATTCGATCTTGTGGTTGATTGGTATC
GAAAACCACTATATGATGACGAAAGACTATGAATTTTTACGCATCGTCTACCCTAAGATGCAATCA

Staphylococcus haemolyticus

CTAAACAAAGGCGATAGAGTTATTAATGGTAAAACCACTCAAAAACTTCAAAATCAAGGCTTTATTCCTAAATTTTCTCGTGGTACAGATAGTGGTGAAGATGTTAGAAAACGTATGCTAAGAGAAGCTAAGA
AACGCAAGAAACATAATCATCCTACATTTGATGCTGGGGAAATGATGGGACAAGGTGGTTTCGGCGCAGGTGGTGCTGGAGGCGCTGCAAAAGAAGCGTGGAAATACGTTACTGACAAAACTAAAAATATAGG
TTCAGGTGCTAAACACACGGTTAAATCACTTAGTGACGGCGCTAAGAAAATGATTAACACTACTAAAGGTGCTTTAGGTGCTGCAGGAACATGGGCCAAAGAGAAAGCAGGCGACTTATTACAATATCTTGGT
AGTCCTGGAAAACTTGTTAATAAAGTCCTAAAAGAGTTTGGCGTAGACTTTAGTATGGTTAATGGCGAAATTCCTAAAATGCTTTGGGACGCAATGTGGAAACAACTAAAAGAAGGCGTTAAATCGTTATTTG
GCGGTTGGTTAGATGACGTTTCTGGTGGCGATGGCGACGGTCGATTTATTAAGTATCTTGATAACATTACTACAAGATACAGTCCGAACGGTCCACCACCAGGCTATCCGTTTAACTGGGCGCATCCAGGTAT
TGACTTACCATATATTTATGAAAAAGTCCTAACGCCAATGGGCGGTAAAGTTGAAACAAGACATACAGCATCAGGTTTTGGGAAACATGTTATCGTAAGAGCAAAACCTTATGATGCTTACTTTGGACATTTA
AGTAAATGGCTTGTTAAAAATGGACAACGTGTTAAACCTGGTGACGCTATTGGTATTTCTGGTAACACAGGTTCAAGTAGTGGTCCTCACTTACACTATGAAATGAATAAACACGGTTTCGGCTCAATGACAG
GTCATTCTATTGATCCAGTTAAATGGTTGAAGTCACATAATGGTAGTAAGAGTGTTTCTAAATCAGCAAGCGCATGGAAACCCGATATCAGACGTGCTGCTAAAGCAATTGGCGTTCGAGTATCTAACGCTGA
TGTGAACGATGTTGCTCGACTTATCCAAACAGAAAGTAGCGGTAATGCTGGAGTTACACAAGGGATAAAAGACGTAAACAGTGGCGGTAATGAAGCACAAGGCTTACTACAATATACACCAGGATCATTTAAC
AGTTATGCAATTAGAGGACATAAAAACATTAAAAACGGTTACGACCAATTACTCGCCTTCTTCAATAACACAGATTGGCGTGCTAATTTATCTTACTGGAAACGTCGAATGGCTAGTGGCTTAACTGG

Staphylococcus lugdunensis

ATACTGCTGAACAACAAGTCTTTCATCAAGAGTTGATTGAACGTGTCCAAACACCTATGACGCGTGATGAAGCAATAGATTGCCTTATTCAGCAATTGGATAAACATGGCTATGTTAATGATTGTTTAACTTT
AAAAGATGCTATTGTAGCGCGGGAAGCCGAAGCGACAACTGCAATAGGAATGAACGTGGCGATTCCACATGCCAAGTCAGCTGCAGTTAAAGAACCTGTTGTGGCAGTATTACAGCATCAAACAGGTGTAAAG
TGGGATAGTCTAGATGGTTCATTACCACAAATTATTTTCTTAATAGCTGTGCCAAATCATAGTAATGATACACATTTGAAGTTGTTACAACGTTTATCAAGAGCCTTAATGGACGATGCTACACGTAAACAAT
TTATTGAAGCAGCATCAACAGCAGATATATATCACATGTTACAACAACTATAAAGGAGGCGTCAGGACATGGTGTTATTTTTACAACCAGTTTTTCAAGAACGCATTTGGGGAGGTCGTGCCTTACAACAACA
ATTTAATTATGATATTCCAAGTCATACTACTGGTGAATGTTGGGCGATTTCAGCACATCCAAATGGTCCGAACATTATTGAAAACGGACTGCATAAAGGAAAGACTTTGACGCAAGTATGGGATGAAGATCGC
GCATTATTTGGAGAAGATCAACGCAGCCAATTTCCACTGTTAACAAAGATTTTGGATGCTAATGATAAATTATCTGTGCAAGTTCATCCGGATGATGCCTACGCTTTAGCACACGAAGGCGAGTATGGCAAGA
CAGAGTGTTGGTATATTATCAGTGCTAAGGAAGGTGCAGAAATTATTTATGGTGTTCATGCAGATAATCAAATTGAGTTAGCCCAGAAGATTGATGCACGAGACTTTGACACGTTATTTAAACACGTTCCGGT
TAAAGCAGGTGATTTTTTCTATGTGCCGGCTGGAACTGTACATGCCATTGGGGCCGGGATAACGATTCTAGAAACACAACAATCTTCAGACACGACCTATCGTATTTATGATTATGATCGCAAAGATAAAAAT
GGACAACTACGTGCACTTCATTTGGAACAGAGTAAGGCGGTTATTGATCTTGAAACGAAAAATCCTAATACGGTGCCGATACATAGTATCAAGCACGGCCAGTCAATGACACAGTTTGTGTCAAACGCATTCT
TTACAGTAGATAAATGGGAAATTGATGGCACGTTACCATATGAGAAACCACATGTTTATTGTCTAGTATCTGTGATAGATGGACAAGGTATTGTAGCTATAGATGGAGAACAATGGCCAATTAACAAAGGCAG
TCATTTTATTATCACAGCTGATGATTATGACGTTGAATTTGATGGACAACTCACGTTAAT

Supplementary Table 6. ATCC Bacterial gDNA Samples

Name	ATCC catalog #
Genomic DNA from Clostridium sporogenes strain 388	3584D
Quantitative Genomic DNA from Escherichia coli	BAA-2192DQ
Quantitative Genomic DNA from Escherichia coli	BAA-2326DQ
Quantitative Genomic DNA from Enterococcus faecium strain VRE	700221DQ
Genomic DNA from Enterococcus faecium strain TEX16	BAA-472D-5
Quantitative Genomic DNA from Listeria monocytogenes strain EGDe	BAA-679DQ
Genomic DNA from Neisseria meningitidis M1027	53417D-5
Genomic DNA from Pseudomonas aeruginosa strain PAO1-LAC	47085D-5
Genomic DNA from Salmonella enterica subsp. enterica serovar Paratyphi B strain MZ1438	BAA-1585D-5
Genomic DNA from Salmonella enterica subsp. enterica serovar Muenchen strain MZ1477	BAA-1674D-5
Genomic DNA from Staphylococcus aureus subsp. aureus strain SA113	35556D-5
Genomic DNA from Staphylococcus aureus subsp. aureus strain TCH1516	BAA-1717D-5
Genomic DNA from Staphylococcus aureus subsp. aureus strain TCH959 (ATCC BAA-1718)	BAA-1718D-5

Supplementary Table 7. Concentration of primers, probes and blockers

Species Name	Concentration (nM)									Blocker Selection
	Primer			Probe			Blocker			
	1	2	3	1	2	3	1	2	3	
Acinetobacter baumannii	15	15	19.5	60	60	78	30	30		Blocker S1T1, Blocker S1T2
Citrobacter freundii	30	45	30	120	120	120			30	Blocker S3T3
Escherichia coli	15	15	15	60	60	60	30	30		Blocker S4T1, Blocker S4T2
Klebsiella pneumoniae	19.5	15	15	78	60	60	30		30	Blocker S5T1, Blocker S5T3
Pseudomonas aeruginosa	15	15	15	60	60	60	30	30		Blocker S6T1, Blocker S6T2
Staphylococcus aureus	45	75	60	120	180	150	30	120		Blocker S8T1, Blocker S8T2
Salmonella enterica	15	19.5	30	60	78	120		30	30	Blocker S9T2, Blocker S9T3
Proteus mirabilis	19.5	30	15	78	120	60				None
Streptococcus pneumoniae	15	19.5	15	60	78	60		30		Blocker S11T2
Staphylococcus epidermidis	30	15	30	120	60	60	30			Blocker S13T1
Enterococcus faecalis	19.5	15	15	78	60	60			30	Blocker S15T3
Neisseria meningitidis	30	19.5	15	120	78	60	30		30	Blocker S16T1, Blocker S16T3
Clostridium sporogenes	45	15	34.5	120	60	138		30		Blocker S17T2
Legionella spiritensis	15	19.5	15	60	78	60	30		30	Blocker S18T1, Blocker S18T3
Listeria monocytogenes	15	19.5	15	60	78	60	30	30		Blocker S19T1, Blocker S19T2
Staphylococcus capitis	49.5	60	45	138	120	120				None
Streptococcus agalactiae	34.5	15	30	78	60	60			60	Blocker S21T3
Chlamydia pneumoniae	19.5	15	15	78	60	60	30	30		Blocker S22T1, Blocker S22T2
Enterococcus faecium	15	15	19.5	60	60	78	30	30		Blocker S26T1, Blocker S26T2
Staphylococcus haemolyticus	15	15	15	60	60	60		30		Blocker S27T2
Staphylococcus lugdunensis	15	15	75	60	60	60	30			Blocker S28T1

Supplementary Table 8. qPCR procedure

#	Stage	Temperature / °C	Time	Cycles
1	Stage 1	95	5 min	1 cycle
	Stage 2	95	10 sec	60 cycles
		60	20 sec	
		72	15 sec	
2	Stage 1	95	5 min	1 cycle
	Stage 2	95	10 sec	60 cycles
		60	40 sec	
		72	15 sec	
3	Stage 1	95	5 min	1 cycle
	Stage 2	95	10 sec	60 cycles
		60	60 sec	
		72	15 sec	
4 (Optimal condition)	Stage 1	95	5 min	1 cycle
	Stage 2	95	10 sec	60 cycles
		60	90 sec	
		72	15 sec	
5	Stage 1	95	5 min	1 cycle
	Stage 2	95	10 sec	60 cycles
		60	120 sec	
		72	15 sec	
6	Stage 1	95	5 min	1 cycle
	Stage 2	95	10 sec	60 cycles
		57	90 sec	
		72	15 sec	
7	Stage 1	95	5 min	1 cycle
	Stage 2	95	10 sec	60 cycles
		63	90 sec	
		72	15 sec	

Supplementary Table 9. qPCR thermocycling settings and primer concentration for modular higher-plex CCMA development

#	CCMA Primer concentration* / nM	168-plex primer concentration / nM	Total Primer concentration / nM	Input quantity (copy)	Denature Time	Annealing Time	Extension Time
1	15	0	3036	3000	10s	90s	30s
2	15	0	3036	300	10s	90s	30s
3	5	0	1012	3000	10s	90s	30s
4	5	0	1012	1000	10s	90s	30s
5	5	1	1415	3000	10s	90s	30s
6	5	1	1415	1000	10s	90s	30s
7	2	0	405	10000	15s	5 min	30 s
8	2	0	405	3000	15s	5 min	30 s
9	2	0	405	1000	15s	5 min	30 s
10	2	1.2	808	10000	15s	5 min	30 s
11	2	1.2	808	3000	15s	5 min	30 s
12	2	1.2	808	1000	15s	5 min	30 s

Supplementary Table 10. Ct values and ΔCt values of *Staphylococcus epidermidis* under qPCR condition listed in Supplementary Table 9.

#	Ct 1 (ROX)	Ct 2 (FAM)	Ct 3 (HEX)	order 2 - order 1	order 3 - order 2
1	25.0	27.5	32.6	2.5	5.1
2	28.0	31.1	36.9	3.1	5.8
3	29.4	41.1	47.9	11.7	6.8
4	31.1	44.0	50.9	12.9	6.9
5	29.4	41.5	48.7	12.1	7.2
6	31.0	44.2	51.6	13.2	7.4
7	23.2	28.3	35.7	5.1	7.4
8	25.7	30.7	37.4	5.0	6.7
9	27.8	33.5	39.5	5.7	6.0
10	23.8	29.3	36.2	5.5	6.9
11	26.3	31.1	38.3	4.8	7.2
12	28.8	34.3	40.8	5.5	6.5

Supplementary Table 11. Clinical sample information

Sample ID	Sample Type	DNA conc. / ng/uL	A260/A280	Sample Volume / mL	Eluant volume / uL	Equivalent clinical sample volume for test / uL
S1	Blood	70.2	1.81	10	100	200
S2	Blood	20.8	1.77	10	100	200
S3	Blood	134.9	1.87	10	100	200
S4	Blood	7.7	1.53	10	100	200
S5	Blood	130	1.85	10	100	200
S6	Blood	227.9	1.89	10	100	200
S7	Blood	222.9	1.86	10	100	200
S8	Blood	204.6	1.86	10	100	200
S9	Blood culture	51.2	0.26	10	100	200
S10	Blood culture	72.5	0.45	10	100	200
S11	Blood culture	30.7	1.8	10	100	200
S12	Blood culture	4.3	1.65	10	100	200
S13	Sputum	327	1.93	0.5	100	10
S14	Sputum	164	1.88	0.5	100	10
S15	Blood culture	501.1	0.51	10	100	200
S16	Blood	20.8	1.77	1	100	20
S17	Blood	134.9	1.87	2	100	40
S18	Blood culture	130	1.85	2	100	40
S19	Pleural effusion	3.9	1.54	5	50	200
S20	Sputum	29.896	2.15	0.5	100	10
S21	Sputum	21.658	2.16	0.5	100	10
S22	Sputum	46.338	2.04	0.5	100	10
S23	Blood culture	84.5	1.82	0.4	100	200
S24	Blood	12.3	1.98	10	100	200
S25	Colony	6.6	1.63	0.5	100	
S26	Pleural effusion	3.9	1.54	10	50	400
S27	Sputum	83.2	1.67	0.5	50	20
S28	Sputum	20.6	1.88	0.5	50	20
S29	Blood culture	124.9	1.87	0.5	70	20
S30	Blood culture	29.6	1.85	10	60	333
S31	Blood	117.8	1.64	10	60	333

S32	Blood	19.5	1.79	1	60	33
S33	Blood	14.5	1.73	1	60	33
S34	BALF	5.18	1.56	20	100	400
S35	BALF	3.1	1.44	20	100	400
S36	BALF	222	1.61	20	100	400
S37	Pleural effusion	162	1.87	10	100	200
S38	Sputum	106.2	1.66	0.5	50	20
S39	Blood	8.1	1.91	1	100	20
S40	Blood	4.9	1.99	1	100	20
S41	Blood	6.3	1.79	1	100	20
S42	Sputum	88.7	1.63	0.5	50	20
S43	Sputum	19.2	1.57	0.5	50	20
S44	Blood	5.6	1.88	5	100	100
S45	Blood culture	19.7	2.01	10	100	200
